# In-situ architecturing of 3D graphene networks in metals via laser additive manufacturing for strength-toughness synergy

**DOI:** 10.1038/s41467-026-77015-z

**Published:** 2026-08-21

**Authors:** Zhongkun Liao, Jingchi Zhang, Zichuan Fu, Liang Zhao, Xiaotong Luo, Fan Shi, Baisong Guo, Kaihao Zhang

**Affiliations:** 1 Smart Manufacturing Thrust, The Hong Kong University of Science and Technology (Guangzhou), Nansha District, Guangzhou, China; 2 Mechanical and Aerospace Engineering, The Hong Kong University of Science and Technology, Sai Kung, Hong Kong SAR, China; 3 Institute of Advanced Wear & Corrosion Resistant and Functional Materials, Jinan University, Guangzhou, China

**Keywords:** Metals and alloys, Mechanical engineering

## Abstract

Integrating two-dimensional nanomaterials into three-dimensional bulk metals to simultaneously achieve high strength and toughness remains challenging. Conventional fabrication methods for graphene-reinforced metal matrix composites (MMCs) often lead to graphene layers agglomeration and poor interfacial bonding. Here, we report a transformative manufacturing strategy that spontaneously architectures a three-dimensional graphene network within the metal matrix. Exploiting the rapid thermal cycles of laser powder bed fusion with a Ni/polymer precursor system, we drive in-situ conformal growth of high-quality graphene wrapping printed Ni scaffolds. Subsequent hot pressing densifies the pre-designed structure into a bulk composite, preserving the continuous, three-dimensional graphene network. This architectured composite exhibits a pronounced strength–ductility synergy, achieving a 72% enhancement in ultimate tensile strength (648 MPa) without sacrificing ductility (54% elongation). Our approach shifts additive manufacturing from a shaping technique to a potent platform for precise reinforcement architecture control, establishing a viable route to fabricate high-performance MMCs.

## Abstract (web summary)

A laser powder bed fusion (L-PBF) approach utilizing Ni-PMMA powders produces graphene-metal composites with a programmable 3D graphene network inside the metal matrix. This unlocks synergistic strengthening and toughening, overcoming the classic strength–ductility trade-off.

## Introduction

Metal matrix composites (MMCs) combine the advantages of both metal matrix and reinforcement agents, making them applicable in enormous fields such as aerospace^1,2^, rail transportation^3,4^, and power electronic devices^5–7^. Graphene, having a unique combination of extraordinary intrinsic mechanical and physicochemical properties, is an ideal nanoscale reinforcement for MMCs^8–10^. Large-scale production of high-quality graphene and effective interfacial incorporation of graphene within metal matrix dictate the success of high-performance graphene-reinforced MMCs (Gr-MMCs)^11^. However, the transition from graphene’s superior in-plane properties to Gr-MMCs’ macroscopic performance confronts a fundamental physical challenge: the immense mismatch in dimensional scales between two-dimensional (2D) carbon nanomaterials and three-dimensional (3D) bulk metallic composites. This scale mismatch persistently undermines the efficient translation of graphene’s exceptional intrinsic properties to the composite level, resulting in Gr-MMCs mechanical properties that consistently fall short of theoretical predictions.

In practice, initial research efforts to develop high-performance Gr-MMCs focused on achieving uniform dispersion of discrete graphene sheets within the metal matrix. Techniques such as molecular-level mixing^12^, ball milling^13–15^, gas-liquid reaction^16^, and electrochemical deposition^17^ have been employed to distribute graphene nanosheets within the metal powders then consolidate them together into a bulk sample. While this homogeneous dispersion strategy can locally hinder dislocation motion and crack initiation in the metal matrix, it often results in a significant loss of ductility for limited strength enhancement^18–20^. On the other hand, the graphene used in these approaches, often in the form of discrete defective nanosheets due to mechanical deformation or graphene derivatives like graphene oxide or reduced graphene oxide, contains inherent structural defects and a limited size that degrade its reinforcement coefficient and provide insufficient load transfer at the carbon-metal interface^21^.

To better harness the in-plane properties of individual graphene layers through improved spatial distribution, researchers have turned to bio-inspired architecture designs^22–26^. Recent studies on Gr-Cu laminated composites reported remarkable in-plane ductility of 5200%^27^ and notable compressive strength of 1.56 GPa^8^. For more complex 3D graphene network (3DN-Gr) architectures, the 3D continuous graphene-like phase interpenetrating the metal matrix can effectively activate strengthening and toughening mechanisms across multiple length scales by significantly improving carbon-metal interfacial shear strength, complicating 2D nano-reinforcement pull-out, and deflecting crack propagation, thereby facilitating more efficient energy dissipation while utilizing graphene’s in-plane strength^9,28–35^.

A promising method for creating such 3DN-Gr is the direct growth of graphene on metal powders via chemical vapor deposition (CVD) prior to consolidation. This approach provides improved graphene-metal interfacial bonding compared to ex-situ mechanical mixing^21,36,37^. However, the curved surface of powder particles often leads to graphene with discontinuity and inherent structural defects, as indicated by prominent D to G peak intensity ratios in Raman spectra^25,37,38^. More fundamentally, to fully realize graphene’s potential for synergistic strengthening and toughening, its 3D network architecture requires precise spatial control—a design degree of freedom not afforded by conventional powder metallurgy techniques. The need for precise architectural control naturally leads to the application of additive manufacturing (AM), which enables the direct creation of complex, pre-designed structures. For instance, Zhang et al. demonstrated this by creating lightweight Mg-Ti composites via infiltrating Mg into 3D-printed Ti-6Al-4V scaffolds architected with bio-inspired structures^39,40^. These interpenetrating phase composites achieved a superior combination of specific strength and fracture toughness. In a similar vein, Bauer et al. proposed a nanoarchitected approach, fabricating interpenetrating phase composites with 3D-printed pyrolytic carbon reinforcements featuring gyroid and spinodal minimal surface topologies, which were subsequently infiltrated with a Ni matrix via electrodeposition^41^. The resulting nanoarchitectured composites demonstrated compressive strengths up to 3.5 GPa and absorbed nearly five times more strain energy than its monolithic carbon constituent. A fundamental insight common to these 3D-printed architectured MMCs is that the 3D-continuous, mutually interpenetrating configuration of the reinforcement and matrix phases is paramount for synergizing the strengthening and toughening mechanisms. This specific architectural design promotes superior stress transfer, effectively delocalizes damage by forcing crack deflection and twist, and activates powerful extrinsic toughening mechanisms like crack bridging. As a result, this architectured heterogeneity, rather than the properties of the constituents alone, successfully overcomes the traditional strength-toughness trade-offs. This also provides a clear design principle for Gr-MMCs: using AM techniques to deploy graphene layers in similar or more advanced 3D architectures in a metal matrix. While laser powder bed fusion (L-PBF) AM has been used to fabricate Gr-MMCs, reported studies predominantly rely on pre-mixing graphene nanosheets within the metal matrix powder bed^42,43^. This approach is still confined to a homogeneous reinforcement distribution after powders melting, largely because commercial L-PBF systems are designed for processing uniform, pre-mixed powder feedstocks, with true multi-material L-PBF printing remaining a significant technical challenge. Consequently, current MMCs AM methods usually fail to exploit AM’s core capability for the spatial control of material composition and microstructure.

**Fig. 1:**
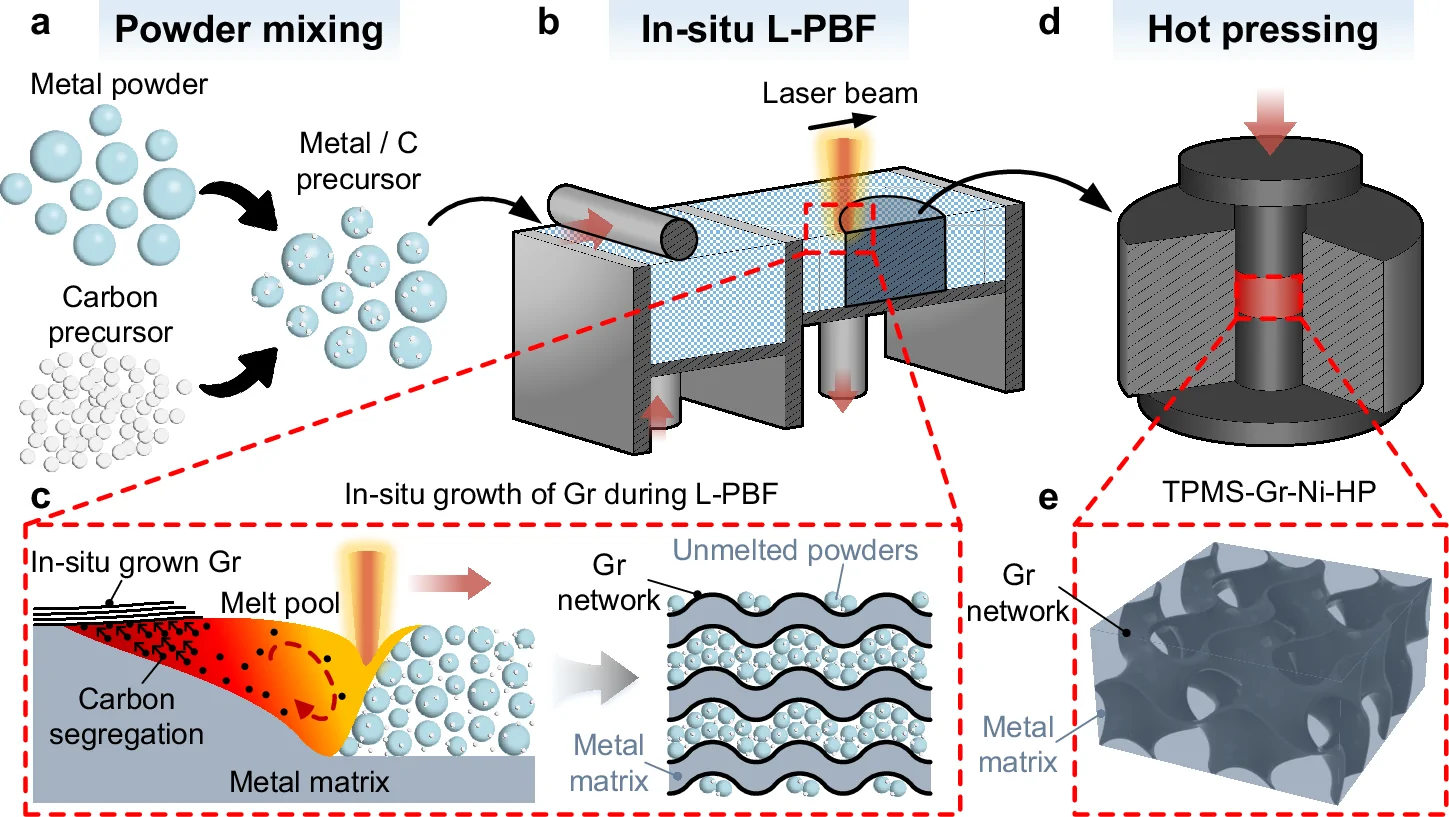
Schematic illustration of in-situ L-PBF AM of 3D graphene network reinforced MMCs processes. **a** The metal powders, e.g. Ni powders in this study, were mixed with solid carbon precursor powders, e.g., PMMA via ball milling to form the hybrid powder preform. **b** Load the hybrid powder to a L-PBF printer for layer-by-layer selective laser melting, enabling the fabrication of bulk or architected lattice structures. **c** Mechanism of in-situ graphene (Gr) growth during laser powder bed fusion (L-PBF) processing. Driven by the rapid non-equilibrium solidification of the laser-induced melt pool, dissolved carbon atoms segregated toward the solidification front and melt pool surface, precipitating into few-layer graphene upon cooling. For triply periodic minimal surface (TPMS) lattices, graphene in-situ grew on both inner and outer surfaces of printed struts to form a continuous interconnected network, with unmelted precursor powders retained in lattice pores. **d** The as-printed metal lattice with the as-grown graphene networks was subjected to fast hot-pressing (HP) device, which fully densifies the metal matrix and consolidates residual unmelted powders while preserving the integrity of the three-dimensional graphene network. **e** The schematic of fully densified composite with a three-dimensional interpenetrating Gr network as the reinforcement.

In this study, we propose a transformative strategy that integrates the laser-induced graphene synthesis directly into the L-PBF process to achieve architectured 3D nano-reinforcement networks, as illustrated in Fig. 1. A Ni matrix with a triply periodic minimal surface (TPMS) gyroid scaffold was printed via L-PBF with Ni/Polymethyl methacrylate (PMMA) pre-mixed powders. Laser heating triggers the pyrolysis and decomposition of PMMA simultaneously melts the Ni powders. The released carbon atoms can diffuse into the molten Ni, and during the Ni melt pool solidifies, the dissolved carbon can precipitate to the Ni clad surface to form graphene layers (Supplementary Fig. 1). The architectured Ni TPMS scaffold with conformal graphene layer wrapping was directly obtained in L-PBF and subsequently densified via hot pressing to consolidate the 3D graphene network and achieve a fully dense bulk composite. Our method bypasses the inherent technical constraints of multi-material printing by using the melt pool temperature profile control in the powder bed to drive the decomposition of carbon precursors and the subsequent carbon precipitation and growth of graphene on the metal matrix being printed simultaneously. As a geometrically desired metal skeleton is built layer-by-layer, a high-quality 3D graphene network is in-situ synthesized on its surface, enabling spatial configuration control of carbon nanomaterial reinforcement within metal. The proposed Ni-Gr composite exhibits pronounced synergistic enhancement in strength and toughness. This in-situ synthesis approach for AM of architectured Gr-MMCs represents a significant advancement for harnessing the superb intrinsic properties of low-dimensional carbon nanomaterials in the bulk MMCs.

## Results

### Process-structure relationship in the in-situ synthesis of Gr during L-PBF

**Fig. 2:**
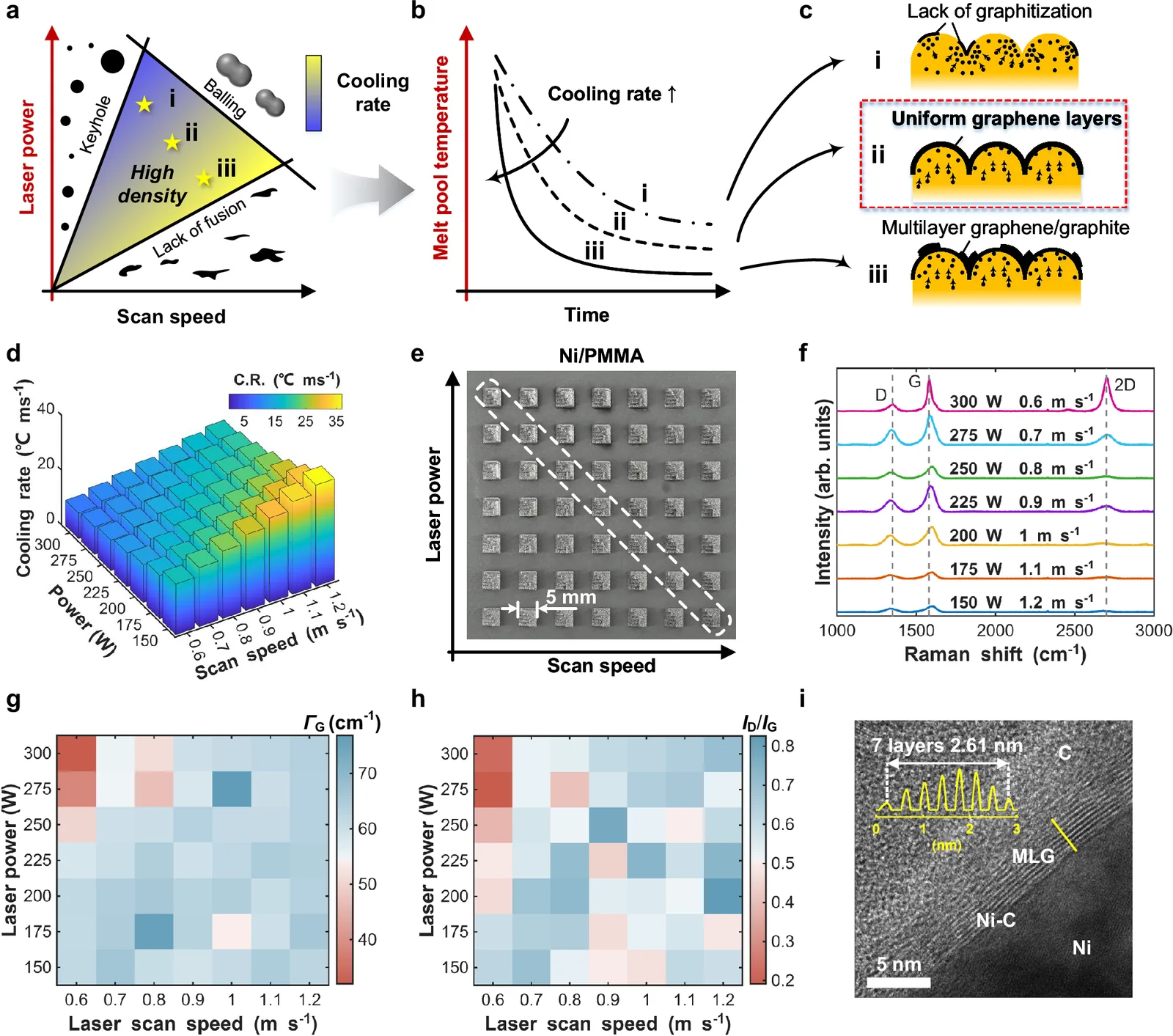
Characterizations of in-situ grown graphene in L-PBF. **a** The schematic of typical process window of L-PBF AM for full-density metal part printing. “i”, “ii”, “iii” stars represent three full-density printing settings but with different laser power *P*_*l*_ and laser scan speed *U*_*l*_. The color map represents the melt pool cooling rate. **b** The schematic to show melt pool cooling curves of “i”, “ii”, “iii” stars in (**a**). **c** Different cooling rates (C.R.) in (**b**) lead to different carbon precipitation flux at graphene synthesis temperature on the solidified Ni clad surface. Then “i”, “ii”, “iii” laser settings could result in lack of graphitization, uniform graphene layers growth, and MLG or graphite formation on the surface of the printed part. **d** Melt pool cooling rate varies with *P*_*l*_ and *U*_*l*_. **e** A photo of printed test coupons with different L-PBF laser settings corresponding to (**d**), using uniformly mixed Ni and Polymethyl methacrylate (PMMA) powder. **f** The corresponding Raman spectra of the printed Ni-Gr cube surfaces in the white dashed square in (**e**). **g**, **h** The Raman G peak’s full width at half maximum (FWHM, *Γ*_G_), and D to G peak intensity ratio (*I*_D_/*I*_G_) distribution corresponding to the 49 printed samples in (**e**). **i** High resolution transmission electron microscope (HRTEM) image of as-printed Ni-Gr cross section. Scale bar, 5 nm. Source data are provided as a Source Data file.

In graphene synthesis, whether by conventional CVD^44^ or laser CVD^45,46^, the cooling rate is a critical parameter as it governs the temperature-dependent solubility and surface precipitation flux of carbon atoms, thereby controlling the nucleation and the crystallographic quality of the resulting *sp*^2^ carbon lattice^47–49^. In L-PBF, the laser parameters can tune the melt pool temperature profile, thus controlling the kinetics of carbon segregation and precipitation. Figure 2a illustrates the typical process window for fabricating fully dense metal parts via L-PBF^50^. It is important to note that within the full density printing region, different combinations of laser power (*P*_*l*_) and scan speed (*U*_*l*_), labeled “i”, “ii”, and “iii”, result in significantly varying thermal histories. The corresponding cooling curves for these three parameter sets are shown in Fig. 2b. The cooling rate determines the Ni clad surface temperature and carbon solubility in Ni, therefore controls the carbon precipitation flux at the solidified surface (Supplementary Fig. 2). During rapid cooling of the melt pool, carbon solubility, expressed as $S={S}_{0}\exp (\frac{H}{{kT}})$ (in atoms mm^−3^), can precipitously drop below the initial carbon concentration in the mixed powder feedstock, where ${S}_{0}=5.33\times {10}^{25}$ atoms mm^−3^ is an entropic pre-factor, H=−0.42 eV is the heat of precipitation and *k* is Boltzmann’s constant^51^ (Supplementary Note 1 and Supplementary Fig. 2). Driven by strong chemical potential and temperature gradients, carbon atoms precipitate onto the solidifying Ni surface. Such different cooling conditions may lead to distinct graphitization outcomes, as illustrated in Fig. 2c. An excessively high cooling rate (process “iii”) results in insufficient carbon segregation, yielding a lack of graphitization on the Ni surface. Conversely, a cooling rate that is too low (process “i”) promotes excessive carbon precipitation, forming thick, multilayer graphene (MLG) or graphite. An optimal intermediate cooling rate (process “ii”) enables the precipitation flux necessary for uniform, high-quality graphene growth.

To experimentally map the laser process—graphene structure relationship, we printed a matrix of Ni coupon cubes across a range of *P*_*l*_ (150–300 W) and *U*_*l*_ (0.6–1.2 m s^−1^), as shown in Fig. 2e. The corresponding melt pool cooling rates at specific temperature *T*_*0*_ were estimated using a simplified model adapted from laser welding^52^, expressed as $C.R.\propto \frac{\lambda {U}_{l}}{{P}_{l}\,\eta {T}_{0}}$ (Fig. 2d), where λ is the thermal conductivity of metal matrix. Raman spectroscopy of the as-printed surfaces (Fig. 2f–h) confirms the modelled trend. More detailed Raman spectra and surface morphologies are presented in Supplementary Fig. 3. With increasing cooling rate, we observed a systematic decrease in both the Raman D to G peak intensity ratio (*I*_D_/*I*_G_) and the G peak full width at half maximum (FWHM, *Γ*_G_), indicating a reduction in defect density and improved crystallinity (Supplementary Note 2). The sample printed at 300 W and 0.6 m/s exhibited the most prominent 2D peak (*I*_2D_/*I*_G_ ≈ 1), signifying the local formation of high-quality, few-layer graphene (FLG). In addition, the peak separation between the D’ peak and the G peak in the Raman spectrum rules out the presence of graphene derivatives^53,54^ (Supplementary Note 3 and Supplementary Fig. 4). It is noted that the initial layers of graphene grown on the surface of the Ni matrix typically do not exhibit measurable features in Raman spectra^55^. Cross-sectional high-resolution transmission electron microscopy (HRTEM) image confirmed the presence of FLG growth on the Ni surface (Fig. 2i). Notably, no obvious Ni carbide phase^56^ was detected within the fabricated composites using X-ray photoelectron spectroscopy (XPS) and X-ray diffraction (XRD), as shown in Supplementary Fig. 5a-c. However, a slight lattice distortion of Ni is observed at the Gr-Ni interface, which is most likely induced by the infiltration of carbon atoms and provides direct evidence for the intimate Ni–Gr interface (Supplementary Fig. 5d-f, and Supplementary Note 4). Notably, the proposed in-situ L-PBF printing technique is also applicable to various metallic materials beyond Ni (Supplementary Fig. 6 and Supplementary Note 5).

**Fig. 3:**
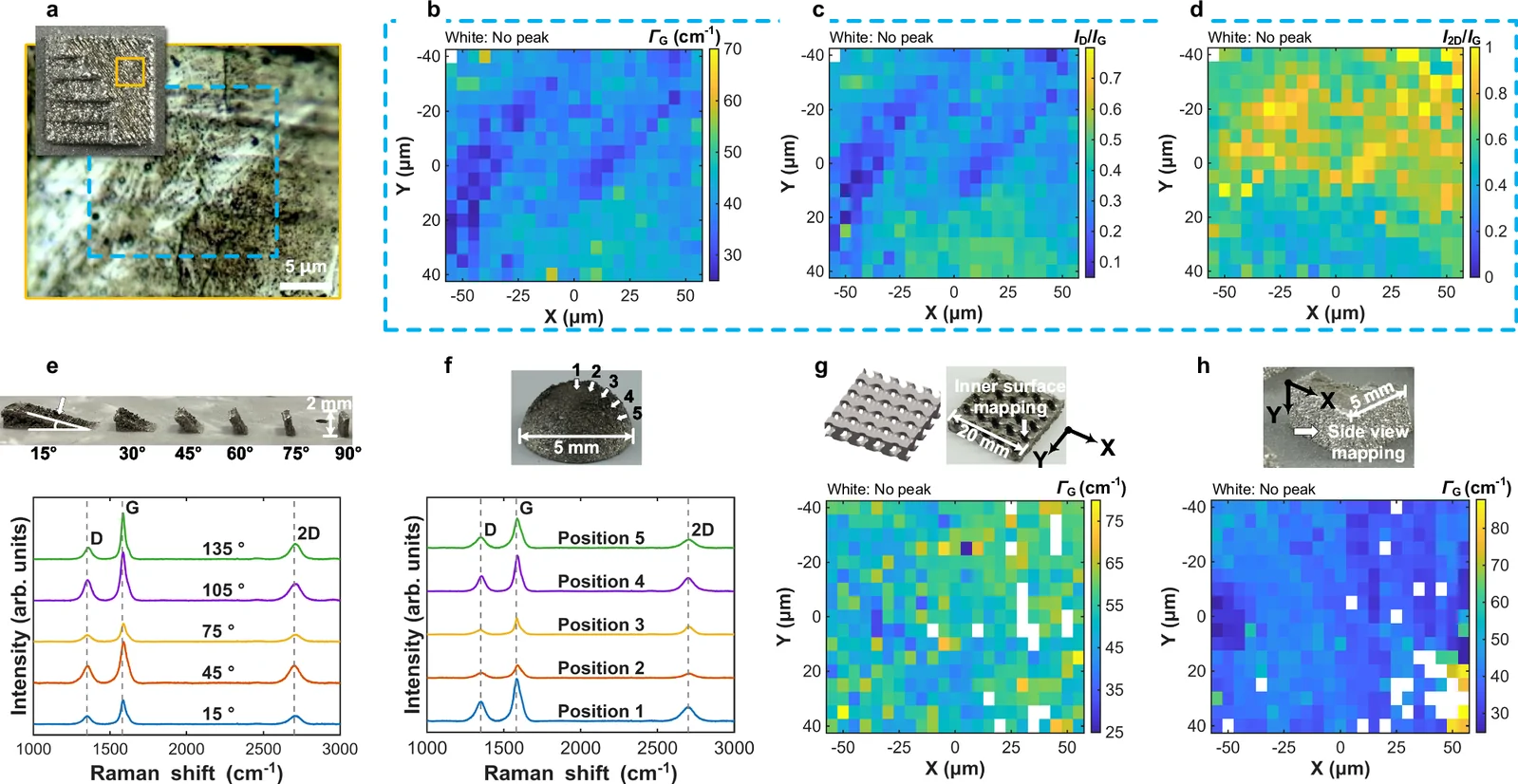
Coverage of as-grown graphene on L-PBF printed surfaces. **a** Optical microscope image of printed top surface with as-grown graphene coating. The inset shows the macroscopic morphology of the bulk sample. scale bar, 20 μm. **b**–**d** The Raman G peak’s full width at half maximum (FWHM, *Γ*_G_), D to G peak intensity ratio (*I*_D_/*I*_G_), and 2D to G peak intensity ratio (*I*_2D_/*I*_G_) maps of the dashed square region in (**a**), showing full coverage of graphene layers on the printed top surface with high quality crystallinity. **e** Representative Raman spectra of printed surfaces with different inclination angles. The upper panel displays the morphologies of printed samples with various angles. **f** Representative Raman spectra collected from the curved surface of a printed hemispherical sample. The upper panel marks five detection positions (1–5) distributed sequentially from the center to the edge of the hemisphere, and the lower panel shows the Raman spectra corresponding to positions 1–5, respectively. **g** *Γ*_G_ Raman mapping of the interior surface of a printed gyroid lattice structure. The upper panel shows the schematic of the gyroid structure and the targeted inner testing surface, and the mapping result confirms graphene coverage on the internal surface of the lattice. **h** *Γ*_G_ Raman mapping of a printed vertical sidewall. The upper panel shows photo of the sample with the testing surface marked, and the result verifies continuous graphene growth with high crystallinity on the vertical side surface. Source data are provided as a Source Data file.

To evaluate the coverage and conformality of the in-situ grown graphene on 3D-printed Ni surface, we performed detailed Raman spectroscopy mapping on samples fabricated using optimized parameters (300 W, 0.6 m s^−1^). The top surface of a printed bulk sample exhibits a graphene coverage over 98% (Fig. 3a–d and Supplementary Fig. 7a-c). The Raman *I*_D_/*I*_G_ values (all <0.56) and *I*_2D_/*I*_G_ ratios, predominantly ranging from 0.3 to 1.34, indicate the formation of FLG with uniformly high quality^6^. Meanwhile, abundant SEM images validate the continuous graphene growth (Supplementary Fig. 8). Notably, such conformal growth of high-quality graphene is not restricted to flat horizontal surfaces. High-quality FLG was successfully synthesized on non-planar geometries, including inclined surfaces (15-135°, Fig. 3e) and curved hemispheres (Fig. 3f), showcasing the flexibility of the in-situ graphene synthesis and its compatibility with the L-PBF process. Furthermore, growth can reach complex internal architectures. Raman mapping of the interior surface of a printed gyroid structure (Fig. 3g and Supplementary Fig. 9) and vertical sidewalls (Fig. 3h and Supplementary Fig. 7e-l) revealed high graphene coverage rates of 95% and 94%, respectively, confirming the formation of a continuous FLG 3D wrapping on the metal scaffold during printing. The few areas lacking Raman signal are attributed to topological undulations of the gyroid structure and defocusing of the laser in Raman spectroscopy (Supplementary Fig. 9g and h). Furthermore, morphological observations verify that the in-situ formed FLG achieves full, conformal coverage over the entire 3D printed Gyroid skeleton (Supplementary Fig. 10). The proposed in-situ L-PBF process thus enables active control over the FLG 3D spatial configuration. Crucially, during subsequent hot-pressing densification, the unmolten powders trapped within the scaffold’s pores serve as internal support and filler material. This mechanism minimizes bulk shrinkage and macroscopic deformation while enabling full densification, thereby preserving the pre-designed 3D architecture of the FLG coating within the final metal matrix.

### Microstructure of as-fabricated Ni-Gr composites

Following high-quality, conformal graphene growth during L-PBF, we consolidated the architectured scaffolds via hot pressing to fabricate fully densified bulk Ni-Gr composites. The resulting microstructures were characterized by Electron Backscatter Diffraction (EBSD) analysis, which revealed profound differences introduced by the in-situ grown graphene in the Ni matrix. The continuity of the grown 3D graphene architecture remains preserved within in the densified Ni matrix (Supplementary Note 6 and Supplementary Figs. 11–12).

We compared four distinct conditions: the proposed architecture after hot pressing (Ni/PMMA-Lattice-HP), a control without graphene (Ni-Lattice-HP), and two as-printed references (Ni-Bulk and Ni/PMMA-Bulk with FLG growth occurred on each clad surface). In the Ni/PMMA-Bulk specimen, Raman spectroscopy line scanning on the cross-sectional surface elucidates the dynamic nature of graphene incorporation during layer-by-layer Ni printing (Supplementary Fig. 13a and b). The FLG layers synthesized on the surface of one clad were torn, fragmented, and redistributed within the melt pool during the subsequent laser melting cycle, since the melt pool depth is usually larger than the layer thickness for full density printing^57^. This process results in graphene flakes of varying thicknesses being primarily encapsulated at solidification boundaries, creating a characteristic periodic graphitization pattern in the cross-section (Supplementary Fig. 13c and d). This fragmented yet patterned distribution directly produces a heterogeneous grain structure in as-printed samples, as clearly evidenced by the EBSD analysis (Supplementary Fig. 14). Pure Ni printed specimen (Ni-Bulk) exhibits a strong crystallographic texture alongside coarse, columnar grains dominating the microstructure, which is a typical consequence of directional solidification under high temperature gradients. The Ni/PMMA-Bulk composite develops a distinct hierarchical grain structure. The graphene fragments at melt pool boundaries effectively interrupt directional columnar growth, supply nucleation sites, promote the formation of finer, equiaxed grains in these regions, and result in a significantly randomized crystallographic texture (Supplementary Fig. 14d-f).

**Fig. 4:**
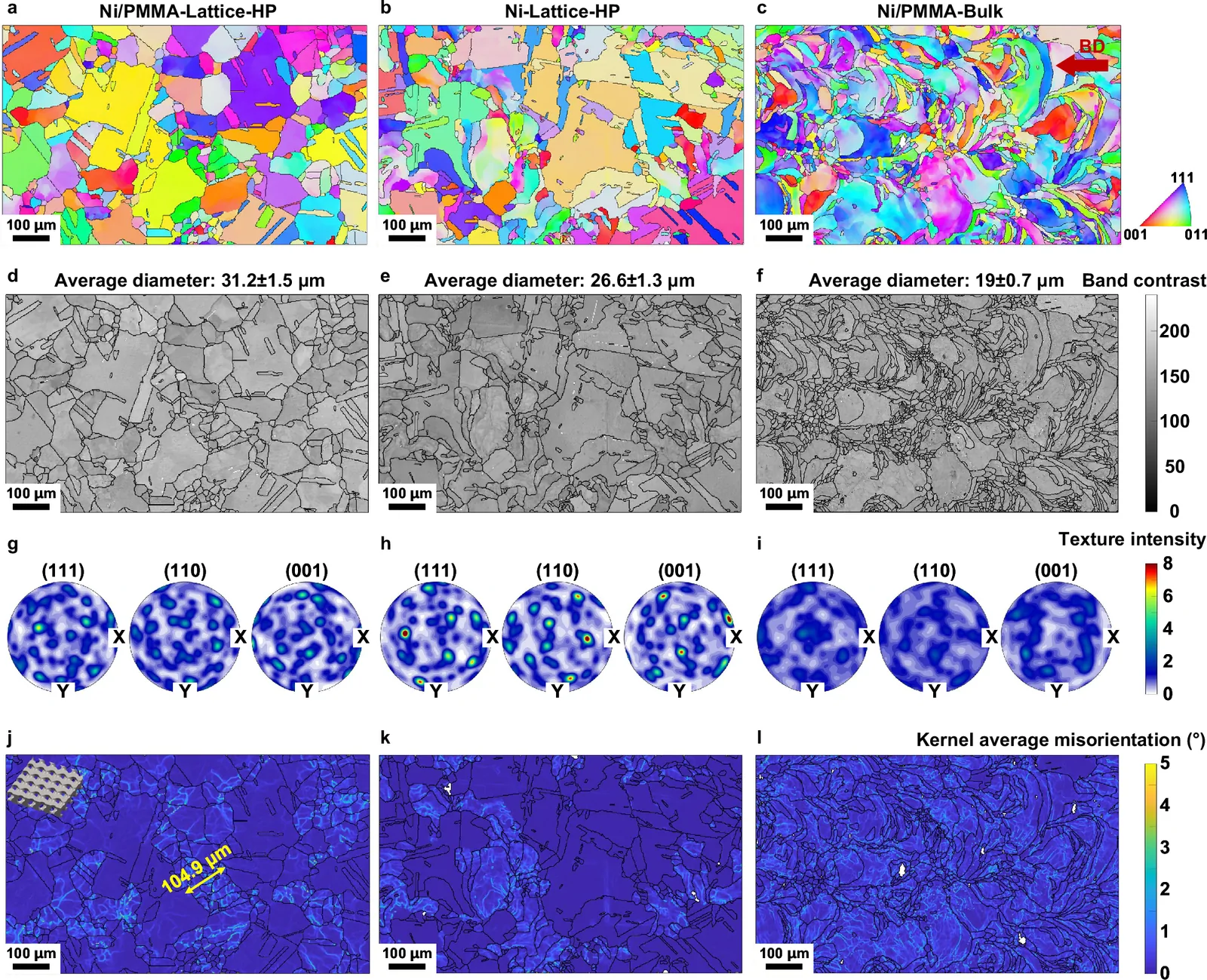
Microstructure characterizations of as-fabricated Ni and Ni-Gr composites. **a**–**c** EBSD inverse pole figure (IPF) maps of the proposed Ni MMC with in-situ grown graphene network reinforcement (Ni/PMMA-Lattice-HP), control group densified L-PBF printed pure Ni lattice via hot pressing (Ni-Lattice-HP), and solid L-PBF printed bulk part with in-situ graphene layers growth during layer-by-layer building (Ni/PMMA-Bulk). The red arrow presents the building direction (BD) of L-PBF process. **d**–**f** Grain boundary maps of Ni/PMMA-Lattice-HP, Ni-Lattice-HP, and Ni/PMMA-Bulk, respectively. Hot pressing process slightly coarsened the grain size of the printed part. The grain size data are presented as mean  ±  standard error. scale bar, 100 μm (**a**–**f**). **g**–**i** Pole figure (PF) maps of Ni/PMMA-Lattice-HP, Ni-Lattice-HP, and Ni/PMMA-Bulk, respectively. Notably, for Ni-Lattice-HP without graphene synthesis, a clear texture pattern can be observed. **j**–**l** The kernel average misorientation maps of Ni/PMMA-Lattice-HP, Ni-Lattice-HP, and Ni/PMMA-Bulk, respectively. The geometrically necessary dislocations (GNDs) density is high in the L-PBF-printed regions due to fast heating and cooling. For the Ni/PMMA-Lattice-HP, the KAM map shows a periodic pattern as the printed gyroid Ni-Gr scaffold, scale bar, 100 μm (**j**–**l**).

This texture-homogenizing effect due to in-situ graphene growth persists after the hot-pressing densification. The Ni/PMMA-Lattice-HP sample lacks the strong texture characteristic (texture intensity: 4.66) and exhibits an average grain size of 31.2 μm, compared to the Ni-Lattice-HP sample, which shows a higher texture intensity (7.28) and an average grain size of 26.6 μm (Fig. 4). Both the Ni/PMMA-Lattice-HP and the Ni-Lattice-HP samples show moderate grain coarsening compared to Ni/PMMA-Bulk because of hot pressing. What should be noted is, the kernel average misorientation (KAM) maps provide definitive microstructural evidence for the preservation of the designed 3D graphene architecture. As compared in Fig. 4j–l, the Ni/PMMA-Lattice-HP sample displays a distinct, periodic pattern of high geometrically necessary dislocation (GND) density that mainly replicates the designed gyroid scaffold. This patterned dislocation density originates from the strain incompatibility and impeded dislocation motion at the interfaces between the printed Ni framework and the consolidated powder regions, where the FLG grows (Supplementary Fig. 11). Furthermore, FEM results verify that and the as-grown FLG are not fully disrupted after hot pressing, therefore, the continuity of the 3D graphene architecture remains preserved (Supplementary Fig. 12).

### Mechanical performance and deformation mechanisms

Prior to mechanical testing, thermogravimetric analysis (TGA) was applied to the pre-mixed Ni/PMMA powders and confirmed about 97.6% decomposition of the PMMA precursor at temperatures over 500 °C, with only 2.4% residual carbon at 1100 °C (Supplementary Fig. 15a). Further, computed tomography (CT) scans revealed the density of the as-fabricated Ni/PMMA-Lattice-HP, Ni-Lattice-HP, Ni/PMMA-Bulk samples over 99.9%, 99.9%, and 99.5% respectively. All specimens were free of macro-defects, confirming that the proposed in-situ L-PBF process does not compromise structural integrity (Supplementary Fig. 15b-d). This is partly attributed to the high in-plane thermal conductivity of in-situ grown graphene, which enables efficient homogenization of the melt pool’s and solidifying cladding’s temperature field^58,59^ (Supplementary Fig. 16 and Supplementary Note 7).

**Fig. 5:**
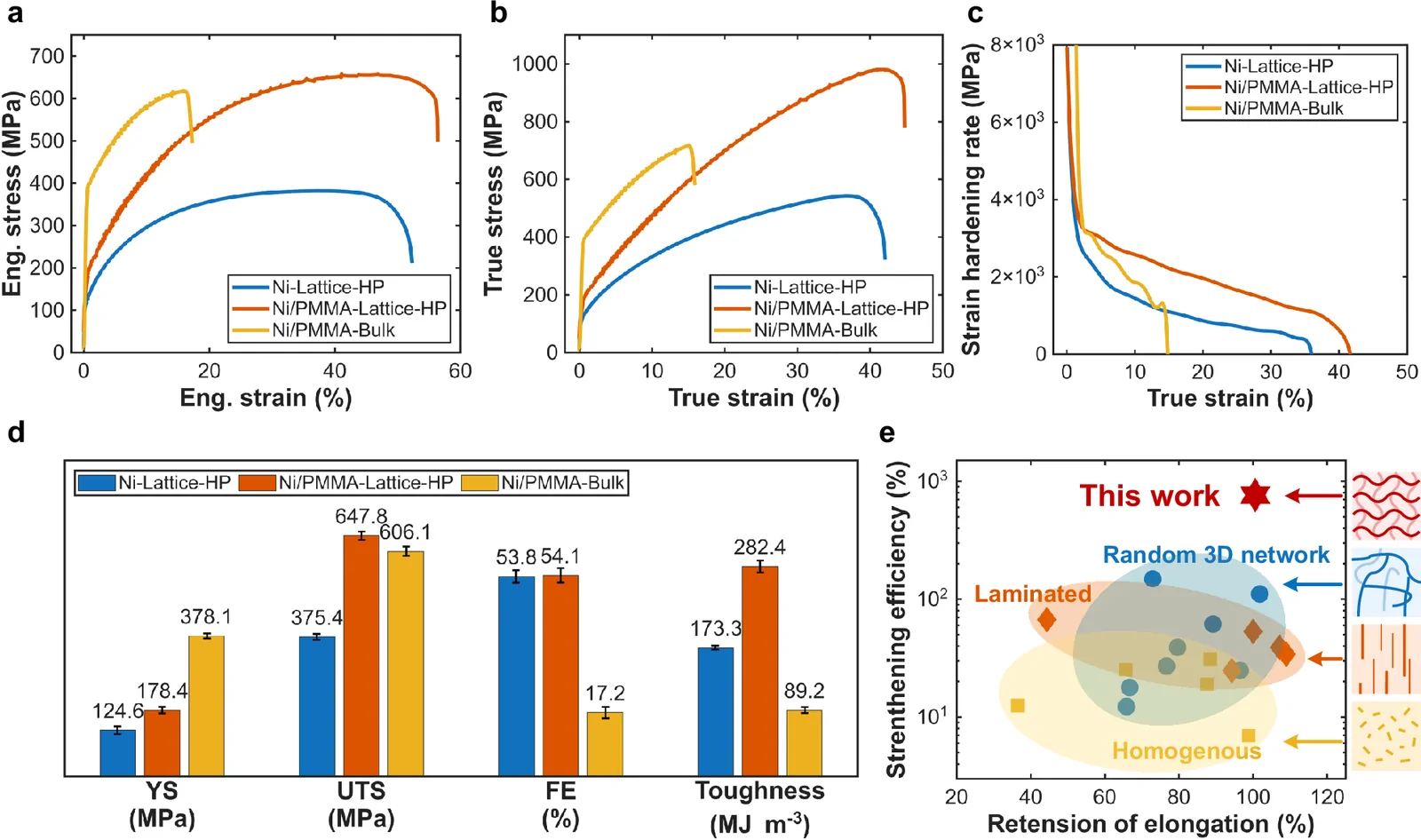
Uniaxial tensile properties of as-fabricated Ni and Ni-Gr composites. **a**, **b** Engineering and true tensile stress-strain curves of Ni/PMMA-Lattice-HP, Ni-Lattice-HP, and Ni/PMMA-Bulk, respectively. **c** Strain hardening rate plotted against true strain, derived from the corresponding stress-strain curves, indicating the Ni/PMMA-Lattice-HP sample exhibits a higher work hardening rate throughout the entire deformation process, benefiting from the three-dimensional interlocked graphene network. **d** Comparison of key mechanical properties of Ni/PMMA-Lattice-HP, Ni-Lattice-HP, and Ni/PMMA-Bulk, showing that the as-prepared Ni/PMMA-Lattice-HP composite had an outstanding combination of strength and toughness. Error bars represent the standard deviation of five independent parallel tensile tests. **e** Benchmark plot of fracture elongation retention versus graphene strengthening efficiency, comparing the in-situ architectured graphene network in this work with typical graphene reinforcement configurations reported in the literature, including homogeneous dispersion, laminated structure, and random 3D network. The result demonstrates that the in-situ constructed 3D interpenetrating graphene network achieves superior strengthening efficiency while maintaining high ductility retention, outperforming conventional architectures. Detailed references and data for the literature benchmarks are provided in Supplementary Table 1 in the SI. Source data are provided as a Source Data file.

Figure 5 shows the unidirectional tensile testing results revealing the profound impact of 3D graphene architecture on the composite’s mechanical performance. The Ni/PMMA-Lattice-HP composite with the 3D graphene network demonstrates an exceptional strength-ductility synergy, exhibiting an ultimate tensile strength (UTS) of 647.8 MPa and fracture elongation (FE) of 54.1%. This represents a 72.3% increase in UTS while maintaining ductility compared to the graphene-free control (Ni-Lattice-HP: UTS = 375.4 MPa, FE = 53.8%), and simultaneous enhancement in UTS and FE compared to the specimen with in-situ graphene growth but no configuration control, namely Ni/PMMA-Bulk (UTS = 606.1 MPa, FE = 17.2%). Consequently, the toughness of Ni/PMMA-Lattice-HP reaches 282.4 MJ m^−3^, a 63.2% enhancement over pure Ni. Figure 5c compares strain hardening behaviors: the Ni-Lattice-HP sample exhibits a characteristically slow decay in hardening rate, typical of pure metals where dislocation accumulation proceeds uniformly. In contrast, Ni/PMMA-Bulk with randomly dispersed graphene fragments shows significantly higher initial strength (YS = 378.1 MPa) but suffers rapid hardening capacity exhaustion, leading to premature necking and limited ductility (FE = 17.2%). Remarkably, Ni/PMMA-Lattice-HP maintains superior strain hardening rates throughout deformation, indicating sustained dislocation storage and multiplication enabled by the 3D Ni-Gr interpenetrating configuration. This continuous hardening promotes extensive uniform elongation while effectively suppressing localized failure. It should be emphasized that the strength improvement in composite can be attributed to multiple strengthening mechanisms quantitatively, including grain refinement, dislocation strengthening, and load transfer, where the high load transfer efficiency originating from the 3D graphene network dominants, as detailed and calculated in Supplementary Note 8, and compared in Supplementary Fig. 17.

Quantitative analysis of the graphene reinforcement’s strengthening efficiency, defined as $({\sigma }_{{UTS}-{composite}}-{\sigma }_{{UTS}-{matrix}})/f{\sigma }_{{UTS}-{matrix}}$, versus ductility retention shows that the 3D graphene architecture outperforms both homogeneous dispersions and laminated structures reported in literature (Fig. 5e and Supplementary Table 1). This superior performance originates from optimal load transfer and effective dislocation management enabled by the continuous, spatially configured in-situ grown high-quality graphene network. Furthermore, compression tests along three orthogonal directions of Ni/PMMA-Bulk specimens were conducted, with nearly identical yield strengths across all orientations (Supplementary Fig. 18). This validates that the in-situ grown graphene network effectively homogenizes crystallographic texture, eliminating the anisotropic mechanical response typically observed in L-PBF-printed metals^60,61^. The combination of isotropic behavior with enhanced strength and toughness demonstrates the unique advantages of the architectured 3D graphene reinforcement strategy.

**Fig. 6:**
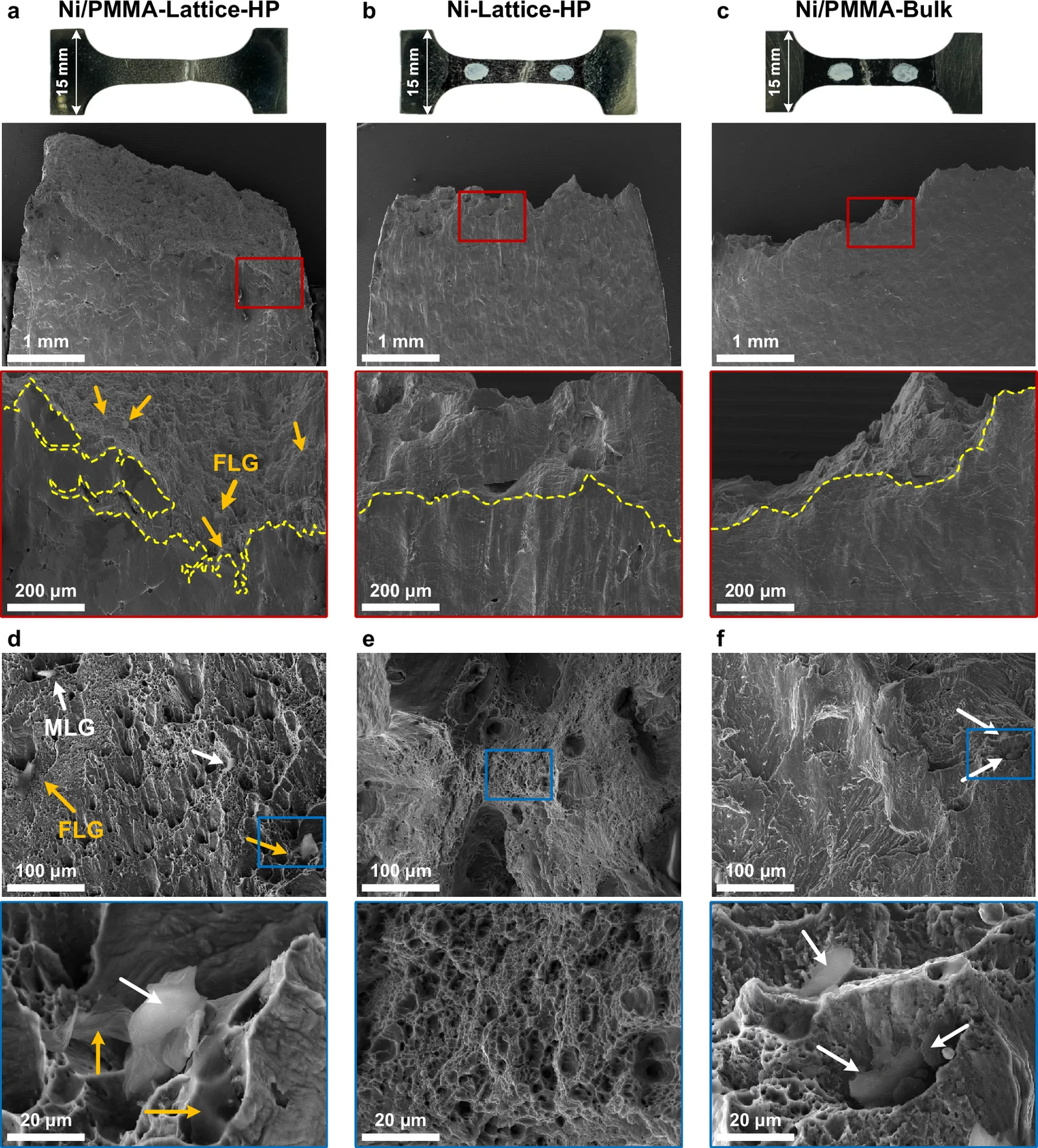
Fracture morphologies of as-fabricated Ni and Ni-Gr composites. Optical photographs and SEM images of tensile testing coupons and fracture surfaces of **a**, **d** Ni/PMMA-Lattice-HP composite, **b**, **e** Ni-Lattice-HP, and **c**, **f** Ni/PMMA-Bulk composite. Macroscopically, the Ni/PMMA-Lattice-HP composite exhibits extensive crack branching with multiple secondary and tertiary cracks deviating from the primary path, highlighted by yellow-dashed lines in (**a**, **d**), demonstrating a tortuous crack propagation mode that substantially enhances energy dissipation. Microscopically, the fracture surface shows a hierarchical dimple structure featuring small, elongated dimples interspersed among larger ones, suggesting heterogeneous plastic flow induced by the 3D graphene network. Crucially, abundant pulled-out and bent few-layer graphene (FLG) fragments are observed along the crack paths (indicated by orange arrows). The graphene-free control (Ni-Lattice-HP) exhibits classic ductile fracture characteristics in (**b**, **e**), characterized by pronounced macroscopic necking and a high density of uniformly distributed, conventional dimples across the fracture surface, typical of uniform plastic deformation in pure metals. The Ni/PMMA-Bulk specimen with a fragmented graphene distribution displays an intermediate but ultimately inferior fracture morphology in (**c**, **f**). Its premature brittle failure is evidenced by limited dimple formation on a flatter fracture surface containing only isolated, thicker multilayer graphene (MLG) flakes (indicated by white arrows). Scale bar, 1 mm for the top panel, 200 μm for the bottom panel (**a**–**c**), 100 μm for the top panel, 20 μm for the bottom panel (**d**–**f**).

Further analysis examined the tensile fracture morphologies of three specimens. The graphene-free Ni-Lattice-HP control exhibits classic ductile fracture characteristics, with pronounced necking and uniformly distributed dimples across its fracture surface (Fig. 6b and e). Conversely, the Ni/PMMA-Lattice-HP composite exhibits extensive crack branching with multiple secondary and tertiary cracks deviating from the primary path (Fig. 6a). This tortuous crack propagation markedly extends the total fracture pathway, substantially enhancing energy dissipation through continuous deflection and branching mechanisms^15^. Microscopically, the fracture surface shows a hierarchical dimple structure featuring small, elongated dimples interspersed among larger ones (Fig. 6d), suggesting heterogeneous plastic flow induced by the 3D graphene network. Crucially, abundant pulled-out and bent FLG fragments are observed along crack paths (orange arrows in Fig. 6d), providing evidence of efficient load transfer and pull-out mechanisms. The Ni/PMMA-Bulk specimen with fragmented graphene distribution exhibits an intermediate but ultimately inferior morphology: while possessing higher strength, its brittle failure was evidenced by limited dimple formation on the fractured surface containing only isolated, thicker MLG flakes (white arrows in Fig. 6f, Raman data in Supplementary Fig. 19), alongside significantly reduced elongation.

**Fig. 7:**
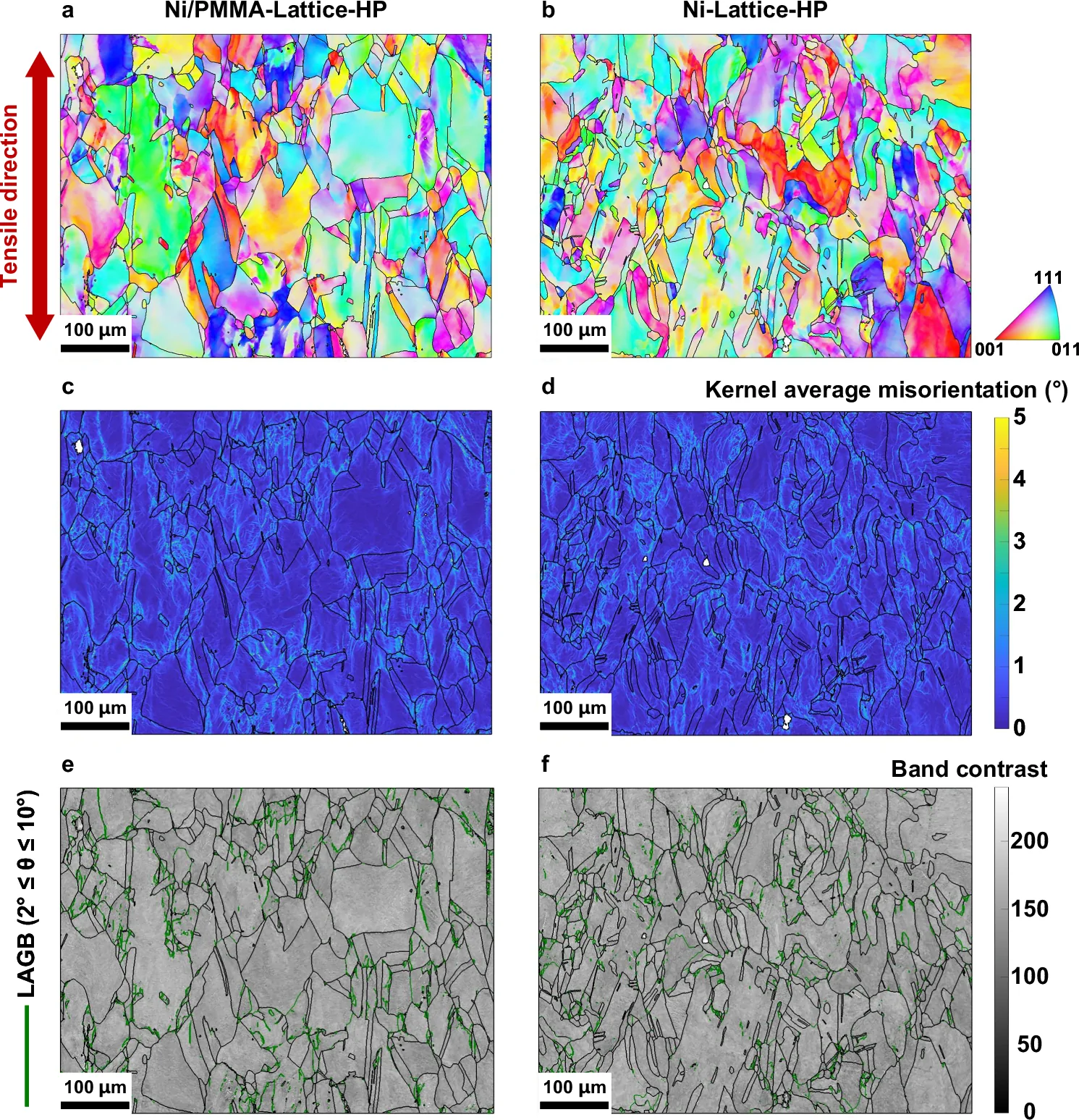
Microstructure characterizations of the fractured samples. **a**, **b** EBSD IPF map of the fractured Ni/PMMA-Lattice-HP and Ni-Lattice-HP sample respectively. **c**, **d** KAM map of the fractured Ni/PMMA-Lattice-HP and Ni-Lattice-HP sample respectively. **e**, **f** EBSD grain boundary with highlighted low angle (2° – 10°) grain boundary map of the fractured Ni/PMMA-Lattice-HP and Ni-Lattice-HP sample respectively. Notably, the grains were distorted and elongated along the tensile direction. The pronounced spatial fluctuations of KAM values in (**c**) indicating a heterogeneous distribution of GNDs and a residual plastic deformation capacity in the Ni/PMMA-Lattice-HP composite. Scale bar, 100 μm (**a**–**f**).

Furthermore, the fractured samples also exhibit different microstructural evolutions. The Ni-Lattice-HP exhibits homogeneous dislocation distribution and recrystallized columnar grains aligned with the tensile direction, indicative of conventional uniform plastic deformation (Fig. 7b, d, and f). In contrast, the Ni/PMMA-Lattice-HP develops a heterogeneous microstructure with pronounced spatial fluctuations in GND density (Fig. 7a, c, and e). The KAM map reveals selective dislocation accumulation at interfaces between fine-grained regions and the 3D graphene network, while preserving large equiaxed grains (30–50 μm) with minimal dislocation density. This heterogeneous GND distribution provides direct evidence of the composite’s residual plastic deformation capacity.

The 3D architectured graphene network enables multi-scale strengthening through several synergistic mechanisms: efficient load transfer across continuous interfaces, hetero-deformation induced strengthening from mechanical incompatibility between reinforced and unreinforced regions, and enhanced dislocation storage capacity evidenced by low-angle grain boundary (LAGB) concentration along graphene-rich interfaces (Fig. 7e). This architectural design explains the sustained strain hardening observed in Fig. 5c, where the network simultaneously restricts dislocation motion at interfaces while preserving deformation-capable zones within coarse grains. The combination of these intrinsic toughening mechanisms with extrinsic toughening via crack deflection represents a comprehensive strategy to overcome the strength-ductility trade-off in architectured MMCs (Supplementary Note 9).

## Discussion

The proposed manufacturing strategy combines laser-powder melting with in-situ material synthesis, establishing a unique route to achieve heterostructure construction in L-PBF AM. The fundamental physical principle lies in the disparate time scales of heat and mass transport. In Ni, the characteristic time scale for melting and solidification (governed by thermal diffusivity, ${\alpha }_{T}\left({{{\rm{Ni}}}}\right)\approx 22.3$ mm^2^ s^−1^) is several orders of magnitude shorter than the time required for carbon diffusion (mass diffusivity, ${\alpha }_{c}\left({{{\rm{Ni}}}}\right)\approx 3.2\times {10}^{-5}$ mm^2^ s^-1^ at 1000 °C) over a comparable length scale^51^. This creates a crucial process window: following solidification, the newly formed clad remains at an elevated temperature, allowing carbon atoms from a supersaturated solid solution to continue segregating. Driven by the strong temperature gradient, carbon precipitates to the clad surface^45,62^—a condition directly analogous to the thermal and chemical environment for graphene synthesis via CVD on high carbon solubility metals like Ni^44^. We have demonstrated that by strategically controlling the thermal cycle of the melt pool, graphene with tunable quality can be directly synthesized on each solidified layer during L-PBF printing. This Ni-Gr system serves as a foundational model. The core principle that significant temperature gradients and varying cooling rates within the standard L-PBF process window can drive designed chemical reactions, elemental segregation, or precipitation—is broadly applicable. It provides a programmable tool for constructing cross-scale architectural heterogeneity during metal matrix AM.

Building on this unique heterostructure design enabled by in-situ graphene synthesis, the mechanical performance of the Ni-Gr composites is inherently dominated by the interaction between the 3D architectured graphene network and the Ni matrix, as manifested in their tensile fracture behaviors. Distinct tensile fracture morphologies demonstrate that the 3D architectured graphene network not only serves as a structural reinforcement but also activates and coordinates multiscale toughening mechanisms^63^. Macroscopically, the continuous and interconnected graphene architecture exerts pull-out and/or bridging effects on propagating cracks. When the composite is subjected to tensile loading, the graphene network resists crack extension by transferring stress across the crack surfaces, forcing cracks to deviate from their original straight path and undergo tortuous branching. This extrinsic toughening mechanism dramatically increases fracture energy through repeated crack deflection, as the crack must overcome additional resistance to propagate along a non-linear trajectory. Simultaneously, at the microscopic scale, intrinsic toughening operates through graphene’s efficient load transfer capability at the Ni-Gr interface. The strong interfacial bonding (Fig. 2i) ensures that external stress is effectively transmitted from the Ni matrix to the graphene network, triggering heterogeneous plastic deformation in the Ni matrix surrounding the graphene network. This deformation leads to hierarchical dimple structures with varying sizes, where smaller dimples are observed in regions adjacent to graphene sheets (due to localized plastic constraint) and larger dimples dominate the matrix regions (Fig. 6d). Such hierarchical dimples further contribute to energy dissipation during fracture by accommodating more plastic deformation than the uniform dimples observed in the fracture of Ni-Lattice-HP (Fig. 6e). Importantly, the synergistic coordination between extrinsic crack control and intrinsic energy dissipation is a distinguishing feature of the in-situ constructed Ni-Gr heterostructure.

Taken together, these mechanical performance advantages and underlying mechanism insights originate from a transformative manufacturing strategy that integrates L-PBF AM, laser induced graphene growth, and hot pressing to fabricate Ni-based MMCs with architectured in-situ grown 3D graphene networks. The principal findings are: (i) the melt pool temperature profile, especially its cooling rate in L-PBF printing can be a tuning knob for component element diffusion and segregation in the melt pool and the solidifying clad. For example, the melt pool cooling rate controls the carbon precipitation flux towards the printed Ni surface from a pre-mixed Ni/PMMA powder bed and further achieves direct synthesis of continuous graphene layers with outstanding quality onto the printed Ni matrix. (ii) such in-situ graphene growth onto the metal surface, simultaneously being printed harnesses the 3D precision construction capability of AM and enables fabrication of architectured graphene 3D networks in the metal matrix. Graphene growth during layer-by-layer laser-metal melting and solidification effectively eliminates the crystallographic texture and mechanical anisotropy typical of L-PBF-printed metals. Further, the 3D metal-graphene architecture activates coordinated multi-scale toughening—combining extrinsic crack deflection with intrinsic strengthening mechanisms—to overcome the characteristic strength-ductility trade-off, achieving a 72% strength enhancement without compromising ductility in the fabricated Ni-Gr composite. (iii) the proposed in-situ L-PBF AM pathway enables programmable control over low-dimensional nanomaterials spatial configuration as superior reinforcements providing their intrinsic reinforcing efficiency in the macroscopic MMCs. This methodology transforms AM from a shaping tool into a platform for creating material architectures, expanding the design space for high-performance MMCs.

## Methods

### Materials

The Ni powders were purchased from Guangzhou Shinengine AM Technology Co., Ltd, with a particle size range of 15−53 μm, and a median diameter (D50) of 33.96 μm. PMMA powders were acquired from Dongguan Xinmiao New Materials Co., Ltd, with an approximate particle size of 1 μm. The two powders were mixed thoroughly using a planetary ball mill (MSK-SFM−1-1L, Kejing) at a speed of 300 rpm for 6 h to ensure homogeneity.

### Ni matrix fabrication and in-situ graphene synthesis

As shown in Fig. 1, we proposed a spontaneous in-situ graphene growth process during L-PBF AM for preparing MMCs with specific spatial configurations. It consists of two primary steps. Initially, L-PBF AM was employed to melt the pre-mixed Ni/PMMA powders, constructing a Ni matrix scaffold with a TPMS structure. We utilized a Gyroid structure with a unit cell size of 0.5 × 0.5 × 0.5 mm, a porosity of 0.5, and a featured wall thickness of around 100 μm. It provides numerous growth sites for graphene due to its large specific surface area. The laser processing parameters include a power of 300 W, a scan speed of 0.6 m s^-1^, a hatch distance of 0.1 mm, and a layer rotation of 67°.

Similar to laser CVD^46^, the growth of graphene during the L-PBF process involves three steps: (i) The PMMA decomposes and releases carbon atoms, which can dissolve in the Ni melt pool; (ii) As the melt pool cools, the solubility of carbon decreases exponentially, driving carbon atoms to segregate in the melt pool and precipitate towards the solidifying clad surface; (iii) The carbon atoms diffuse on the surface, nucleate with *sp*^2^ hybridization and grow. Through this in-situ L-PBF process, we constructed the Ni lattice framework layer by layer while simultaneously growing uniform graphene on both the inner and outer Ni surfaces. The pores of the framework intentionally trapped the unmolten Ni/PMMA powders for further densification.

### Densification via hot-pressing

The as-printed sample was placed in a graphite mold and subjected to vacuum sintering using rapid hot-pressing (HP) equipment (FHP-828, HAATN, China), as shown in Fig. 1c. The temperature and pressure were increased from ambient conditions to 1100 °C and 60 MPa over a period of 13 min, maintained for 60 min, and then cooled within the furnace under a pressure of 5 MPa. The volume fraction of graphene (0.095 vol.%) in the sample can be estimated by considering the specific surface area (11.2 mm^−1^) of the printed gyroid structure, the thickness of graphene layers grown on the structure surface ( ~ 100 nm), and the overall shrinkage rate of sample after sintering ( ~ 85%). A sample without a graphene network was prepared using pure Ni powders and the same procedures. The two samples are named Ni/PMMA-Lattice-HP and Ni-Lattice-HP, respectively.

### Fabrication of Ni/PMMA-bulk composites

We used pre-mixed Ni/PMMA powders to print dense solid blocks, including small cubes (Fig. 2e) and hemispheres (Fig. 3f), for characterization of the graphene on their external surfaces, and dog-bone shape samples for tensile tests. The L-PBF process settings have been described in section ‘Ni matrix fabrication and in-situ graphene synthesis’. These samples were fabricated directly by printing without undergoing HP and were referred to as Ni/PMMA-Bulk.

### Microstructure characterization techniques

The graphene layers grown on the Ni surface were characterized using a confocal Raman microscope system (inVia-Qontor, Renishaw), having a 532 nm wavelength laser, 1800 gr mm^−1^ (single-point scanning) and 1200 gr mm^−1^ (mapping) grating. EBSD analysis was performed using a Hitachi SU5000 SEM equipped with an Oxford AZtecLive UltimMax 65 system and a Symmetry S3 detector. EBSD specimens were mechanically ground with SiC papers up to 2000 grit and then polished with 0.05 μm SiO_2_ suspension, followed by ion milling at a tilting angle of 20° and a power of 6 kW for 1 hour. The samples were scanned at a step size of 2 μm with an operation voltage of 20 kV. The Kikuchi patterns were captured using an image size of 156×128 and an exposure time of 2.05 ms, without a pixel binning. The standard Hough transform-based algorithm was employed for pattern indexing. The initial indexing rate exceeded 95%, after which only zero solution filling was applied to the raw data within the Aztec software. The processed data were analyzed and plotted using the MTEX toolbox^64^. KAM images were generated by calculating the average misorientation angle between any specific pixel and all its neighboring pixels that reside within the same grain and exhibit a misorientation angle of less than 5°. Some SEM images were captured using a TESCAN MIRA4 system. The as-grown graphene on the Ni/PMMA-Bulk sample surface was characterized using HRTEM (JEM-F200, JEOL). The sample preparation utilized a dual-beam focused ion beam system (Crossbeam 350, Zeiss). The decomposition behavior of PMMA during sintering was characterized via TGA (SDT 650, TA), where PMMA powders were heated from room temperature to 1100 °C at the maximum heating rate of the equipment ( ~ 97.61 °C min^−1^). CT was conducted to measure the density of composites (FF 35CT, YXLON). XPS and XRD on Ni/PMMA-Lattice-HP sample were conducted to analyze the presence of carbides, using the Thermo Scientific K-Alpha system and Empyrean 3.0, respectively.

### Mechanical characterizations

The Ni/PMMA-Lattice-HP and the Ni-Lattice-HP samples were shaped into a dog-bone shape using wire-electrode cutting, whereas the Ni/PMMA-Bulk samples were directly printed in the dog-bone shape. The total length and gauge length of the tensile specimen are 38 mm and 10 mm, respectively; the parallel section has a width of 5 mm and a thickness of approximately 2 mm; the gripping section width is 15 mm; and the transition section features a 6 mm fillet radius. Room temperature tensile tests were conducted using an Instron 68TM-50 machine with a preload of 10 MPa and a constant loading rate of 0.2 mm min^−1^. Strain data for all tensile experiments were collected in real-time using a video extensometer. Uniaxial compression tests were carried out on an Instron 34TM-30 universal testing system at a constant crosshead speed of 0.15 mm min^−1^, where engineering strain is derived from crosshead displacement measurements. The compression samples were cut into cylinders with a height of 5 mm and a radius of 2.5 mm.

## Supplementary information

Supplementary Information

Transparent Peer Review file

## Source data

Source Data

## Data Availability

Source data generated in this study are provided in the Source Data file. Source data are provided with this paper.
