# Supplementary material for: In-situ architecturing of 3D graphene networks in metals via laser additive manufacturing for strength-toughness synergy
Source: Nat Commun. 2026 Aug 21;17:10004. doi: 10.1038/s41467-026-77015-z (PMC13590628; doi:10.1038/s41467-026-77015-z)
Supplement: Supplementary file 1 [file 41467_2026_77015_MOESM1_ESM.pdf]

Supplementary information

In-situ Architecturing of 3D Graphene Networks in Metals via Laser Additive Manufacturing for Strength-Toughness Synergy

Zhongkun Liao[1, 2], Jingchi Zhang[1], Zichuan Fu[1], Liang Zhao[1], Xiaotong Luo[1], Fan Shi[2], Baisong Guo[3], Kaihao Zhang[1, 2*]

[1]Smart Manufacturing Thrust, The Hong Kong University of Science and Technology (Guangzhou), Nansha District, Guangzhou, 511453, China

[2]Mechanical and Aerospace Engineering, The Hong Kong University of Science and Technology, Sai Kung, Hong Kong SAR, China

[3]Institute of Advanced Wear & Corrosion Resistant and Functional Materials, Jinan University, Guangzhou, 510632, China

Email: kaihaozhang@hkust-gz.edu.cn

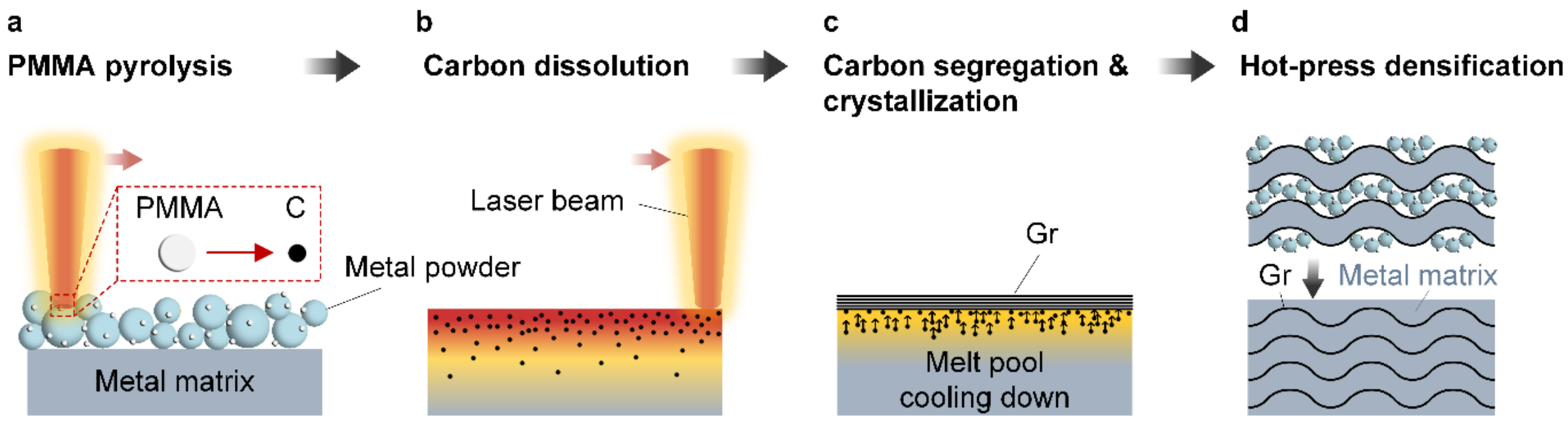

Supplementary Figure 1: In-situ graphene growth mechanism during L-PBF.

The in-situ L-PBF fabrication of Gr-MMCs involves four primary procedures: **a** Metal powders are melted to form a molten pool, and Polymethyl methacrylate (PMMA) particles are pyrolyzed into free carbon atoms; **b** Carbon atoms dissolve into the high-temperature molten pool; **c** During molten pool cooling, carbon atoms segregate to the surface and crystallize to form conformal graphene (Gr) layers; **d** Gr-MMCs with designed spatial architectures are densified via hot pressing.

Supplementary Note 1. Powder bed temperature profiles during layer-by-layer laser scanning in L-PBF

COMSOL finite element method (FEM) simulation was performed to analyze the powder bed temperature profile during the layer-by-layer L-PBF fabrication process (**Supplementary Fig. 2a**). Ni/PMMA composite powder was used as the experimental material, and pure Ni approximation was adopted in the model where thermophysical parameters are temperature dependent. The laser was modeled as a moving Gaussian heat source, with a power of 300 W and a scanning speed of 0.6 m s^{-1}. The heat transfer calculations consider interlayer heat conduction (activated layer by layer), as well as forced convection and thermal radiation at the top surface.

The simulation results qualitatively illustrate the thermal history of the powder bed. During the layer-by-layer deposition of Ni, the temperature field undergoes periodic increase and decrease (**Supplementary Fig. 2b**), which matches the experimental results (**Supplementary Note 7**). Correspondingly, the carbon solubility in Ni is governed by temperature as $S_p = S_0 \exp(H_p/kT)$, where S_0 is an entropic pre-factor, H_p is the heat of precipitation, k is Boltzmann's constant, and T is the temperature[1]. As a result, the solubility exhibits similar periodic variations (**Supplementary Fig. 2c**). The PMMA content in the mixed powder is 1 wt.%. In the case of complete pyrolysis of PMMA into carbon atoms, the atomic concentration of carbon in the Ni matrix is about 2×10^{27} atom m^{-3}. Once the carbon solubility drops below the threshold, carbon atoms start to segregate toward the printed surface.

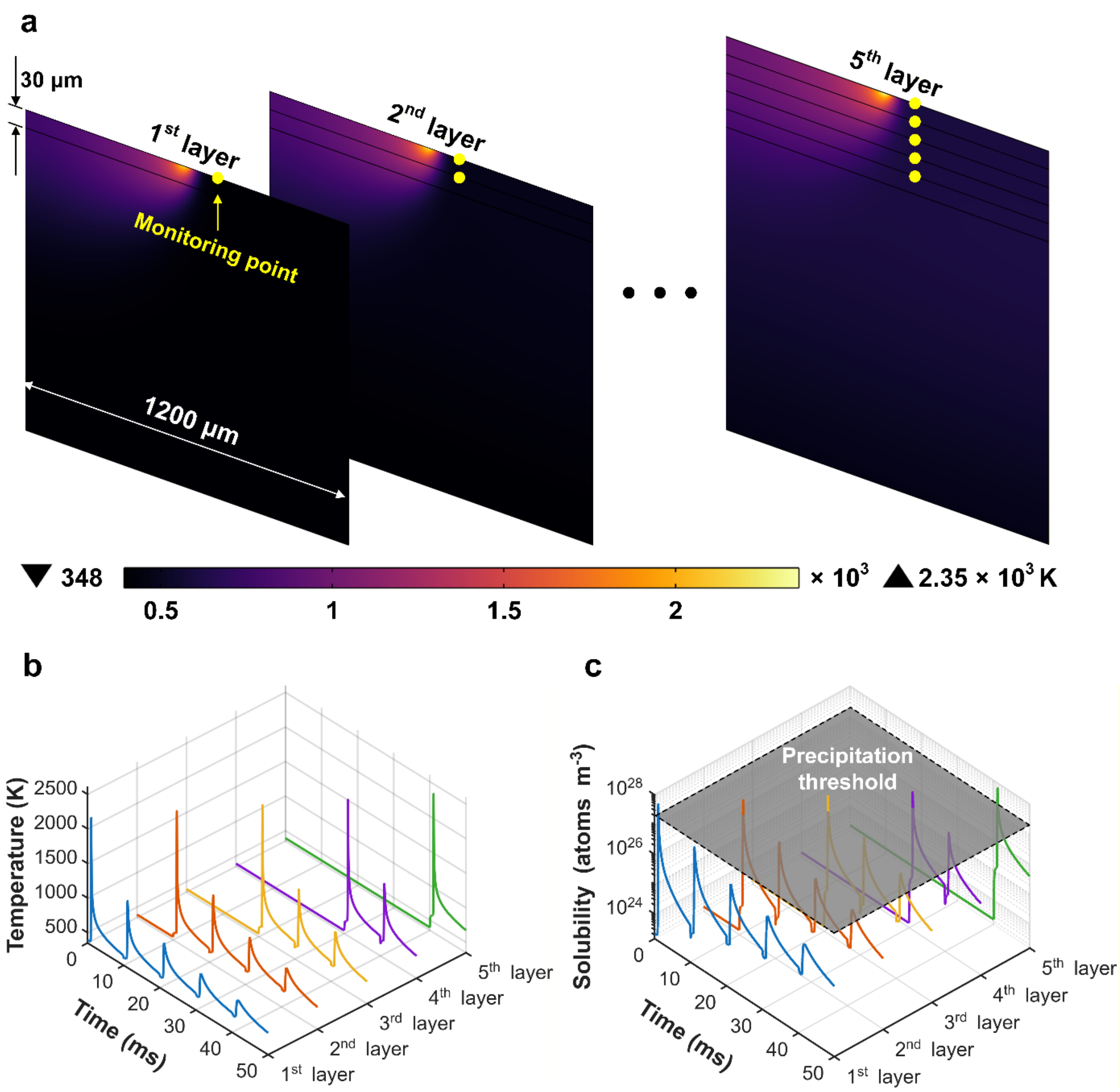

Supplementary Figure 2: Simulated powder bed temperature profiles during layer-by-layer laser scanning in L-PBF.

a COMSOL simulation result of temperature distribution during layer-by-layer sintering of Ni powder in L-PBF. **b**, **c** Temperature and carbon solid solubility ($S_p = S_0 \exp(H_p/kT)$ [1]) variations at each layer's monitoring point over time. Carbon atoms begin to precipitate when solubility falls below 2×10^{27} atoms m^{-3}. Source data are provided as a Source Data file.

Supplementary Note 2. Temperature driven Ni surface carbon precipitation and in-situ formation of FLG

The carbon segregation rate is affected by the molten pool cooling rate, which depends on the combination of laser power and scanning speed. Tuning laser parameters can modify carbon segregation behavior, thereby controlling the quality of graphene grown on the printed surface (**Supplementary Fig. 3**). At an excessively high cooling rate (laser power: 250 W, scanning speed: 1.2 m s^{-1}), Raman spectra indicate abundant defects in graphene ($I_D/I_G \approx 0.61$) and thick layers ($I_{2D}/I_G \approx 0.07$). Meanwhile, multi-layer graphene or graphite derived from excessive segregation can be observed via SEM (**Supplementary Fig. 3e**). In contrast, when carbon atoms segregate to the surface at a moderate rate, the crystallinity of formed graphene can be greatly improved ($I_D/I_G \approx 0.22$), and the product displays typical features of few-layer graphene ($I_{2D}/I_G \approx 1.09$, **Supplementary Fig. 3d**).

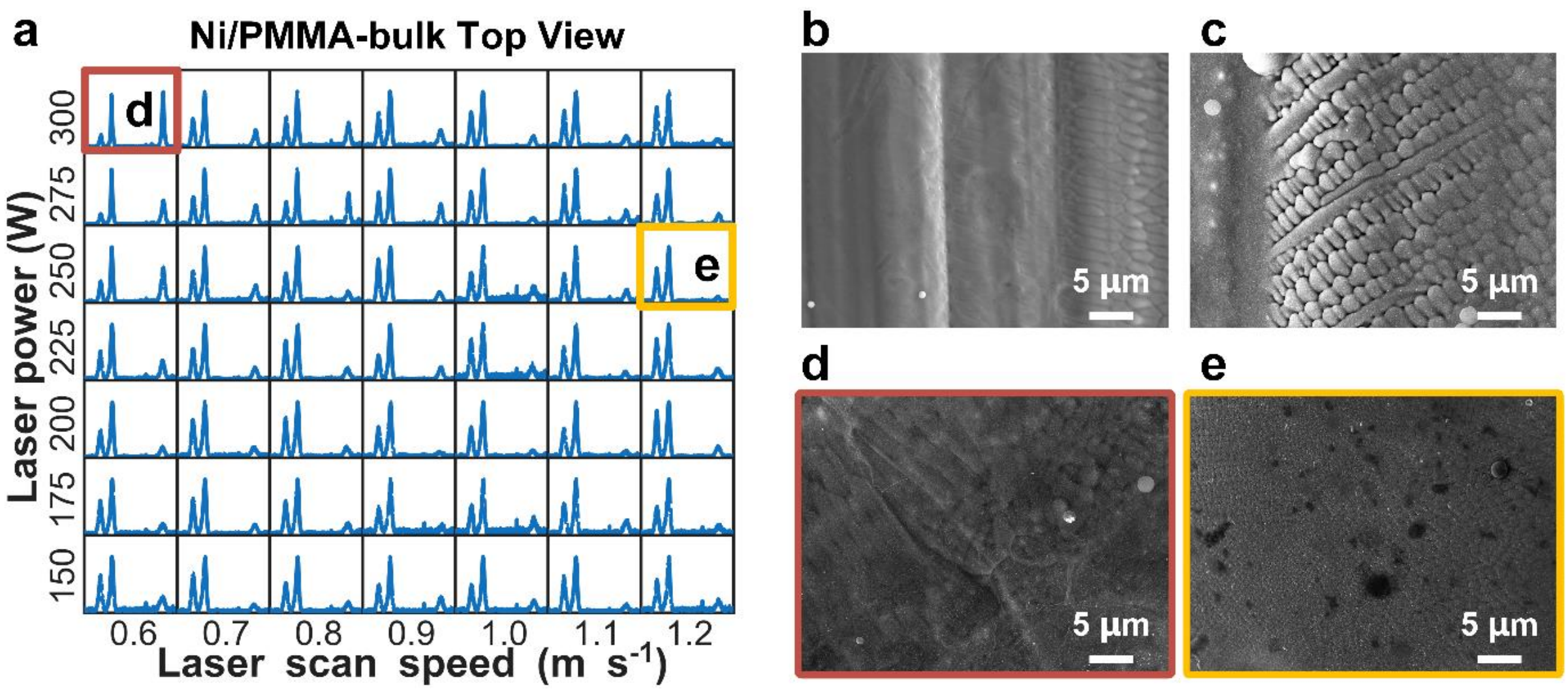

Supplementary Figure 3: Characterizations of as-grown Gr on Ni top surface with different L-PBF laser parameters.

a Representative Raman spectra of printed Ni/PMMA-Bulk top surface with various laser power and laser scan speed. **b** SEM image of L-PBF printed pure Ni surface. **c**, **d**, **e** SEM images of in-situ L-PBF printed Ni-Gr composite surfaces corresponding to lack of graphitization, uniform FLG growth, and over carbon precipitation or MLG growth, respectively. Raman spectrum at (P_l = 300 W, U_l = 0.6 m s^{-1}) with $I_{2D}/I_G \approx 1$ indicates FLG nature of Gr grown on Ni surface after L-PBF in (**d**), while Raman spectrum at (P_l = 250 W, U_l = 1.2 m s^{-1}) with $I_{2D}/I_G \approx 0.1$ shows the existing of MLG growth on Ni surface in (**e**). Scale bar, 5 μm (**b-e**). Source data are provided as a Source Data file.

Supplementary Note 3. Stoichiometric characterizations of as-grown FLG

The in-situ L-PBF synthesis technique proposed in this study for Gr-Ni composites fabrication uses PMMA as the carbon precursor. PMMA decomposes almost completely into free carbon atoms and oxygen-containing gaseous byproducts (CH_4O, CH_2O, etc.) above 500 °C[2]. Continuous graphene layers can be formed through the dissolution, segregation, and crystallization of carbon atoms on the Ni melting/solidification track.

It has been reported in the literature that the peak separation between the D'_{inf} peak and the G peak in the Raman spectrum of graphene can serve as a criterion to distinguish between graphene oxide (GO), reduced graphene oxide (rGO), and pristine graphene[3]. Raman spectra of the in-situ grown carbon phase were fitted with Lorentzian functions for the D (≈ 1350 cm^{-1}), G (≈ 1580 cm^{-1}), D'_{inf} (≈ 1620 cm^{-1}), and 2D (≈ 2700 cm^{-1}) peaks (**Supplementary Fig. 4**). The peak position difference between the D'_{inf} peak and the G band shoulder peak (Gapp) is around 28 cm^{-1}, which is greater than 25 cm^{-1}. This is definitive spectral evidence of sp^2-hybridized pristine graphene. The G peak of the in-situ grown carbon phase is sharp (FWHM = 38 cm^{-1}) and unshifted (≈ 1585 cm^{-1}), while GO typically shows a broadened and red-shifted G peak (1560-1570 cm^{-1}) due to oxygen functionalization[4].

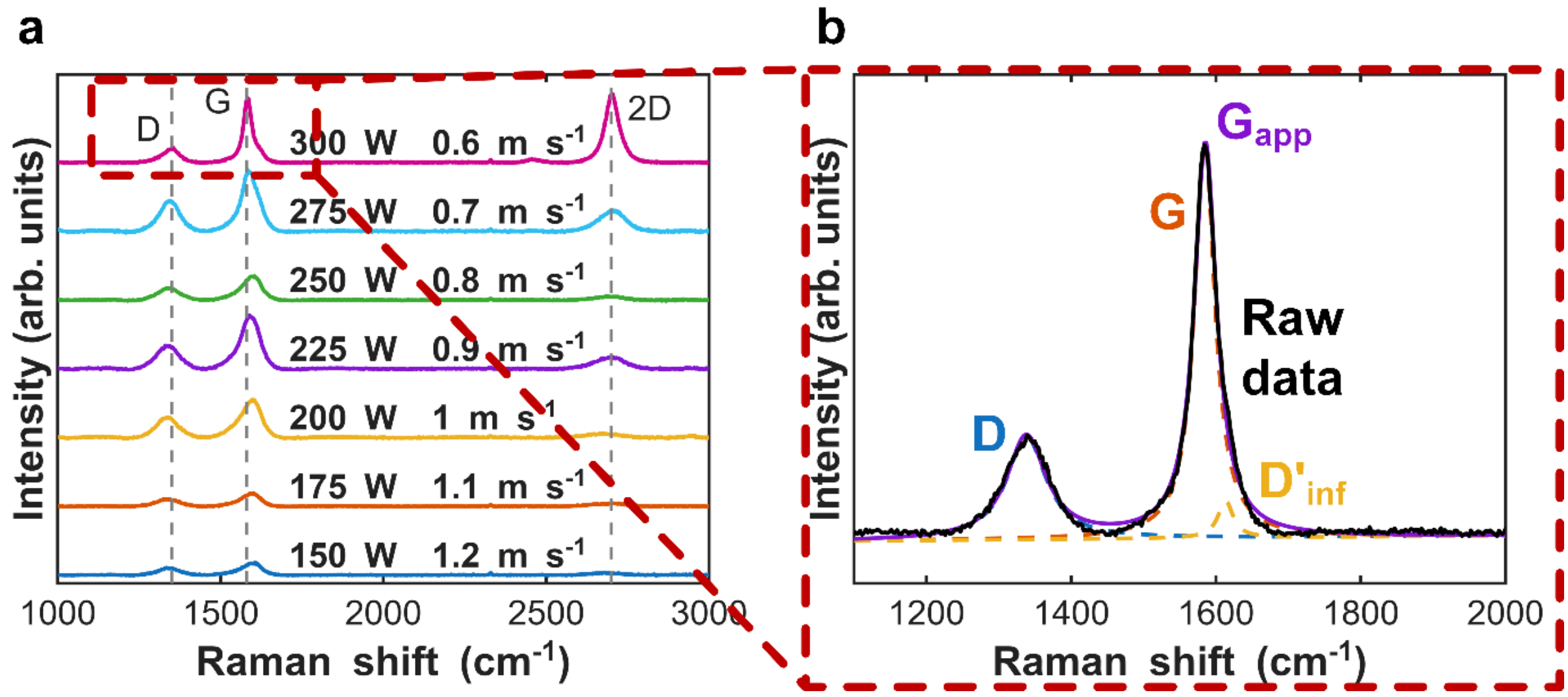

Supplementary Figure 4: Identification of in-situ grown graphene and its derivatives.

a Typical Raman spectra of the printed samples with different laser parameters. **b** A fine fit of specific sample's Raman spectrum (with a power of 300 W and a scan speed of 0.6 m s^{-1})—incorporating the D, G, and D' peaks—indicates that the graphene is of high quality and rules out the possibility of GO. Source data are provided as a Source Data file.

Supplementary Note 4. Characterizations of the Gr-Ni interface formed in L-PBF process

First-principles calculations indicate that monoatomic carbon on the Ni surface prefers to occupy subsurface octahedral interstitial sites rather than surface adsorption sites, and the chemical potential of subsurface carbon is significantly lower than that of surface carbon[5]. This thermodynamic characteristic renders carbon highly prone to diffuse into the matrix during deposition on the Ni surface and eventually form a nickel carbide phase[5], which has been confirmed by Li's team[6].

In the XPS and XRD results of Ni/PMMA-Lattice-HP sample (**Supplementary Fig. 5a-c**), there are no characteristic peaks of Ni carbides observed, for example, diffraction peaks at 39.3°, 41.7°, and 58.6° in XRD, which is respectively related to the (110), (006), and (116) planes of Ni_3C structure (PDF#06-0697). The absence of nickel carbide peak can be attributed to the high temperature during L-PBF and subsequent hot-pressing treatment, where the metastable Ni carbides could decompose, and the released carbon atoms will diffuse onto the graphene layer[7]. The ultra-low graphene content (0.095 vol.%) in the composite makes carbides undetectable by the employed characterization methods.

The cross-sectional HRTEM characterizations of the Ni/PMMA-Bulk sample helps to better understand the microstructure between the as-grown graphene and underneath Ni matrix, where the Ni carbides are most likely to form. The HRTEM images clearly show an intimate, defect-free semi-coherent interface between the Ni matrix and the graphene layers (**Supplementary Fig. 5d** and **e**). It is attributed to the high adhesion energy of graphene grown on Ni (72.7 J m^{-2}), which is around 6 times of that Gr-Cu interface[8]. Notably, the lattice fringe spacing of the Ni matrix exhibits a spacing of approximately 0.20 nm, corresponding to the (111) plane of

face-centered cubic Ni[9]. While a distinct Ni-C transition region is present, where the lattice spacing gradually shifts from 0.20 nm to 0.23 nm (**Supplementary Fig. 5f**). This gradual change indicates partial diffusion and segregation of carbon atoms at the Ni subsurface during graphene growth, forming a Ni-C interfacial layer that ensures strong adhesion and efficient load transfer across the interface.

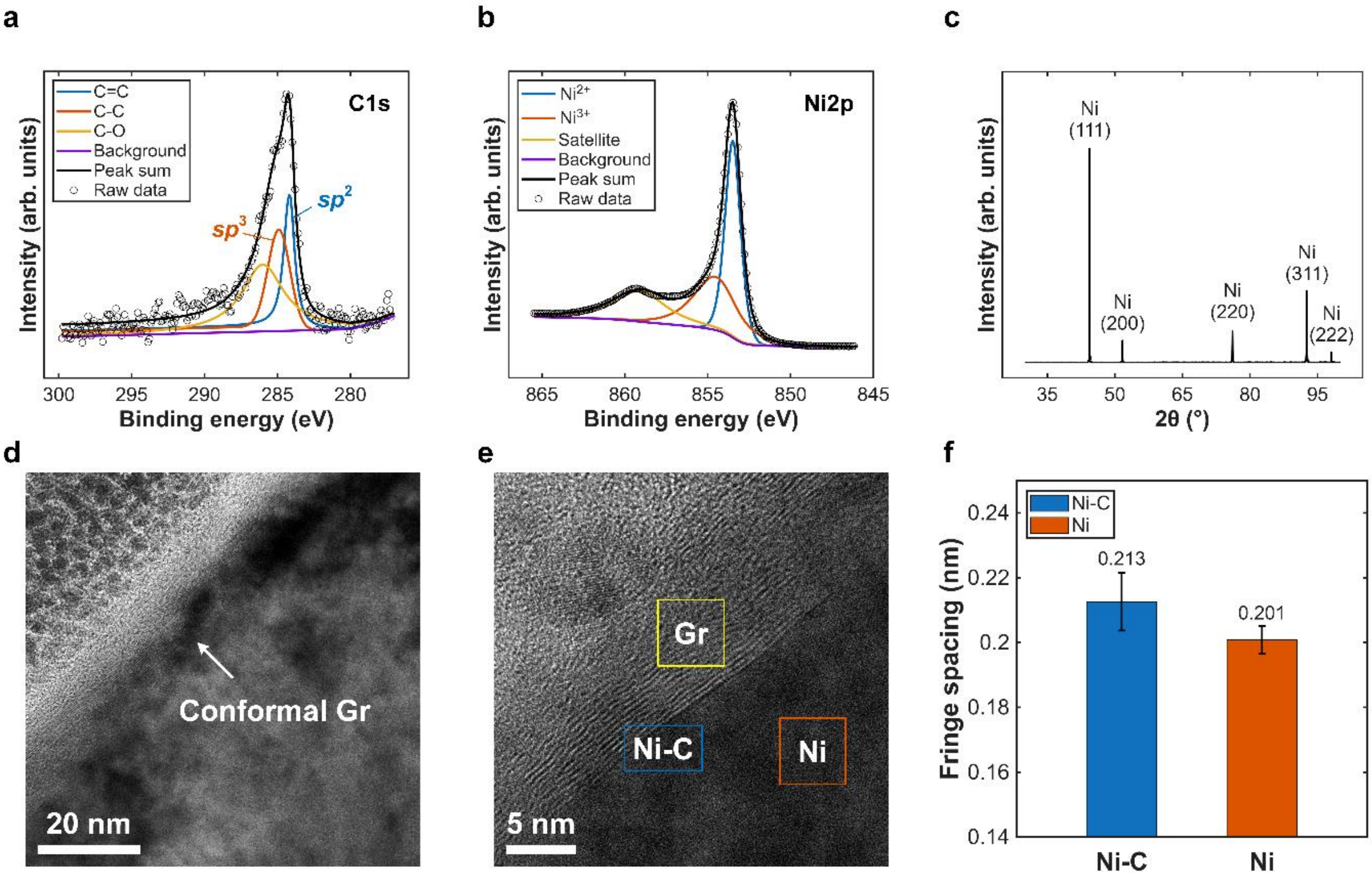

Supplementary Figure 5: Gr-Ni interface characterization.

a, **b**, **c** XPS C1s, XPS Ni2p, and XRD spectrum of Ni/PMMA-Lattice-HP sample. **d**, **e** HRTEM images of Gr-Ni interface. **f** Fringe spacing comparison of Ni region and Ni-C region. The scale bar represents the standard deviation of 15-layer lattice fringe spacing. Scale bar, 5 nm (**d**, **e**). Source data are provided as a Source Data file.

Supplementary Note 5. Transformative in-situ L-PBF strategy for various alloys

To verify the universality of the proposed in-situ graphene growth strategy during L-PBF, we extended this transformative manufacturing route from the Ni matrix to another four widely used engineering alloys, including 316L austenitic stainless steel, AlSi10Mg aluminum alloy, CuCrZr copper alloy, and Ti-6Al-4V (TC4) titanium alloy. As shown in **Supplementary Fig. 6a**, high-integrity sample arrays of all four alloy systems were successfully fabricated via L-PBF, demonstrating the process compatibility of our strategy with mainstream commercial metal AM materials. Raman spectroscopy analysis was performed (**Supplementary Fig. 6b** and **c**), illustrating the graphene growth quality as a function of laser power and scan speed, confirming that a well-defined process window for in-situ graphene growth can be identified for each alloy via tuning the melt pool thermal history. The characteristic Raman spectra for all four alloys exhibits the distinct D peak (~1350 cm^{-1}), G peak (~1580 cm^{-1}), and 2D peak (~2700 cm^{-1}) of graphene, providing unambiguous evidence for the successful in-situ synthesis of graphene on the printed surface of all investigated alloy systems via the same in-situ L-PBF route.

The distinct subsurface carbon solubility[9] and carbon chemical potential[5] across various metallic matrices result in divergent growth mechanisms. The moderate carbon solubility in 316L leads to a graphene growth pattern similar to that of Ni, which follows the sequence of carbon dissolution, segregation, and crystallization. In contrast, AlSi10Mg and CuCrZr exhibit extremely low equilibrium carbon solubility in their matrices; bulk dissolution of carbon atoms remains negligible even at molten pool temperatures[9,10]. For these two alloy systems, graphene growth proceeds via a surface adsorption and surface reaction mechanism[11]. For TC4, the

exceptionally high chemical affinity between Ti and C readily induces the formation of brittle TiC precipitates in the matrix, which competes with the growth of surface graphene[12]. Therefore, precisely tuning laser parameters to promote the rapid segregation and nucleation of carbon atoms while inhibiting carbide precipitation is likely the key to controlling the quality of graphene growth on titanium alloy.

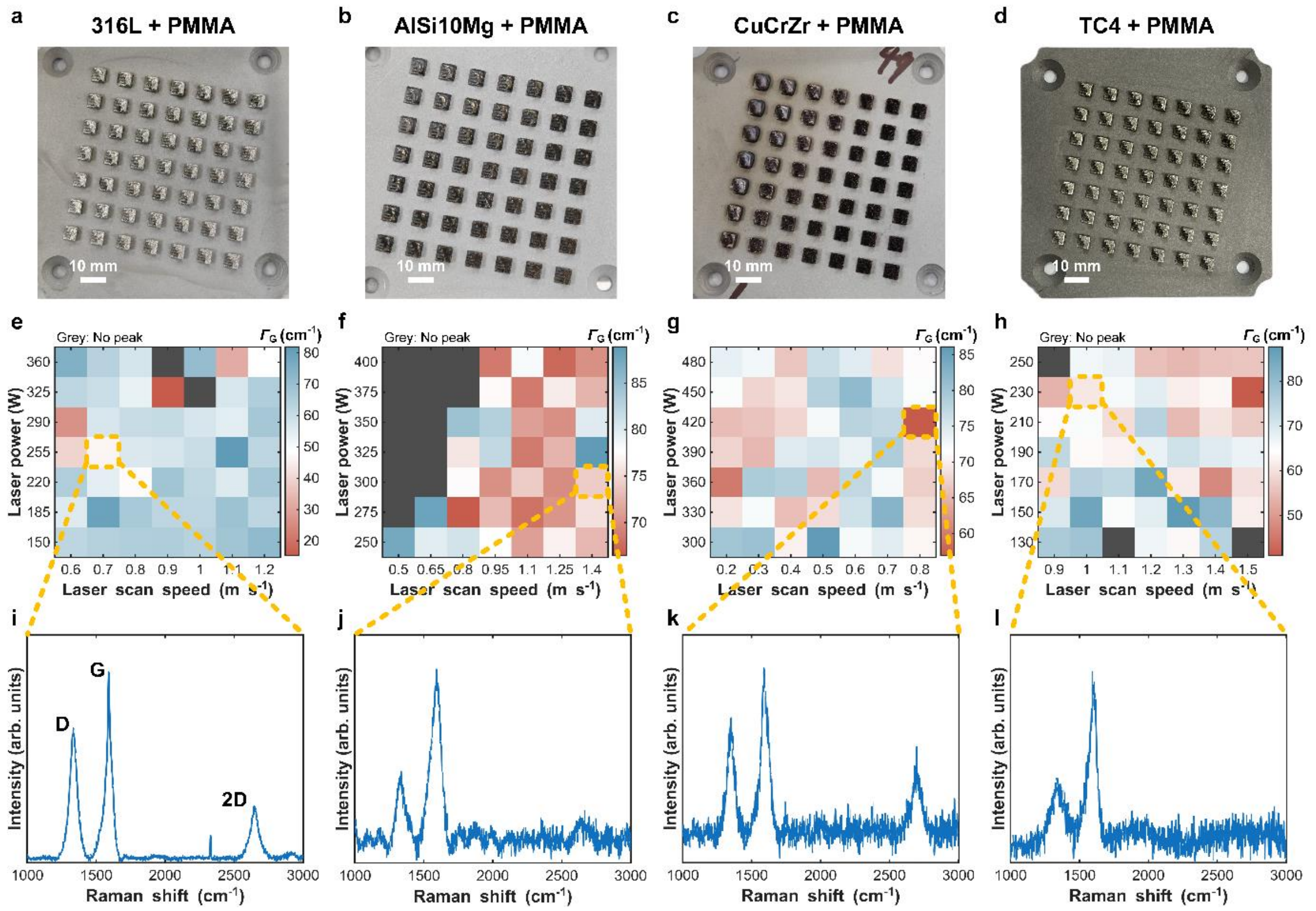

Supplementary Figure 6: Raman results of as-printed Metal/PMMA-Bulk samples.

a, **b**, **c**, **d** Photos of the as-printed specimens of 316L/PMMA-Bulk, AlSi10Mg/PMMA-Bulk, CuCrZr/PMMA-Bulk, and TC4/PMMA-Bulk from the pre-blended metal/PMMA powder, respectively. Scale bar, 10 mm (**a-d**). **e**, **f**, **g**, **h** The Raman G peak's full width at half maximum (Γ_G) distribution corresponding to the 49 L-PBF laser conditions in the printed samples in (**a-**

d). **i**, **j**, **k**, **l** The representative Raman spectra in (**e**-**h**). Source data are provided as a Source Data file.

Supplementary Note 6. Structural characterizations of the grown FLG on the printed Ni scaffold surface during L-PBF process and within the Ni matrix after densification

Extensive Raman spectroscopy characterizations demonstrate the high-quality growth of graphene on various printed architectures. For example, the graphene coverage rate exceeds 98% on the top surface and 94% on the side surfaces of Ni/PMMA-Bulk samples (**Supplementary Fig. 7** and **Supplementary Fig. 8**). Meanwhile, continuous high-quality graphene growth is also realized on the as-printed composite Gyroid skeleton (**Supplementary Fig. 9** and **Supplementary Fig. 10**).

In addition, EBSD KAM results of Ni/PMMA-Lattice-HP exhibit a periodic pattern of high GND density that matches well with the Gyroid geometry (**Fig. 4j** and **Supplementary Fig. 11**). This typical pattern arises from thermal stress differences between the printed Ni framework and consolidated powder regions, together with dislocation blocking at their interfaces. At these interfaces, the growth of a three-dimensional continuous graphene network has been confirmed by Raman and SEM characterizations. Furthermore, hot pressing does not damage the continuity and integrity of the 3D graphene network in Ni/PMMA-Lattice-HP samples. Accordingly, FEM simulations were carried out to illustrate the stress-strain distribution between the Ni skeleton and Ni/PMMA powder during compressive deformation.

A 2×2×2 representative volume element model is established to simulate the deformation with a symmetric boundary condition. Both Ni/PMMA-Bulk and powder matrix are meshed with linear tetrahedral finite element. Elasto-plastic model is applied to simulate the constitutive behavior of both parts. The parameters are obtained from experiments and a reduction based on the packing density for Ni skeleton and Ni/PMMA powder respectively. The model is loaded

with a rigid plate meshed with quadrilateral shell element with general contact, up to a strain of 20%, according to the actual experimental value. The model is shown in **Supplementary Fig. 12a**. The explicit method is conducted for FEM analysis and mass scaling is applied for higher computing efficiency. Additionally, the total mass increment ratio is restrained below 1%, leading to a rational result.

FEM results are shown in **Supplementary Fig. 12b** and **c**. The deformed configuration maintains the gyroid-like curvy shape, which keeps a good consistency with EBSD KAM results (**Supplementary Fig. 4j** and **Supplementary Fig. 11**). The stress concentrates at the peaks that tangents parallel to loading direction, and between the peaks, the stress level is much lower. As shown in **Supplementary Fig. 12c**, the maximum true strain in skeleton structure is about 0.3, which obviously exceeds the ductility of graphene. However, between two peak values, the strain of the most region remains quite low at around 0, which partially keeps the connectivity of graphene layers, leaving continuous 3D Gr network in Ni/PMMA-Lattice-HP composite after hot pressing.

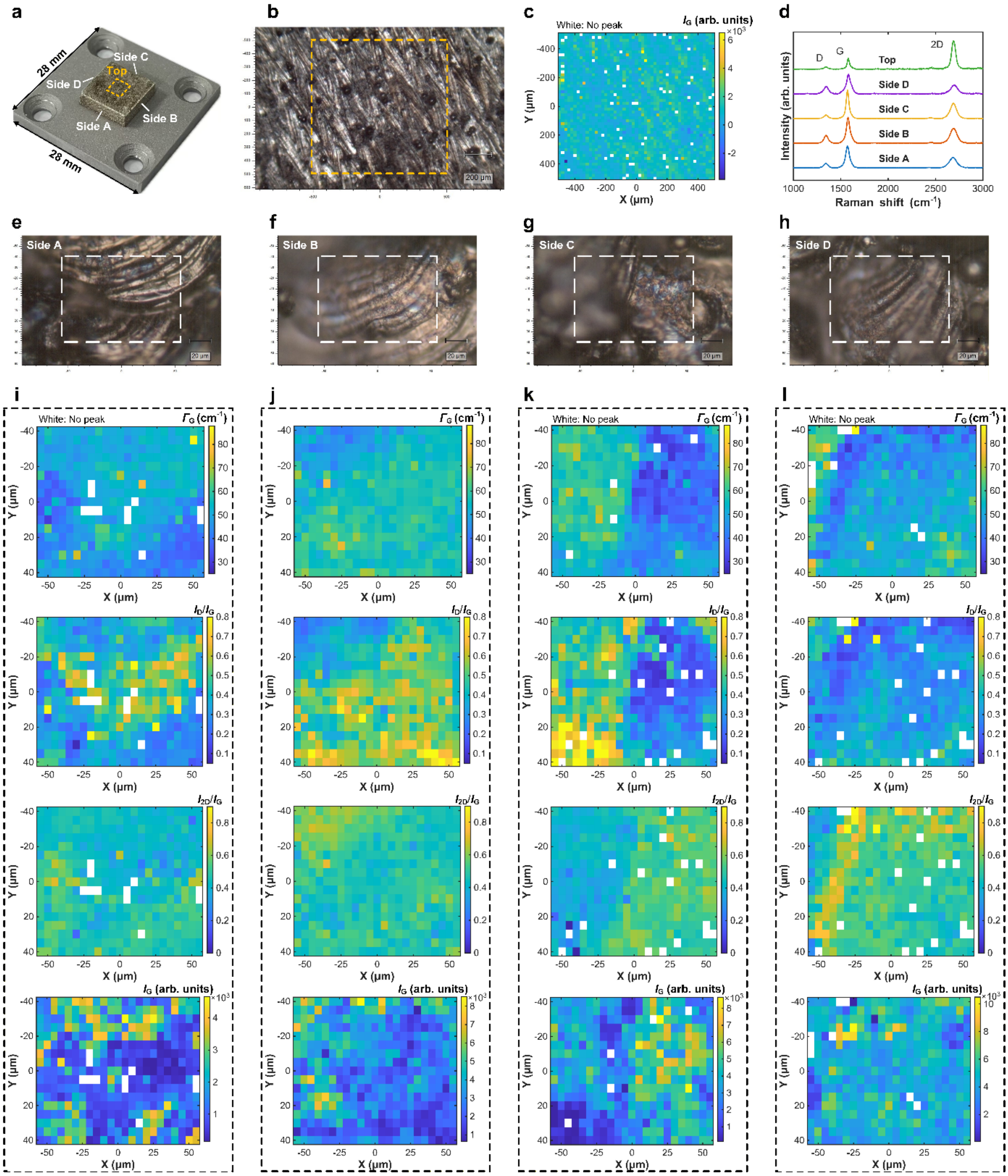

Supplementary Figure 7: Raman results for the top and four lateral surfaces of the Ni/PMMA-Bulk sample.

a As-printed Ni/PMMA-Bulk sample photo. **b** OM of Ni/PMMA-Bulk sample's top surface. **c** Raman G peak intensity map of the dashed square in (**b**). **d** Representative Raman spectra of Ni/PMMA-Bulk sample's top surface and 4 lateral surfaces. **e, f, g, h** OM of Ni/PMMA-Bulk

sample’s 4 lateral surfaces. **i, j, k, l** Raman mapping results of Ni/PMMA-Bulk sample’s 4 lateral surfaces. Source data are provided as a Source Data file.

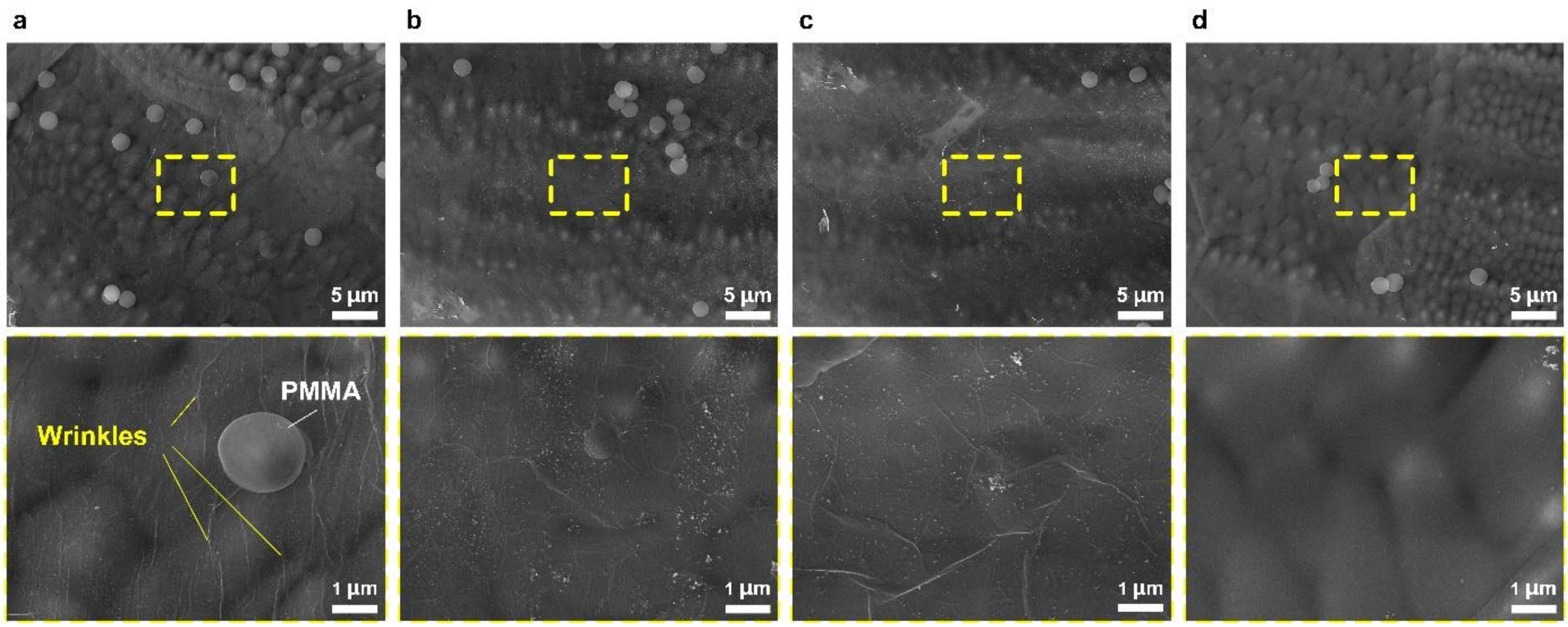

Supplementary Figure 8: SEM images of the grown FLG on the printed Ni.

a, **b**, **c**, **d** SEM images of Ni/PMMA-Bulk samples' top surface. The yellow lines in (**a**) point to the wrinkled microstructures of the graphene on the printed surface, while the white lines point to incompletely pyrolyzed PMMA particle. Scale bar, upper 5 µm, lower 1 µm **(a**, **b**, **c**, **d**).

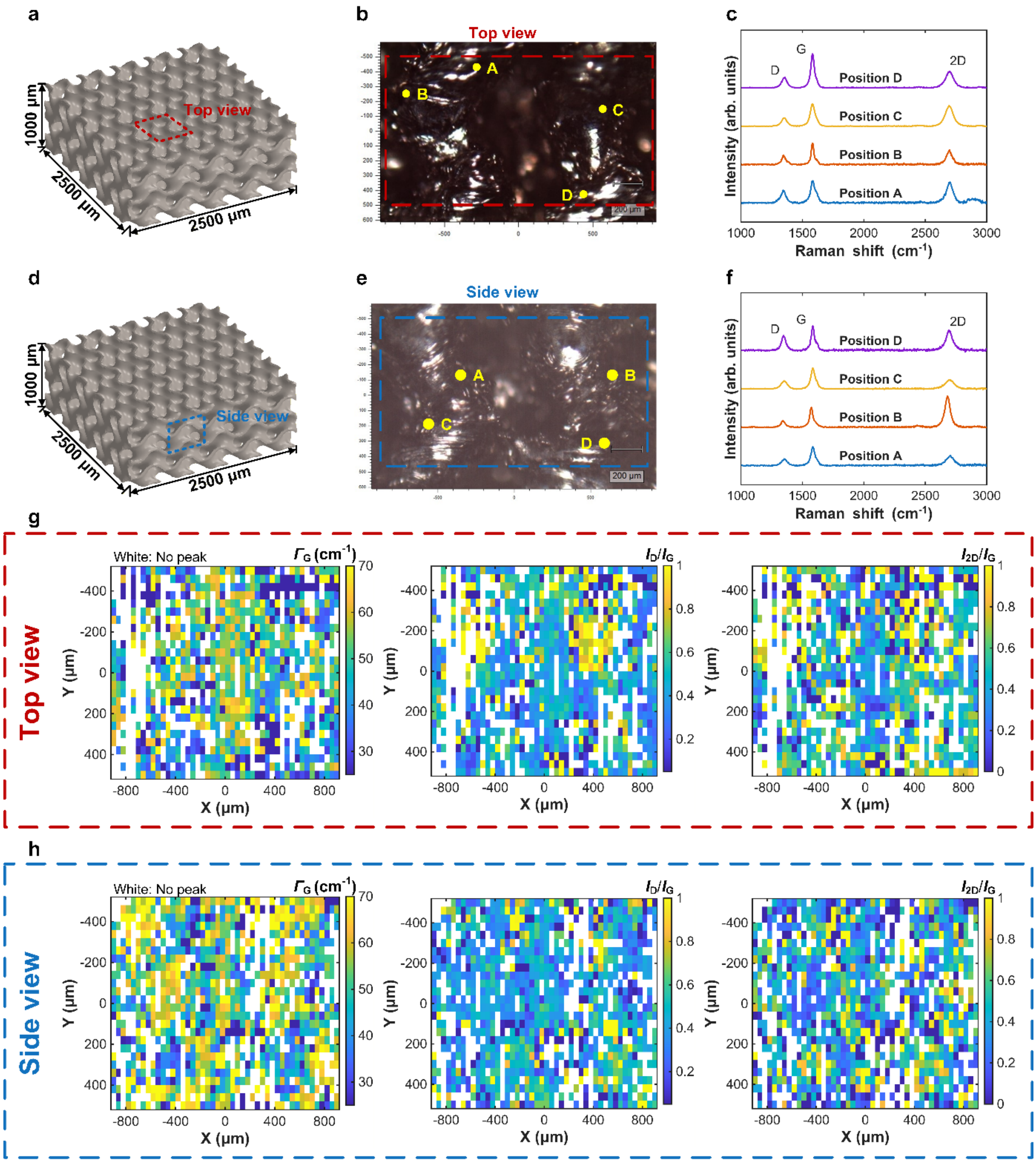

Supplementary Figure 9: Characterizations of FLG grown on the curved surface of a printed gyroid Ni structure.

a, d Gyroid structure 3D model. **b, e** OM of printed gyroid Ni-Gr composite's top surface and lateral surface. **c, f** Representative Raman spectra of different position in (**b**) and (**e**), respectively. **g, h** Corresponding Raman results in (**b, e**). Source data are provided as a Source Data file.

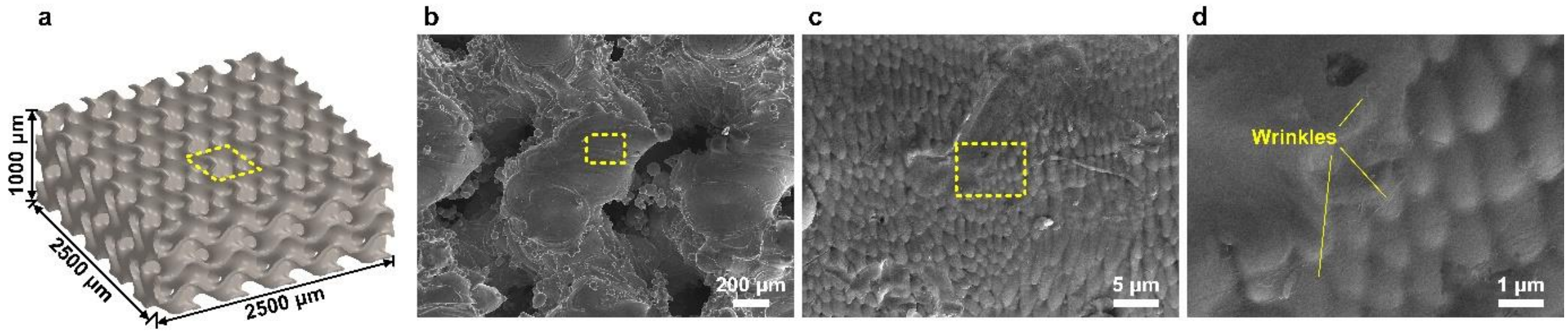

Supplementary Figure 10: SEM images of in-situ grown graphene on as-printed gyroid skeleton.

a Gyroid structure 3D model. **b**, **c**, **d** SEM images of as-printed composite skeleton, where graphene uniformly grows on its surface and yellow lines point to the unique wrinkle structure of graphene. Scale bar, 200 μm (**b**), 5 μm (**c**), 1 μm (**d**).

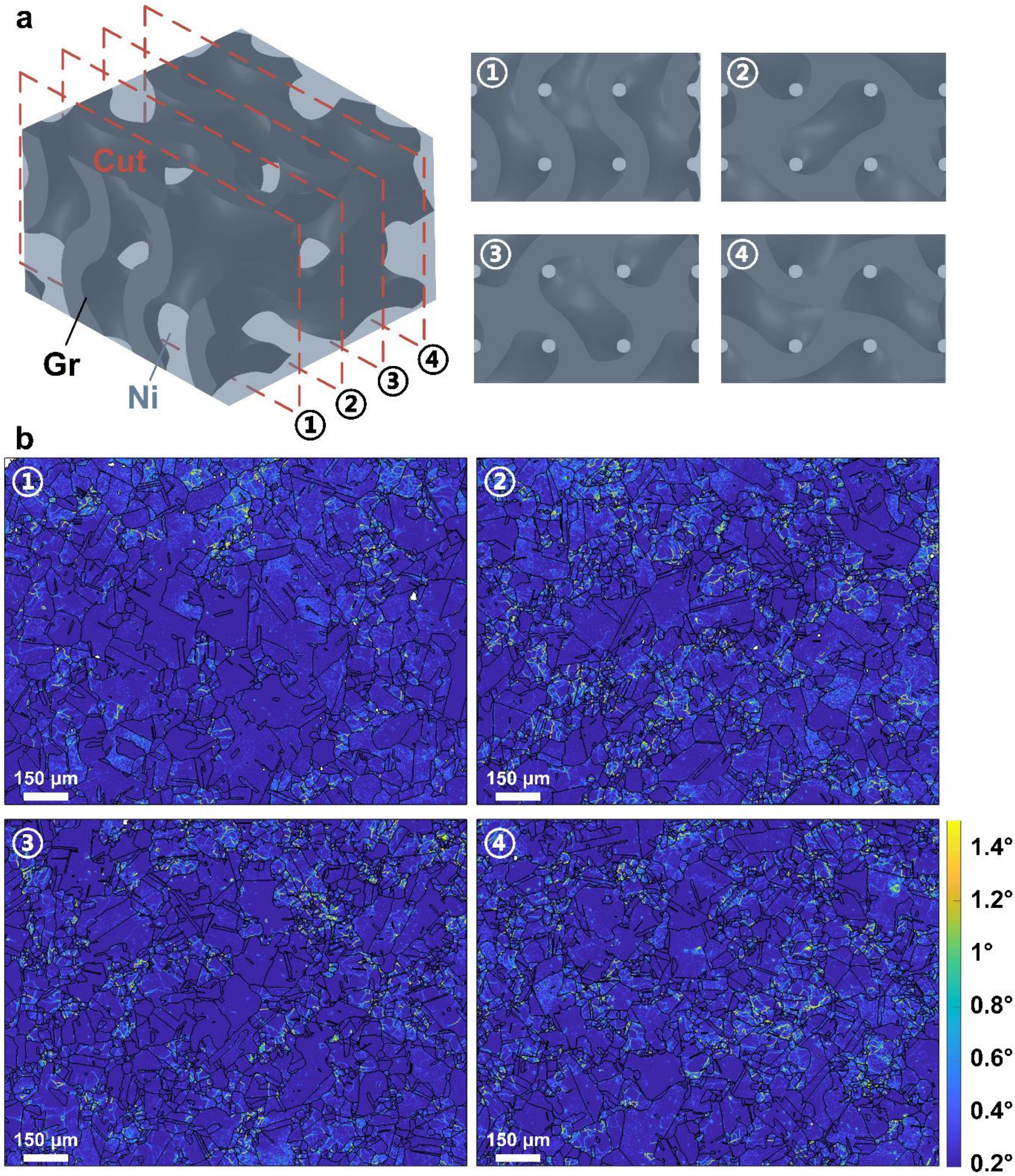

Supplementary Figure 11: KAM images of Ni/PMMA-Lattice-HP sample.

a Ni/PMMA-Lattice-HP KAM sample cutting schematic, showing the possible distribution of the graphene network. **b** Corresponding KAM images in (**a**). Scale bar, 150 μm (**b**).

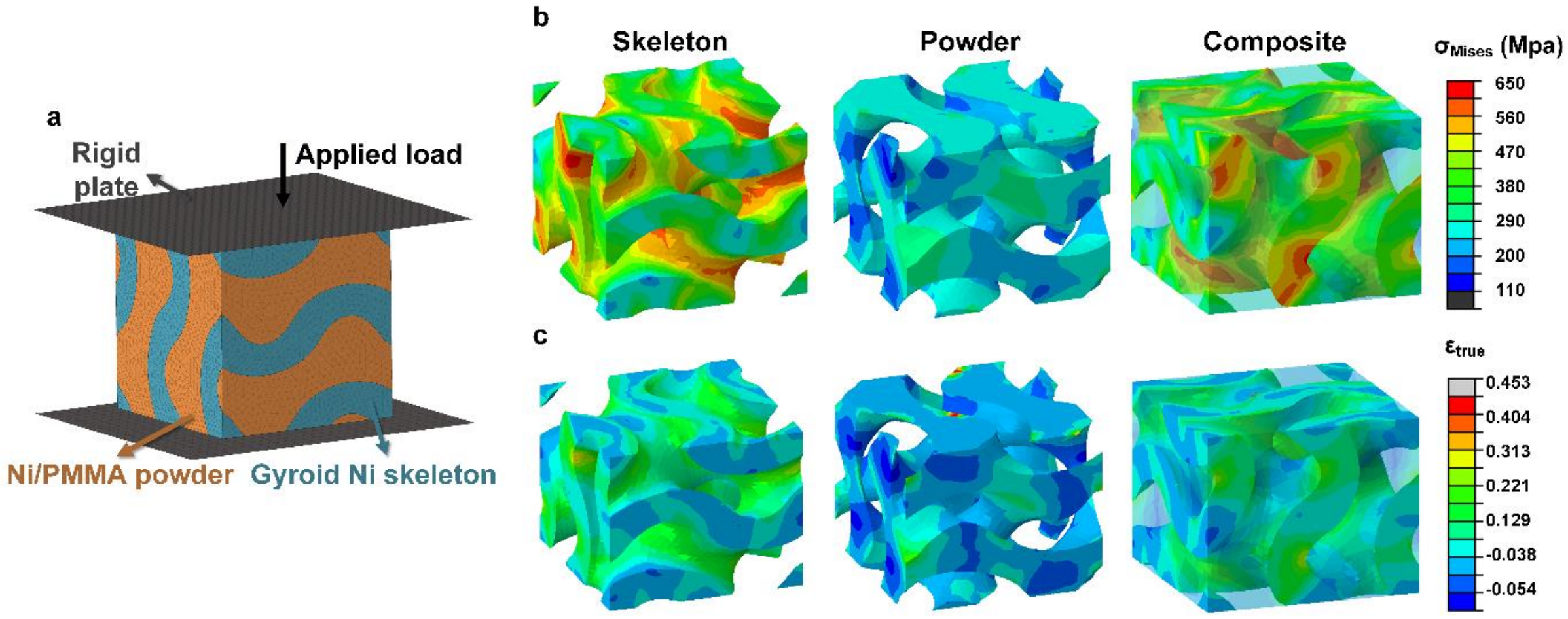

Supplementary Figure 12: Simulated compression deformation of Ni skeleton-Ni/PMMA powder composite.

a Finite element method model. **b**, **c** Von Mises stress and true strain of Ni skeleton, Ni/PMMA powder, and composite during compression deformation.

Supplementary Note 7. Mechanism of in-situ grown FLG for microcrack suppression and thermal field regulation

Microcracks in L-PBF-fabricated pure metals are primarily induced by uneven thermal stress during rapid heating/cooling and directional solidification[13,14]. The in-situ grown FLG has an ultrahigh elastic modulus and excellent in-plane thermal conductivity (>3000 W m^{-1} K^{-1}), which could facilitate temperature uniformity in the Ni cladding during L-PBF printing. Firstly, as shown in **Supplementary Fig. 13**, the existing graphene layer will be distorted and fragmented when the second Ni matrix layer is fabricated, and the graphene fragments embedded in the Ni melt pool could homogenize the local temperature field in the melt pool and the solidified cladding, reducing the thermal gradient and the resulting thermal stress. Second, the weak van der Waals interactions between FLG layers could allow for slight slippage under thermal stress, which dissipates the concentrated stress and prevents the initiation of microcracks and their propagation.

It has been confirmed that the extreme, non-uniform transient thermal gradient and the mismatched cooling and contraction between adjacent regions/layers during melting-solidification in laser AM, are the root causes of residual stresses and geometric distortion in L-PBF AM parts[15,16]. In fact, the in-plane thermal conductivity of graphene and its derivatives is significantly higher than that of bulk Ni, especially at high temperatures[17,18], which can effectively improve the in-plane temperature field uniformity during L-PBF AM cooling stage, thereby mitigating the in-plane thermal inhomogeneity induced by the laser scanning trajectories, and consequently reducing residual stress and property anisotropy.

To validate the above hypothesis, we employed high-speed IR thermal imaging to in-situ monitor the transient thermal behavior and melt pool thermal history of the pure Ni powder bed and the Ni/PMMA powder bed during the L-PBF process, with the corresponding results presented in **Supplementary Fig. 16**. The reconstructed IR temperature field (**Supplementary Fig. 16a** and **b**) revealed evident differences in the spatial thermal distribution between the two systems. The pure Ni sample exhibited a relatively diffused high-temperature region with pronounced transverse thermal diffusion, leading to a broadened thermal affected zone. In comparison, the Ni/PMMA system featured a more confined high-temperature zone, where the temperature dropped more rapidly away from the melt pool center, indicating more efficient heat dissipation from the laser irradiation region. Quantitative analysis of the line-scan temperature profiles across the melt pool (**Supplementary Fig. 16c**) further highlighted these differences. The peak melt pool temperature of the pure Ni system reached approximately 2820 K, which was notably higher than that of the Ni/PMMA system (~2580 K), demonstrating that the introduction of the FLG precursor effectively mitigated local overheating during laser irradiation. More importantly, the temperature difference between the melt pool center and the surrounding solidified region was significantly reduced in the Ni/PMMA system. As highlighted in the magnified inset of the temperature profile, within the cooling region behind the melt pool, the Ni/PMMA sample exhibited a more uniform temperature evolution. These experimental observations directly confirm that the in-situ formed FLG can effectively homogenize the in-plane temperature field during the L-PBF process, which alleviates the extreme transient thermal inhomogeneity induced by the localized laser heating and scanning trajectories. This provides solid experimental evidence supporting our proposed mechanism.

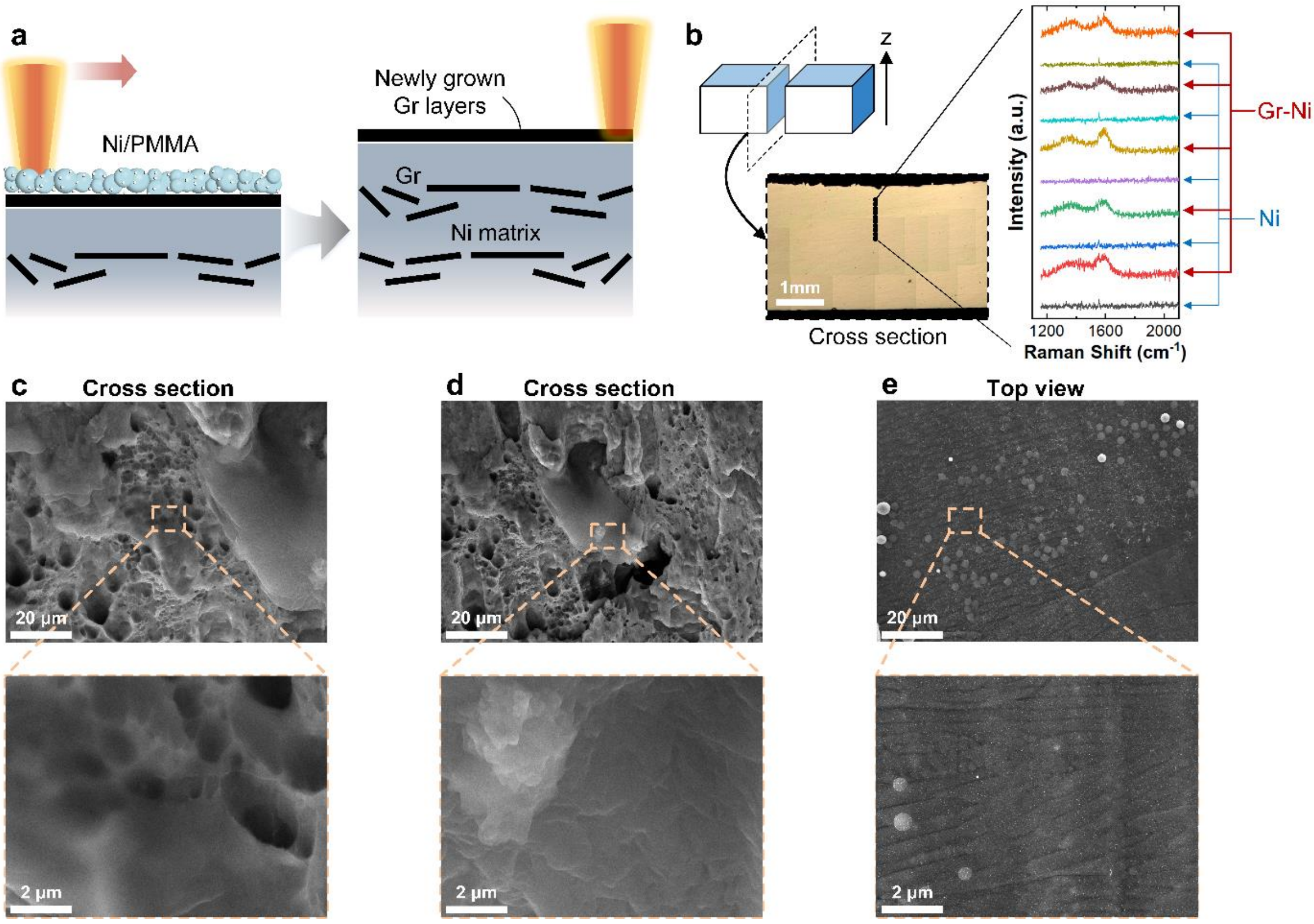

Supplementary Figure 13: The as-grown Gr distribution within the Ni/PMMA-Bulk.

a Schematic illustration showing that as-grown FLG on the surface of cladding was torn and distorted as the laser melting of the next layer of powders, and a new layer of Gr grew on the top surface of cladding. **b** OM image and Raman spectra of the cross-sectional surface of the printed Ni-Gr composite, showing periodic graphitization phenomenon, scale bar 1 mm (**b**). **c**, **d** SEM images of the cross-section of the printed Ni-Gr composite. The zoomed-in SEM images highlight the torn Gr flakes with different thicknesses. **e** The top-view SEM images of the printed Ni-Gr composite. Scale bar, 20 μm for the top panel, 2 μm for the bottom panel (**c-e**).

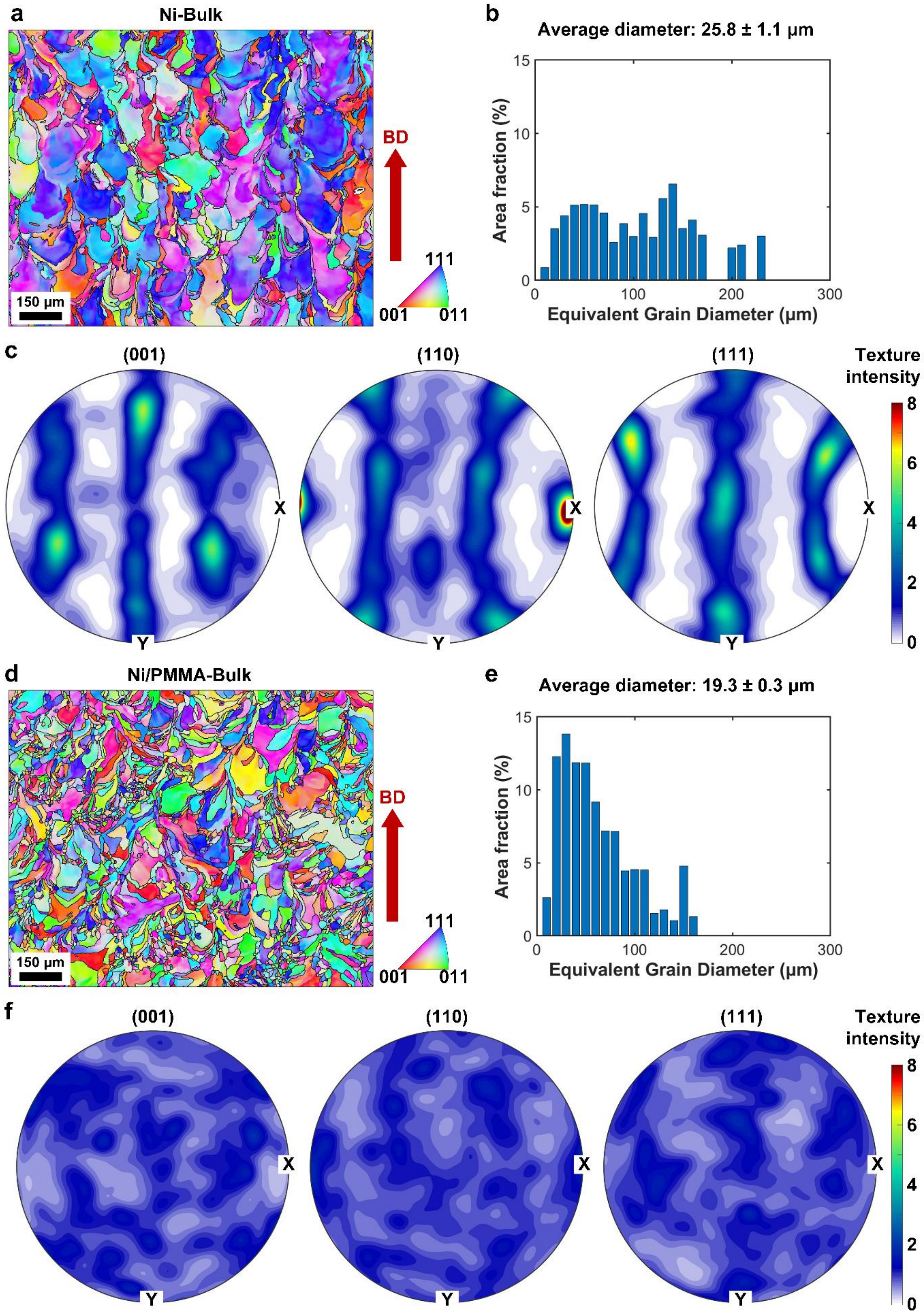

Supplementary Figure 14: Comparison of microstructures of Ni-Bulk and Ni/PMMA-

Bulk.

a, **d** EBSD IPF maps of L-PBF printed pure Ni and Ni/PMMA-Bulk composite, respectively. For Ni/PMMA-Bulk composite, hierarchical grain structures consisting of coarse columnar grains and finer equiaxed grains near the melt pool boundaries were observed, compared to columnar grain dominated microstructure for pure Ni. Scale bar, 150 μm (**a**, **d**). **b**, **e** Grain size distributions derived from EBSD IPF maps. The grain size data are presented as mean ± standard error. **c**, **f** EBSD PF maps of L-PBF printed pure Ni and Ni/PMMA-Bulk composite, indicating the clear texture microstructure of L-PBF printed Ni due to strong directional thermal gradient during layer-by-layer building, while a more homogeneous microstructure when Gr grew in-between layers. Source data are provided as a Source Data file.

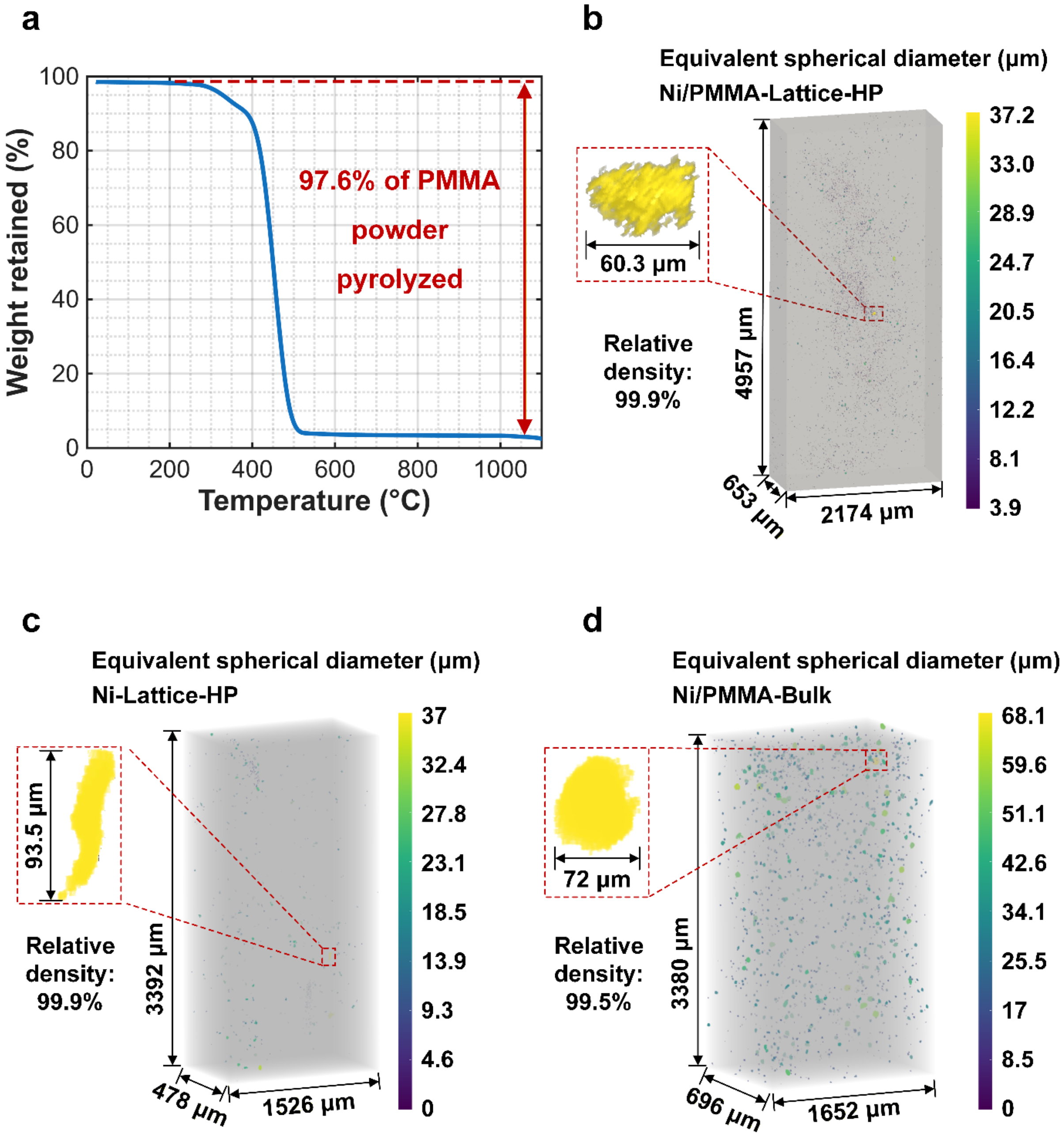

Supplementary Figure 15: TGA and CT analysis of the fabricated Gr-Ni composites.

a The TGA analysis showing 97.6% of PMMA powders can be fully pyrolyzed at temperatures over 500˚C. **b**, **c**, **d** CT analysis of Ni/PMMA-Lattice-HP, Ni-Lattice-HP, and Ni/PMMA-Bulk samples showing 99.9%, 99.9%, and 99.5% relative density respectively. Pores are color-coded by equivalent spherical diameter. Source data are provided as a Source Data file.

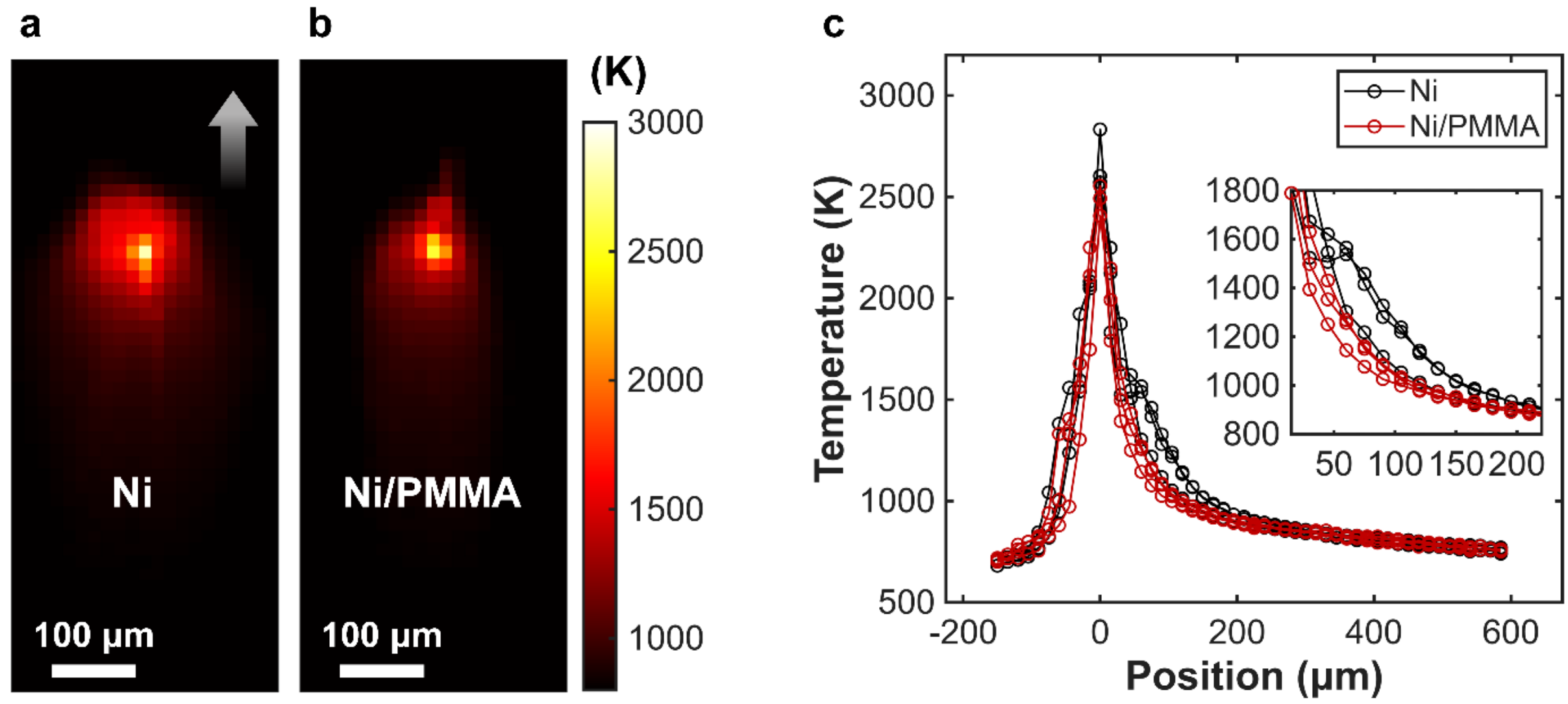

Supplementary Figure 16: In-situ IR monitoring of Ni and Ni/PMMA melt pool.

a, **b** Reconstructed infrared (IR) images of melt pool of Ni and Ni/PMMA respectively. The gray arrows represent the laser scanning direction. **c** Typical melt pool's and the solidifying cladding's temperature profile of Ni and Ni/PMMA. Scale bar, 100 μm (**a**, **b**). Source data are provided as a Source Data file.

Supplementary Note 8. Quantitative strengthening mechanism

The enhanced yield strength Ni/PMMA-Lattice-HP compared with Ni-Lattice-HP can be estimated by equation:

$$\sigma_C = \sigma_M + \Delta\sigma_{Hall-petch} + \Delta\sigma_{SS} + \Delta\sigma_{DS} + \Delta\sigma_{LT} \quad (1)$$

where σ_C is the yield strength of composite, σ_M is yield strength of matrix. The difference between them can be attributed to the Hall-petch strengthening $\Delta\sigma_{Hall\text{-}petch}$, solid solution strengthening $\Delta\sigma_{SS}$, dislocation strengthening $\Delta\sigma_{DS}$, and load transfer strengthening $\Delta\sigma_{LT}$.

8.1 Hall-petch strengthening

Hall-Petch strengthening, also known as grain boundary strengthening, originates from the hindrance effect of grain boundaries on dislocation slip and propagation. The quantitative contribution of Hall-petch strengthening can be calculated by the classical Hall-petch relationship[19]:

$$\Delta\sigma_{Hall-petch} = K\left(d_C^{-0.5} - d_M^{-0.5}\right) \quad (2)$$

where K is the Hall-Petch slope constant for pure Ni, with a value of 154.95 MPa·μm$^{0.5}$, d_C and d_M are the average grain size of composite and Ni matrix respectively. This negligible, even negative contribution is attributed to the ultra-low loading content of graphene in the composite, which only induces local grain refinement in limited regions, while the overall average grain size of the Ni matrix in the composite shows no significant refinement.

8.2 Solid solution strengthening

Solid solution strengthening arises from the lattice distortion induced by the dissolution of solute atoms into the metal matrix lattice. Solute atoms with a different atomic radius from the matrix atoms generate a local elastic strain field around their lattice sites, which interacts with

moving dislocations, impedes dislocation glide, and thus improves the strength of the material. It can be calculated by the following equation:

$$\Delta\sigma_{ss} = MG\varepsilon^{\frac{3}{2}}\sqrt{\frac{f_m}{3}} \tag{3}$$

where M is the Taylor factor for Ni, taken as 3.1, G is the shear modulus of pure Ni, with a value of 65 GPa, ε is the lattice misfit parameter caused by interstitial carbon atoms in the Ni lattice, with a value of 0.0152[20]. f_m is the mass concentration of carbon dissolved in the Ni matrix, which is estimated according to the equilibrium solid solubility limit of carbon in Ni at room temperature (1×10^{-6} wt.%)[21]. Based on the above parameters, the calculated contribution of solid solution strengthening is ~0.21 MPa, which could be ignored.

8.3 Dislocation strengthening

The quantitative contribution of dislocation density can be calculated by the classic Taylor equation[20]:

$$\Delta\sigma_{ds} = \alpha MGb\sqrt{\rho_0} \tag{4}$$

where α is a dimensionless constant related to dislocation interaction, taken as 0.25, M is the Taylor factor (3.1), G is the shear modulus of Ni (65 GPa), b is the Burgers vector of Ni, with a value of 0.249 nm[22]. Dislocation density ρ_0 can be estimated as:

$$\rho_0 = \frac{2\sqrt{3}}{3}\frac{\theta}{bs} \tag{5}$$

where θ is the average misorientation angle per unit step length obtained from KAM mapping, and s is the step size of the EBSD scanning. Based on the experimental KAM data and the above parameters, the calculated contribution of dislocation strengthening is 13.9 MPa.

8.4 Load transfer strengthening

Load transfer strengthening is the most characteristic and dominant strengthening mechanism in graphene-reinforced metal matrix composites. The quantitative contribution of load transfer strengthening can be evaluated by different methods. In the shear-lag model, it is described as[23]:

$$\Delta\sigma_{LT} = \frac{S}{4} f_v \sigma_{YM} \tag{6}$$

where S is defined as the aspect ratio of the reinforcing phase. For the graphene reinforcement in our composite system, its in-plane lateral diameter can be approximated as 100 μm based on the characteristic feature size of the gyroid skeleton, and the thickness of the graphene nanosheets is determined to be 100 nm. The term f_v refers to the volume fraction of graphene (0.095 vol.%). Using the above parameters, the load transfer strengthening is calculated to be 29.6 MPa.

8.5 Load transfer strengthening mechanism of 3D graphene network

Load transfer strengthening is identified as the dominant strengthening mechanism in the composite (Ni/PMMA-Lattice-HP), contributing 29.6 MPa to the total yield strength increment as calculated by the modified shear-lag model. It accounts for more than 70% of the total theoretically calculated strengthening effect. However, the classical shear-lag model adopted for theoretical calculation is established based on the core assumption of discrete, randomly distributed 2D graphene nanosheets in the metal matrix, which fundamentally differs from the 3D continuous, interpenetrating graphene network architecture in our composite system. The unique topological structure of the in-situ grown 3D graphene network enables a more efficient load transfer behavior, which is the core reason for the underestimation of the load transfer strengthening contribution by the traditional theoretical model, as well as the gap between the total calculated strength increment (41.2 MPa) and the experimental value (53.8 MPa), as

shown in **Supplementary Fig. 17**.

The primary advantage of the 3D graphene network in load transfer lies in its ability to form a robust mechanical interlocking structure with the Ni matrix, which significantly enhances the interfacial shear strength and the efficiency of stress transfer from the soft metal matrix to the high-strength graphene reinforcement. Molecular dynamics simulation results from Yang et al.[24] demonstrated that the interlocking structure formed by the 3D graphene network and the metal matrix improves the deformation compatibility between the reinforcement and the matrix, and the average interfacial pull-out force of the 3D graphene network in the Ni matrix is approximately three times higher than that of discrete 2D graphene sheets. In this work, the in-situ grown graphene conformally wraps the 3D printed gyroid Ni skeleton, forming an intimate Ni-graphene interface. This conformal growth further consolidates the mechanical interlocking between the 3D graphene network and the Ni matrix, ensuring that the external load applied to the composite can be efficiently and continuously transferred to the graphene reinforcement throughout the bulk material, rather than being limited to the local shear zone around individual graphene flakes.

Unlike discrete 2D graphene sheets that rely solely on interfacial shear stress to achieve load transfer, the 3D continuous graphene network can directly bear the external load through its integral skeleton structure, achieving long-range, full-scale load sharing in the composite. For conventional Gr-MMCs with discrete flake reinforcements, the load transfer process is restricted to the short-range shear zone at the graphene-matrix interface, and the reinforcing effect is severely limited by the in-plane size of individual graphene flakes; once interfacial debonding or graphene pull-out occurs, the load transfer capacity of the reinforcement is almost

completely lost[23]. In contrast, the 3D graphene network in our composite forms a continuous, self-supporting reinforcing skeleton that interpenetrates with the Ni matrix. Even if local interfacial separation occurs at partial regions during tensile deformation, the integral network structure can still maintain the load-bearing capacity through the continuous interconnected skeleton, and exert an extrinsic toughening effect via crack bridging, which further amplifies the load transfer strengthening effect. Yang et al.[7] also pointed out that the continuous network structure enables graphene to bear load directly in addition to interfacial load transfer, which effectively compensates for the strength reduction caused by local interfacial separation, and the straight portions of the 3D graphene network exhibit a more efficient load-sharing capacity during tension.

Furthermore, the unique deformability of the 3D graphene network optimizes the stress distribution during composite deformation, further improving the stability and efficiency of the load transfer process. MD results revealed that the curved portions of the 3D graphene network can be straightened during tensile deformation, which enables the network to release local stress concentrations caused by dislocation blocking through overall structural deformation, avoiding the premature fracture of the reinforcement caused by local stress overloading[7]. This stress relaxation effect ensures that the load can be uniformly distributed on the 3D graphene network, maintaining the long-term effectiveness of the load transfer process during the entire plastic deformation stage. Meanwhile, the straightening of the curved portions of the graphene network enhances the mechanical interlocking between the network and the Ni matrix through dislocation-graphene interactions, which further increases the interfacial shear stress and the load-sharing ratio of the graphene reinforcement. In contrast, when dislocations encounter

discrete 2D graphene sheets, stress concentration is prone to occur at the edges of the sheets, leading to dislocation penetration through the graphene or interfacial debonding, which significantly reduces the load transfer efficiency[25].

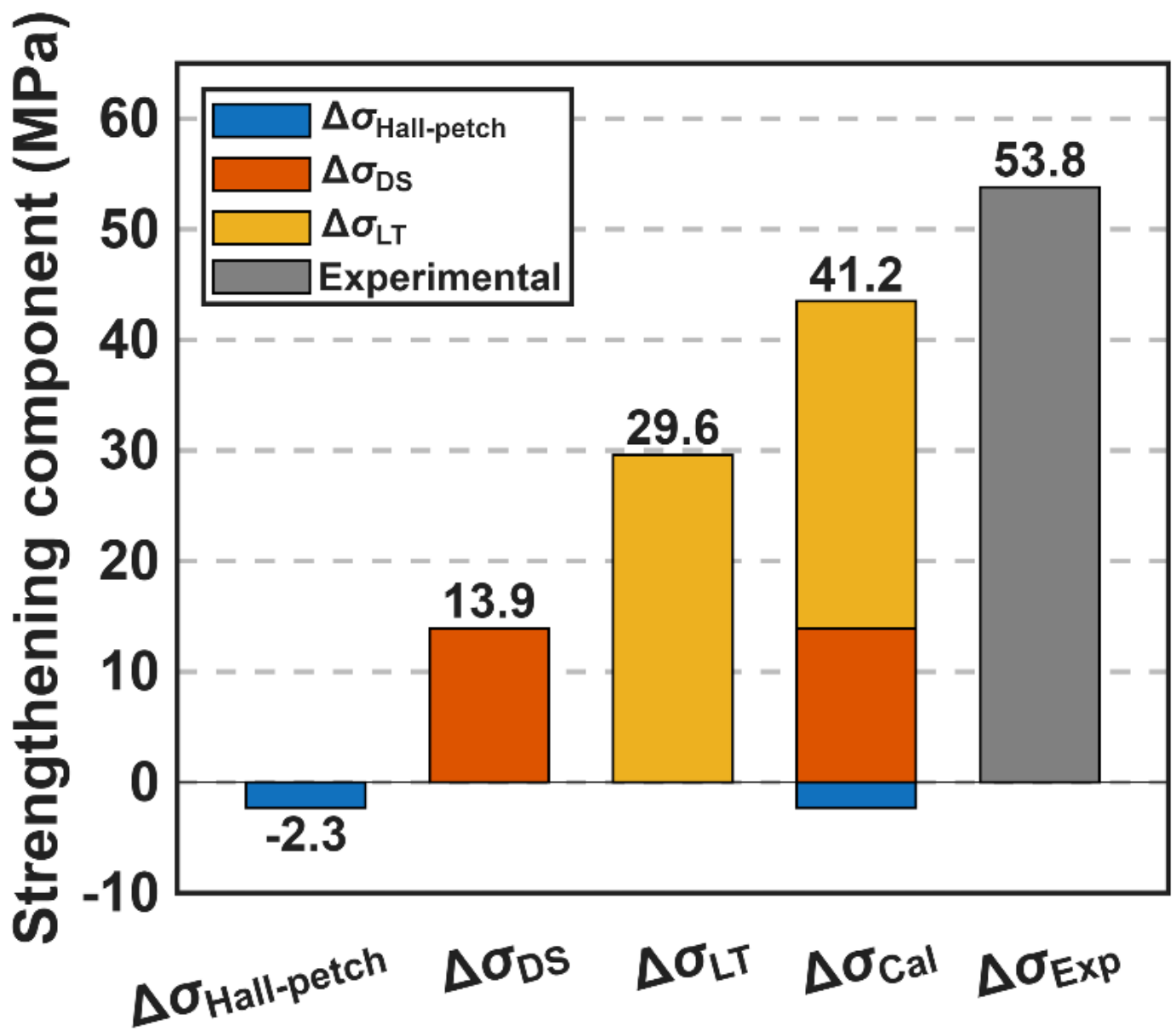

Supplementary Figure 17: Quantitative strengthening component calculation

Quantitative strengthening component calculation of the Ni/PMMA-Lattice-HP sample compared with the Ni-Lattice-HP sample, containing Hall-petch strengthening, dislocation strengthening, and load transfer strengthening.

Supplementary Note 9. Toughening mechanisms of as-fabricated Gr-Ni composites

9.1 Mesoscale extrinsic toughening dominated by the continuous 3D Gr network

Unlike discrete graphene nanosheets that provide only localized toughening, the 3D interconnected Gr network conformally wrapping the Ni scaffold enables global regulation of crack propagation throughout the bulk composite. When a crack propagates, the continuous 3D Gr network acts as a persistent barrier, forcing extensive crack deflection, branching, and twisting. This is directly evidenced by the highly tortuous crack path with abundant secondary and tertiary cracks on the fracture surface of the Ni/PMMA-Lattice-HP specimen. Such a tortuous path dramatically increases the total fracture length and the associated energy dissipation — a classic extrinsic toughening mechanism validated in Gr-MMCs[6,26]. More importantly, unlike discrete graphene flakes that induce only local deflection, the 3D Gr network suppresses the formation of catastrophic through-thickness cracks throughout the entire loading process, which is key to avoiding premature fracture and maintaining high ductility.

The robust semi-coherent Gr-Ni interface (**Supplementary Fig. 5d, e**) enables the continuous Gr network to form stable bridging ligaments in the crack wake, resisting crack opening and slowing propagation[27]. During final fracture, the as-grown graphene layers are gradually pulled out from the Ni matrix, as evidenced by abundant pulled-out and bent FLG fragments on the fracture surface (**Fig. 6a**). Notably, the 3D continuous network ensures that this crack-bridging and pull-out toughening effect operates across the entire fracture plane, not merely in local regions.

9.2 Micro scale intrinsic toughening for ductility retention

The interpenetrating 3D Gr-Ni architecture creates a heterogeneous microstructure consisting of graphene-reinforced regions and pure Ni matrix regions with a large difference in strength and deformability. During tensile deformation, the mechanical incompatibility between these regions generates long-range back stress in the matrix. This back stress not only contributes to strength enhancement but also promotes sustained dislocation accumulation and multiplication throughout the deformation process[28,29]. Direct evidence is the maintained high strain hardening rate of the Ni/PMMA-Lattice-HP specimen until fracture, which effectively suppresses premature necking and enables extensive uniform plastic deformation, thereby retaining the excellent ductility of the pure Ni matrix. Further support comes from EBSD results of fractured samples (**Fig. 7**): the distribution of low-angle grain boundaries (LAGBs) in the Ni/PMMA-Lattice-HP sample clearly divides the material into two regions with distinct deformation levels. Meanwhile, the Ni matrix region retains a high dislocation storage capacity, underpinning subsequent plastic deformation and energy absorption.

Therefore, the combination of global extrinsic toughening (crack deflection/branching, bridging, pull-out) and intrinsic toughening (heterogeneous deformation-induced back stress, sustained strain hardening) explains the exceptional toughness enhancement. The decoupling of strengthening and toughening — strength from high-quality graphene and its interface, toughness from the 3D architecture — represents a paradigm enabled uniquely by the proposed L-PBF-based in-situ synthesis strategy in this study.

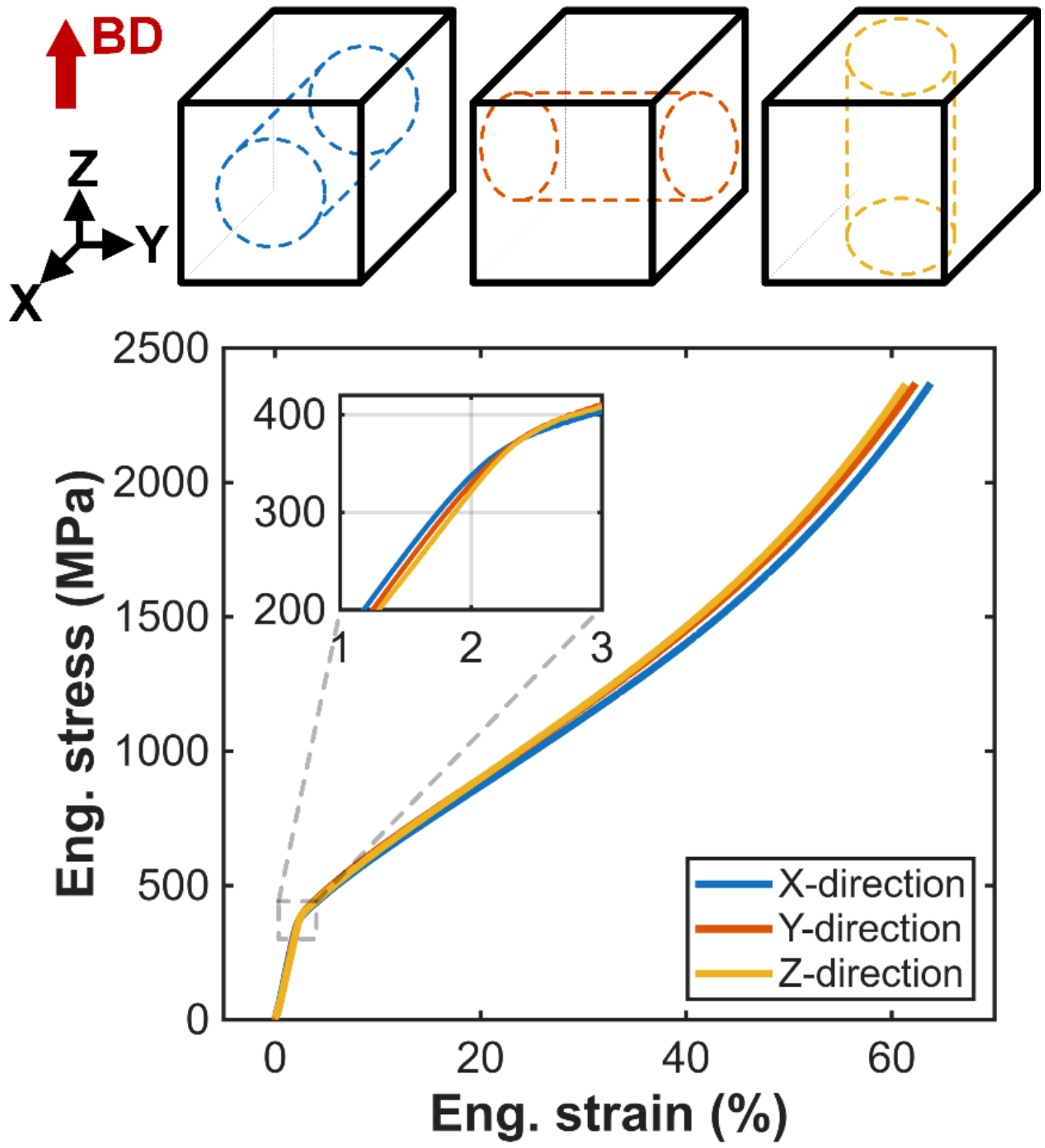

Supplementary Figure 18: Uniaxial compression tests of the as-fabricated Ni/PMMA-Bulk composite.

Compression tests are conducted along the building direction (BD), the Z direction, and its two orthogonal directions, X and Y. The compressive yield strengths for the X, Y, and Z directions are 379.83 MPa, 376.65 MPa, and 383.14 MPa, respectively, indicating isotropic behavior. Source data are provided as a Source Data file.

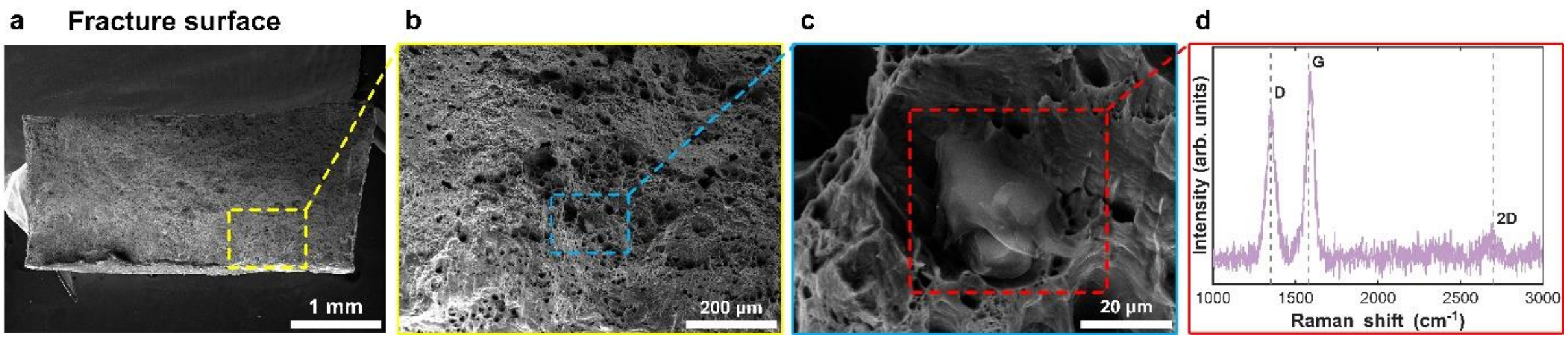

Supplementary Figure 19: Characterizations of the fracture surface of the Ni/PMMA-Lattice-HP composite.

a, b, c SEM images of the fracture surface of the Ni/PMMA-Lattice-HP composite. Scale bar, 1 mm (**a**), 200μm (**b**), 20 μm (**c**). **d** Raman spectrum of highlighted pulled-out Gr flake in (**c**). Source data are provided as a Source Data file.

Supplementary Table 1. Strengthening efficiency factors of Gr-MMCs with various architectures

Composites	$\sigma_{UTS,\ C}$ (MPa)	$\sigma_{UTS,\ M}$ (MPa)	f (vol.%)	R (%)	Retention (%)	Reference
Ni/PMMA-Lattice-HP	648	375	0.095	766.3	100.6	This work
Random 3DN-Gr-Ni	521	390	0.3	112	101.8	[30]
Random 3DN-Gr-Ni	487	433	0.5	24.9	96.5	[31]
Random 3DN-Gr-Cu	319	202	0.387	149.7	73	[32]
Random 3DN-Gr-Cu	276	211	0.5	61.6	89.2	[33]
Random 3DN-Gr-Cu	318	206	2	27.2	76.7	[34]
Random 3DN-Gr-Cu	339	234	1.15	39	79.7	[35]
Random 3DN-Gr-Cu	308	227	2	17.8	66.7	[36]
Random 3DN-Gr-Al	363	241	4.12	12.3	65.9	[37]
Laminated Gr-Cu	270	218	0.3	34.4	108.9	[38]
Laminated Gr-Cu	362	258	0.6	67.2	44.4	[39]
Laminated Gr-Cu	705	494	0.8	53.4	100	[40]
Laminated Gr-Al	260	201	0.75	39.1	107.1	[41]
Laminated Gr-Cu	305	218	1.6	24.9	94.3	[42]
Homogenous Gr-Cu	335	255	2.5	12.5	36.6	[43]
Homogenous Gr-Cu	214	190	0.5	25.3	65.7	[44]
Homogenous Gr-Cu	303	157	3	31	88.5	[45]
Homogenous Gr-Cu	401	366	0.5	19.1	87.6	[46]

Homogenous Gr-Cu	296	238	3.5	7	98.8	47

Supplementary references

1. Baraton, L. *et al.* On the mechanisms of precipitation of graphene on nickel thin films. *Europhys. Lett.* **96**, 46003 (2011).
2. Chen, R. & Xu, M. Kinetic and volatile products study of micron-sized PMMA waste pyrolysis using thermogravimetry and Fourier transform infrared analysis. *Waste Manag.* **113**, 51–61 (2020).
3. King, A. A. K. *et al.* A New Raman Metric for the Characterisation of Graphene oxide and its Derivatives. *Sci. Rep.* **6**, 19491 (2016).
4. Evolution of the Raman Spectrum with the Chemical Composition of Graphene Oxide. *ACS Publications* https://pubs.acs.org/doi/full/10.1021/acs.jpcc.7b06236 (2017).
5. Yazyev, O. V. Effect of Metal Elements in Catalytic Growth of Carbon Nanotubes. *Phys. Rev. Lett.* **100**, (2008).
6. Zhang, Y. *et al.* Bioinspired, graphene-enabled Ni composites with high strength and toughness. *Sci. Adv.* **5**, eaav5577 (2019).
7. Kim, J., Son, S., Choe, M. & Lee, Z. *In situ* TEM investigation of nickel catalytic graphitization. *Mater. Today Adv.* **22**, 100494 (2024).
8. Das, S., Lahiri, D., Lee, D.-Y., Agarwal, A. & Choi, W. Measurements of the adhesion energy of graphene to metallic substrates. *Carbon* **59**, 121–129 (2013).
9. Dahal, A. & Batzill, M. Graphene–nickel interfaces: a review. *Nanoscale* **6**, 2548–2562 (2014).
10. Qiu, C. & Metselaar, R. Solubility of carbon in liquid Al and stability of Al4C3. *J. Alloys Compd.* **216**, 55–60 (1994).
11. Fang, W., Hsu, A. L., Song, Y. & Kong, J. A review of large-area bilayer graphene synthesis by chemical vapor deposition. *Nanoscale* **7**, 20335–20351 (2015).

12. Zhang, G. *et al.* In situ synthesized 3D network structure TC4 metal matrix composites reinforced by the graphene via using laser additive manufacturing. *J. Mater. Res. Technol.* **39**, 4020–4032 (2025).

13. Holzmond, O. & Li, X. In situ real time defect detection of 3D printed parts. *Addit. Manuf.* **17**, 135–142 (2017).

14. Bartlett, J. L., Heim, F. M., Murty, Y. V. & Li, X. *In situ* defect detection in selective laser melting via full-field infrared thermography. *Addit. Manuf.* **24**, 595–605 (2018).

15. Bartlett, J. L., Croom, B. P., Burdick, J., Henkel, D. & Li, X. Revealing mechanisms of residual stress development in additive manufacturing via digital image correlation. *Addit. Manuf.* **22**, 1–12 (2018).

16. Bartlett, J. L. & Li, X. An overview of residual stresses in metal powder bed fusion. *Addit. Manuf.* **27**, 131–149 (2019).

17. Yin, J. *et al.* A finite element model of thermal evolution in laser micro sintering. *Int. J. Adv. Manuf. Technol.* **83**, 1847–1859 (2016).

18. Wei, Z., Ni, Z., Bi, K., Chen, M. & Chen, Y. In-plane lattice thermal conductivities of multilayer graphene films. *Carbon* **49**, 2653–2658 (2011).

19. Armstrong, R. W. Hall–Petch description of nanopolycrystalline Cu, Ni and Al strength levels and strain rate sensitivities. *Philos. Mag.* **96**, 3097–3108 (2016).

20. Arya, A. *et al.* Strength and microstructure evolution in nickel during large strain wire drawing. *Acta Mater.* **221**, 117396 (2021).

21. Lander, J. J., Kern, H. E. & Beach, A. L. Solubility and Diffusion Coefficient of Carbon in Nickel: Reaction Rates of Nickel-Carbon Alloys with Barium Oxide. *J. Appl. Phys.* **23**, 1305–1309 (1952).

22. Li, Z., Gu, H., Luo, K., Kong, C. & Yu, H. Achieving High Strength and Tensile Ductility in Pure Nickel by Cryorolling with Subsequent Low-Temperature Short-Time Annealing. *Engineering* **33**, 190–203

(2024).

23. Ryu, H. J., Cha, S. I. & Hong, S. H. Generalized shear-lag model for load transfer in SiC/Al metal-matrix composites. *J. Mater. Res.* **18**, 2851–2858 (2003).

24. Yang, Y. *et al.* Strengthening behaviour of continuous graphene network in metal matrix composites. *Carbon* **182**, 825–836 (2021).

25. Shuang, F. & Aifantis, K. E. Relating the strength of graphene/metal composites to the graphene orientation and position. *Scr. Mater.* **181**, 70–75 (2020).

26. Zhang, M. *et al.* 3D printed Mg-NiTi interpenetrating-phase composites with high strength, damping capacity, and energy absorption efficiency. *Sci. Adv.* **6**, eaba5581 (2020).

27. Ritchie, R. O. The conflicts between strength and toughness. *Nat. Mater.* **10**, 817–822 (2011).

28. Li, Z. *et al.* Enhanced Mechanical Properties of Graphene (Reduced Graphene Oxide)/Aluminum Composites with a Bioinspired Nanolaminated Structure. *Nano Lett.* **15**, 8077–8083 (2015).

29. Lin, Z. *et al.* Graphene-decorated bimodal pure metal with high strength and ductility. *Mater. Res. Lett.* **12**, 815–824 (2024).

30. Lin, Z. *et al.* Enhanced strength and toughness of Ni/graphene composite via three-dimensional graphene-like nanosheets network. *Compos. Part B Eng.* **253**, 110499 (2023).

31. Fu, K. *et al.* An approach for fabricating Ni@graphene reinforced nickel matrix composites with enhanced mechanical properties. *Mater. Sci. Eng. A* **715**, 108–116 (2018).

32. Zhang, X. *et al.* A powder-metallurgy-based strategy toward three-dimensional graphene-like network for reinforcing copper matrix composites. *Nat. Commun.* **11**, 2775 (2020).

33. Shi, L. *et al.* Achieving high strength and ductility in copper matrix composites with graphene network. *Mater. Sci. Eng. A* **828**, 142107 (2021).

34. Zhang, X. *et al.* Achieving high strength and high ductility in metal matrix composites reinforced with a discontinuous three-dimensional graphene-like network. *Nanoscale* **9**, 11929–11938 (2017).

35. Zhang, X. *et al. In-situ* space-confined synthesis of well-dispersed three-dimensional graphene/carbon nanotube hybrid reinforced copper nanocomposites with balanced strength and ductility. *Compos. Part Appl. Sci. Manuf.* **103**, 178–187 (2017).

36. Chen, Y. *et al.* Fabrication of three-dimensional graphene/Cu composite by in-situ CVD and its strengthening mechanism. *J. Alloys Compd.* **688**, 69–76 (2016).

37. Lin, X. *et al.* Gaining strength-ductility combination in Al matrix composites with in-situ synthesized three-dimensional nanocarbon network. *J. Alloys Compd.* **970**, 172542 (2024).

38. Xiong, D.-B. *et al.* Graphene-and-Copper Artificial Nacre Fabricated by a Preform Impregnation Process: Bioinspired Strategy for Strengthening-Toughening of Metal Matrix Composite. *ACS Nano* **9**, 6934–6943 (2015).

39. Li, Z. *et al.* Graphene quality dominated interface deformation behavior of graphene-metal composite: The defective is better. *Int. J. Plast.* **111**, 253–265 (2018).

40. Li, Z. *et al.* Regain Strain-Hardening in High-Strength Metals by Nanofiller Incorporation at Grain Boundaries. *Nano Lett.* **18**, 6255–6264 (2018).

41. Li, Z. *et al.* Enhanced Mechanical Properties of Graphene (Reduced Graphene Oxide)/Aluminum Composites with a Bioinspired Nanolaminated Structure. *Nano Lett.* **15**, 8077–8083 (2015).

42. Cao, M. *et al.* Aligning graphene in bulk copper: Nacre-inspired nanolaminated architecture coupled with in-situ processing for enhanced mechanical properties and high electrical conductivity. *Carbon* **117**, 65–74 (2017).

43. Hwang, J. *et al.* Enhanced Mechanical Properties of Graphene/Copper Nanocomposites Using a

Molecular-Level Mixing Process. *Adv. Mater.* **25**, 6724–6729 (2013).

44. Graphene defect engineering for optimizing the interface and mechanical properties of graphene/copper composites. *Carbon* **140**, 112–123 (2018).

45. Yang, M., Weng, L., Zhu, H., Fan, T. & Zhang, D. Simultaneously enhancing the strength, ductility and conductivity of copper matrix composites with graphene nanoribbons. *Carbon* **118**, 250–260 (2017).

46. Kim, W. J., Lee, T. J. & Han, S. H. Multi-layer graphene/copper composites: Preparation using high-ratio differential speed rolling, microstructure and mechanical properties. *Carbon* **69**, 55–65 (2014).

47. Chen, Y. *et al.* Fabrication of in-situ grown graphene reinforced Cu matrix composites. *Sci. Rep.* **6**, 19363 (2016).
