# Supplementary material for: In-situ architecturing of 3D graphene networks in metals via laser additive manufacturing for strength-toughness synergy
Source: Nat Commun. 2026 Aug 21;17:10004. doi: 10.1038/s41467-026-77015-z (PMC13590628; doi:10.1038/s41467-026-77015-z)
Supplement: Supplementary file 2 [file 41467_2026_77015_MOESM2_ESM.pdf]

nature portfolio

Peer Review File

In-situ Architecturing of 3D Graphene Networks in Metals via Laser Additive Manufacturing for Strength-Toughness Synergy

Corresponding Author: Dr Kaihao Zhang

This file contains all reviewer reports in order by version, followed by all author rebuttals in order by version.

Version 0:

Reviewer comments:

Reviewer #1

(Remarks to the Author)
The authors studied a three-dimensional graphene network within the metal matrix in which graphene precipitation growth in situ during the metal printing process. They demonstrated a Ni/polymer precursor system where few-layer graphene fully wrapped Ni scaffolds. The composite showed a strength–ductility synergy, achieved a 72% enhancement in ultimate tensile strength (648 MPa) without sacrificing ductility (54% elongation), alongside isotropic mechanical behavior. The results are important and of great interest. The discussion is of in-depth. The reviewer believes that this is a paper of very high quality. It is suggested that the paper be accepted after the following minor comments are taken care of. I will be happy to review the revised manuscript.
1. Graphene and it derives have been blended with Ni to form MMCs. Some literature papers reported graphene tended to form nickel carbides. Did PMMA wrapped nickel powders form nickel carbides. The authors need to discuss such aspects and compare the results with reference to following paper.
Bioinspired, graphene-enabled Ni composites with high strength and toughness, Science Advances, 5 (2019) eaav5577
2. PMMA has oxygen and hydrogen. Nickel powder surfaces also have oxygen layers on the surface. Did the oxygen react with nickel to form oxides? Was the graphene found in the composite graphene oxides? Where did the oxygen go during the processing? A previous paper reported graphene oxide formation in MMCS. The authors need to discuss such aspects with reference to the following paper.
Bio-inspired, Graphene/Al2O3 Doubly Reinforced Aluminum Composites with High Strength and Toughness, Nano Letters, 17 (2017) 6907-6915
3. When PMMA formed graphene, did graphene wrapped onto nickel particles? Such graphene interlayer may play an important role in bonding between nickel particles. Did the authors see curved graphene on nickel particles? The authors need to discuss such aspects with reference to the following paper.
Tailoring nanocomposite interfaces with graphene to achieve high strength and toughness, Science Advances, 6 (2020) eaba7016
4. Additive manufacturing often forms defects. Did graphene help reduce the formation of defects in laser additive manufacturing of such MMCs? Did the authors exam the defects with and without PMMA derived graphene? The authors need to discuss defect and the role of graphene in defect formation in additive manufacturing with reference to the following papers.
In situ real time defect detection of 3D printed parts, Additive Manufacturing, 17 (2017) 135-142
In situ defect detection in selective laser melting via full-field infrared thermography, Additive Manufacturing, 24 (2018) 595-605
Prediction of microstructural defects in additive manufacturing from powder bed quality using digital image correlation, Materials Science and Engineering A, 794 (2020) 140002
5. Additive manufacturing often has residual stress or distortion in additively fabricated parts. Did graphene help reduce residual stress or part distortion? Could graphene high conductivity help heat transfer and reduce stress? The authors need to discuss such aspects with reference to the following papers.
An overview of residual stresses in metal powder bed fusion. Additive Manufacturing, 27 (2019) 131-149
Revealing mechanisms of residual stress development in additive manufacturing via digital image correlation. Additive Manufacturing, 22 (2018) 1-12

Reviewer #2

(Remarks to the Author)
The authors have developed a transformative manufacturing strategy to prepare the graphene-reinforced Ni-matrix composite with high strengthening efficiency. The strengthening mechanisms of the composite have been discussed. The results are interesting, but they have not made a significant breakthrough in the mechanical properties of the Ni-matrix composites because their strength and ductility are in line with some literature. In addition to the lack of some key information, I do not believe that it is publishable in its current form. The following issues should be addressed.
1. Why the Ni is selected for the growth of graphene? Is this means other metals, e.g., Cu, Fe, Ti, Mg, Al, is not suitable for the transformative manufacturing.
2. The PMMA molecule coating on the Ni particle surface would be destroyed due to the laser ablation. It is incredible that the 3D graphene network with a high-quality can be formed although the ideal process window is investigated. More experimental characterizations should be carried out to prove the structural integrity of the in-situ grown graphene reinforcements.
3. The authors mentioned that the 3D graphene network has formed in the Ni matrix, but the Raman spectra and CT data has failed to show the microstructures of the 3D graphene. Some other advanced techniques are recommended for the determination of a 3D graphene network.
4. A hot-pressing process has been used after the laser additive manufacturing. Why the hot-pressing is applied? Is it because the sintering density of the composite is not high, or the mechanical property of the initial state of the composite is not good? In fact, the 3D graphene network would generate in one-step hot-pressing process. The combination of laser-sintering and hot-pressing makes a loss of the advantages of the laser-sintering. On the other hand, as is known, the hot-pressing may damage the structures of graphene and lead an anisotropic Ni matrix, which influence the mechanical property of the composite. Thus, the role of hot-pressing should be stated.
5. There is also less information about the interfacial structure analysis. The interfacial structures of a composite are important for its mechanical response. Thus, the interface of the graphene/Ni composites should be clarified.
6. The strain hardening curves of the composites should be provided since it is helpful to determine the strengthening and plastic behaviors of the composite.
7. The contribution of 3D graphene in the strength and plasticity of the composite should be analyzed quantitatively.
8. The strengthening and toughening mechanisms of the graphene reinforced Ni-matrix composite have to be discussed in-depth.

Version 1:

Reviewer comments:

Reviewer #1

(Remarks to the Author)
The revised manuscript is of higher quality. Publish as is.

Reviewer #2

(Remarks to the Author)
I have reviewed the revised manuscript and recommend its acceptance for publication. The authors have properly addressed all the previous comments, and the manuscript meets the journal's publication criteria.

Open Access This Peer Review File is licensed under a Creative Commons Attribution 4.0 International License, which permits use, sharing, adaptation, distribution and reproduction in any medium or format, as long as you give appropriate credit to the original author(s) and the source, provide a link to the Creative Commons license, and indicate if changes were made.
In cases where reviewers are anonymous, credit should be given to 'Anonymous Referee' and the source.
The images or other third party material in this Peer Review File are included in the article's Creative Commons license, unless indicated otherwise in a credit line to the material. If material is not included in the article's Creative Commons license and your intended use is not permitted by statutory regulation or exceeds the permitted use, you will need to obtain permission directly from the copyright holder.
To view a copy of this license, visit https://creativecommons.org/licenses/by/4.0/

Response to the reviewers

Ref: NCOMMS-25-100125

Title: In-situ Architecturing 3D Graphene Networks in Metals via Laser Additive Manufacturing for Strengthening-toughening Synergy

Journal: Nature Communications

Thanks for the reviewers' and the editor's valuable comments and suggestions. We revised the manuscript accordingly. In the following, these are our responses to the reviewers' and the editor's comments. All the changes are highlighted in red color in the revised manuscript.

Reviewer #1 (Remarks to the Author):

Comments: The authors studied a three-dimensional graphene network within the metal matrix in which graphene precipitation growth in situ during the metal printing process. They demonstrated a Ni/polymer precursor system where few-layer graphene fully wrapped Ni scaffolds. The composite showed a strength–ductility synergy, achieved a 72% enhancement in ultimate tensile strength (648 MPa) without sacrificing ductility (54% elongation), alongside isotropic mechanical behavior. The results are important and of great interest. The discussion is of in-depth. The reviewer believes that this is a paper of very high quality. It is suggested that the paper be accepted after the following minor comments are taken care of. I will be happy to review the revised manuscript.

Reply:

We sincerely thank the reviewer for the positive evaluation and insightful suggestions, which are of great importance for improving the completeness and rigor of our study. We have carefully addressed the comments raised by the reviewer and made corresponding revisions and supplements to the manuscript. The detailed revision notes and supplementary explanations for each comment have been provided in the point-by-point responses.

1. Graphene and it derives have been blended with Ni to form MMCs. Some literature papers reported graphene tended to form nickel carbides. Did PMMA wrapped nickel powders form nickel carbides. The authors need to discuss such aspects and compare the results with reference to following paper.

Bioinspired, graphene-enabled Ni composites with high strength and toughness, Science Advances, 5 (2019) eaav5577

Reply:

We sincerely thank the reviewer for this insightful comment regarding the potential formation of nickel carbides in the proposed Ni-Gr composite system, which is critical for clarifying the Ni-Gr interfacial structure. We have investigated the existence of Ni carbides in the Ni/PMMA-Lattice-HP sample via XRD, XPS, and HRTEM. The detailed response is elaborated below, following the logical progression of our experimental findings.

First-principles calculations indicate that monoatomic carbon on the Ni surface prefers to occupy subsurface octahedral interstitial sites rather than surface adsorption sites, and the chemical potential of subsurface carbon is significantly lower than that of surface carbon[1].The reference paper (Science Advances, 5, **2019**) mentioned in the comment indeed verified the carbide formation in Ni/Gr material systems under certain temperatures[2]. However, we performed XPS and XRD characterizations on the fully densified Ni/PMMA-Lattice-HP sample to detect potential Ni carbide phases (**Supplementary Fig. 5a-c**), and no characteristic peaks of nickel carbides were observed in the test results, for instance, diffraction peaks at 39.3°, 41.7°, and 58.6° in XRD, which is respectively related to the (110), (006), and (116) planes of Ni_3C structure (PDF#06-0697). The absence of nickel carbide peaks can be attributed to the high temperature during LPBF and subsequent hot-pressing treatment, where the metastable Ni carbides could decompose, and the released C atoms will diffuse onto the graphene layer[3]. Given that the carbon content in our composite is relatively low (0.095 vol.%), even if there are small amounts of Ni carbides, it is challenging to directly detect them using XPS or XRD. This result is consistent with other reported paper[4].

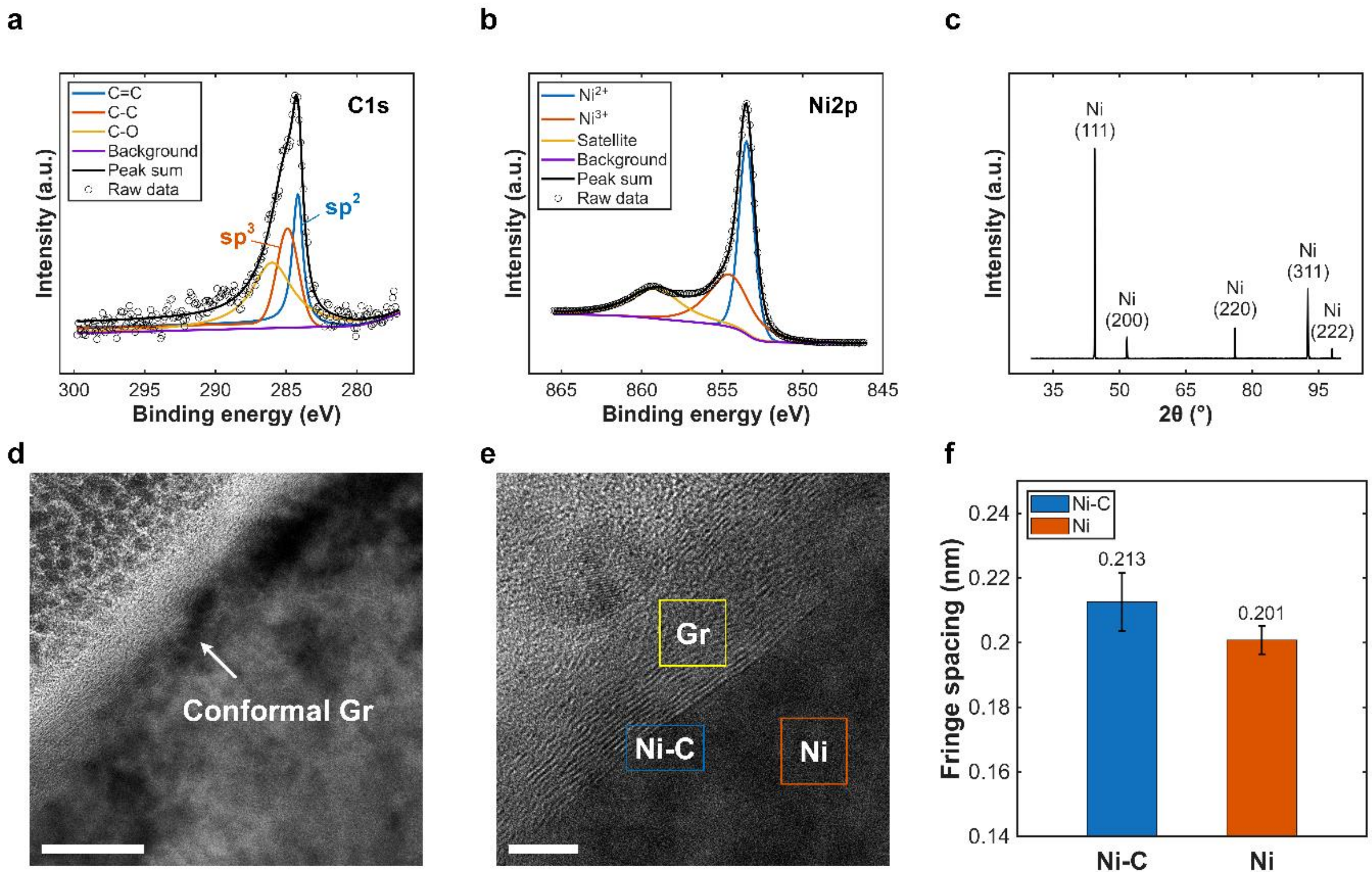

Supplementary Fig. 5: Gr-Ni interface characterization.

a, **b**, **c** XPS C1s, XPS Ni2p, and XRD spectrum of Ni/PMMA-Lattice-HP sample. **d**, **e** HRTEM images of Gr-Ni interface. **f** Fringe spacing comparison of Ni region and Ni-C region. Scale bar, 5 nm (**d**, **e**).

In order to better understand the microstructure between the as-grown graphene and underneath Ni matrix, where the Ni carbides are most likely to form, we implemented cross-sectional HRTEM characterizations of the Ni/PMMA-Bulk sample, as shown in **Supplementary Fig. 5d, e**. The HRTEM images clearly show an intimate, defect-free semi-coherent interface between the Ni matrix and the graphene layers. Notably, the lattice fringe spacing of the Ni-C region is measured to be slightly larger than the standard lattice spacing of the Ni region (**Supplementary Fig. 5f**). This measurable lattice expansion is evidence that carbon atoms dissolve into the Ni lattice, forming either carbides or solid solution.

In fact, graphene growth on metallic matrix including Ni, Pd, Co, etc., which exhibits high carbon solid solubility at elevated temperatures, follows the sequential process of solid dissolution, precipitation and crystallization[5]. Previous reports confirmed similar carbon dissolution phenomenon at the interface of the Pd-Gr system[6]. Notably, we did not blend graphene with Ni powders to form MMCs. Instead, based on the carbon diffusion mechanism, graphene was synchronously grown on the surface of the as-printed Ni matrix during the LPBF process.

Supplementary Fig. 5 has been added in Supplementary Information (SI). The discussion about Gr-Ni interface and formation of nickel carbides has been added in SI (Supplementary Note 4, Page 9, lines 93-119).

Supplementary Note 4. Characterization of Gr-Ni interface formed in LPBF process

First-principles calculations indicate that monoatomic carbon on the Ni surface prefers to occupy subsurface octahedral interstitial sites rather than surface adsorption sites, and the chemical potential of subsurface carbon is significantly lower than that of surface carbon[1]. This thermodynamic characteristic renders carbon highly prone to diffuse into the matrix during deposition on the Ni surface and eventually form a nickel carbide phase[1], which has been confirmed by Li's team[2].

In the XPS and XRD results of Ni/PMMA-Lattice-HP sample (**Supplementary Fig. 5a-c**), there are no characteristic peaks of Ni carbides observed, for example, diffraction peaks at 39.3°, 41.7°, and 58.6° in XRD, which is respectively related to the (110), (006), and (116) planes of Ni_3C structure (PDF#06-0697). The absence of nickel carbide peak can be attributed to the high temperature during LPBF and subsequent hot-pressing treatment, where the metastable Ni carbides could decompose, and the released carbon atoms will diffuse onto the graphene layer[3]. The ultra-low graphene content (0.095 vol.%) in the composite makes carbides undetectable by the employed characterization methods.

The cross-sectional HRTEM characterizations of the Ni/PMMA-Bulk sample helps to better understand the microstructure between the as-grown graphene and underneath Ni matrix, where the Ni carbides are most likely to form. The HRTEM images clearly show an intimate, defect-free semi-coherent interface between the Ni matrix and the graphene layers (**Supplementary Fig. 5d and e**). It is attributed to the high adhesion energy of graphene grown on Ni (72.7 J m^{-2}), which is around 6 times of that Gr-Cu interface[7]. Notably, the lattice fringe spacing of the Ni matrix exhibits a spacing of approximately 0.20 nm, corresponding to the (111) plane of face-centered cubic Ni[5]. While a distinct Ni-C transition region is present, where the lattice spacing gradually shifts from 0.20 nm to 0.23 nm (**Supplementary Fig. 5f**). This gradual change indicates partial diffusion and segregation of carbon atoms at the Ni subsurface during graphene growth, forming a Ni-C interfacial layer that ensures strong adhesion and efficient load transfer across the interface.

Corresponding modifications with suggested reference have also been updated in the main text (Page 13, lines 190-199).

It is noted that the initial layers of graphene grown on the surface of the Ni matrix typically do not exhibit measurable features in Raman spectra[8]. Cross-sectional high-resolution transmission electron microscopy (HRTEM) image confirmed the presence of FLG growth on the Ni surface (**Fig. 2i**). Notably, no obvious Ni carbide phase[2] was detected within the fabricated composites using X-ray photoelectron spectroscopy (XPS) and X-ray diffraction (XRD), as shown in **Supplementary Fig. 5a-c**. However, a slight lattice distortion of Ni is observed at the Gr-Ni interface, which is most likely induced by the infiltration of carbon atoms and provides direct evidence for the intimate Ni–Gr interface (**Supplementary Fig. 5d-f**, and **Supplementary Note 2**).

2. PMMA has oxygen and hydrogen. Nickel powder surfaces also have oxygen layers on the surface. Did the oxygen react with nickel to form oxides? Was the graphene found in the composite graphene oxides? Where did the oxygen go during the processing? A previous paper reported graphene oxide formation in MMCS. The authors need to discuss such aspects with reference to the following paper.

Bio-inspired, Graphene/Al2O3 Doubly Reinforced Aluminum Composites with High Strength and Toughness, Nano Letters, 17 (2017) 6907-6915

Reply:

We sincerely thank the reviewer for the critical question regarding the evolution of oxygen from PMMA decomposition, Ni surface oxide layers, the potential formation of Ni oxides or graphene oxide (GO), as well as the oxygen evolution during processing — these are key aspects to clarify the chemical stability and structural integrity of the in-situ grown graphene and the Ni matrix in the proposed composites. We have systematically characterized the oxygen evolution behavior during LPBF printing and hot pressing, verified the absence of Ni oxides and GO in the final composite via Raman spectral analyses (including **Raman D'_{inf} peak fitting**), and integrated the recommended reference and relevant literature to elaborate the underlying chemical mechanisms, as detailed below.

PMMA decomposes completely at temperatures above 500 °C during LPBF (**Supplementary Fig. 15**), and most native Ni oxide layer (mainly NiO) on Ni powder surfaces also undergo a reversible redox reaction under high temperature. Very few oxygen could remain in the final composite in the form of oxides, while most of the oxygen was released as gaseous byproducts during processing. (a) PMMA pyrolysis: under laser irradiation in LPBF printing (local temperature >1500 °C), PMMA undergoes unzipping decomposition and deoxygenation, releasing carbon atoms (quickly dissolved into the Ni melt pool) and oxygen-containing gaseous byproducts (CH_4O, CH_2O, etc.)[9,10]. Oxygen has extremely high electronegativity, and for most chemically stable polymers, it is almost impossible to produce O_2 during pyrolysis. (b) Reduction of Ni surface oxide: the molten Ni pool and carbon atoms from PMMA form a strong reducing environment at high temperatures. The native NiO on Ni powder surfaces can be rapidly reduced to metallic Ni via the reaction: $NiO+C \rightarrow Ni+CO\uparrow$.

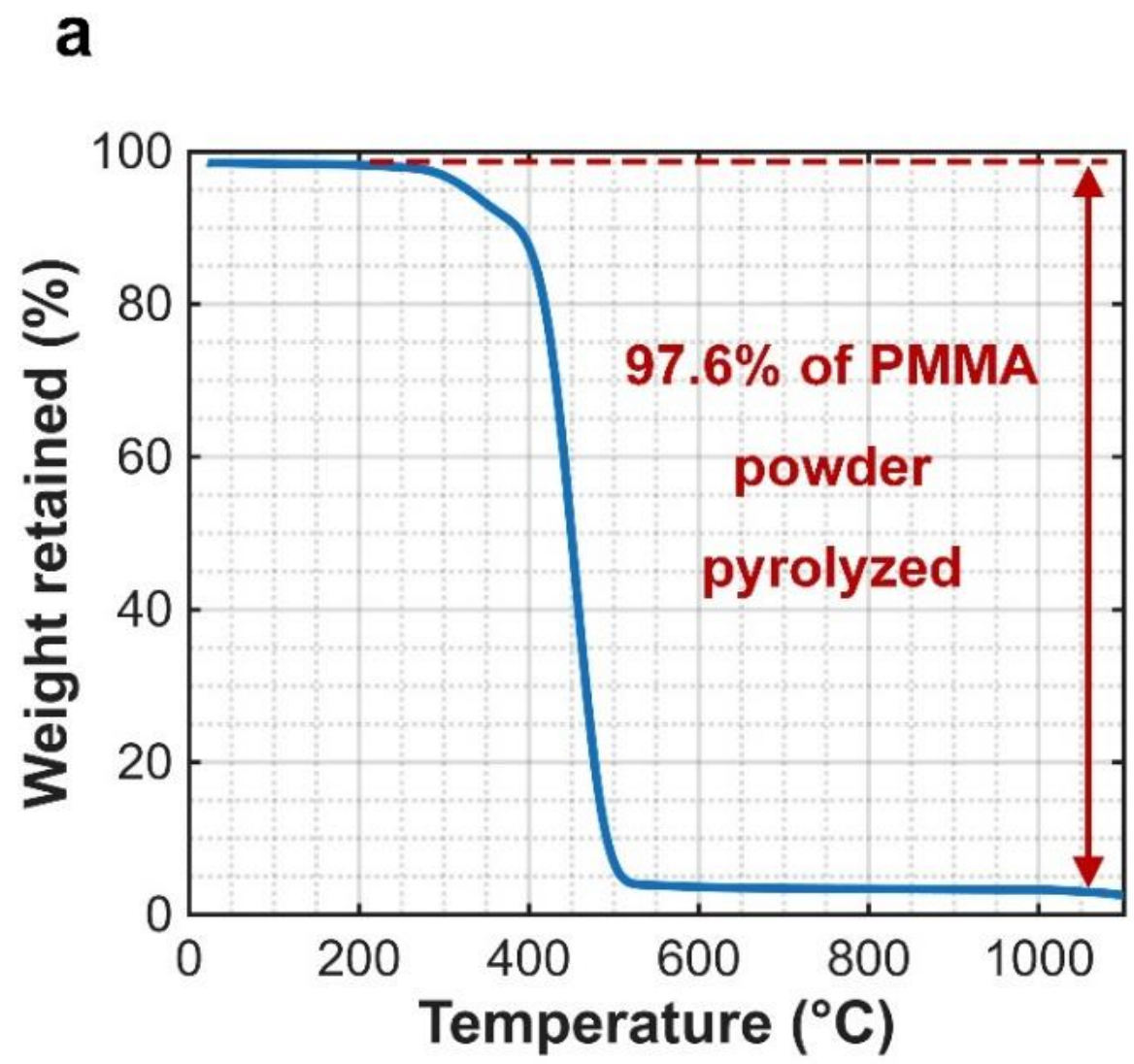

Supplementary Fig. 15a

a The TGA analysis showing 97.6% of PMMA powders can be fully pyrolyzed at temperatures over 500˚C.

To rigorously verify the absence of GO in the composite, we re-analyzed the Raman spectroscopy data with D peak derivative peak (D'_{inf}) fitting (532 nm laser excitation) following the methods in reported paper[11,12]. Raman spectra of the in-situ grown carbon phase were fitted with Lorentzian functions for the D (≈1350 cm^{-1}), G (≈1580 cm^{-1}), D'_{inf} (≈1620 cm^{-1}), and 2D (≈2700 cm^{-1}) peaks (**Supplementary Fig. 4**). The peak position difference between the D'_{inf} peak and the G band shoulder peak (G_{app}) is around 28 cm^{-1}, which is greater than 25 cm^{-1}. This is definitive spectral evidence of sp^2-hybridized pristine graphene, and distinct from GO (where $\omega_{D'inf}$ -ω_{Gapp}<25 cm^{-1} due to abundant oxygen-containing functional groups and sp^3 defects). The G peak of the in-situ grown carbon phase is sharp (FWHM = 38 cm^{-1}) and unshifted (≈1585 cm^{-1}), while GO typically shows a broadened and red-shifted G peak (1560–1570 cm^{-1}) due to oxygen functionalization[12,13].

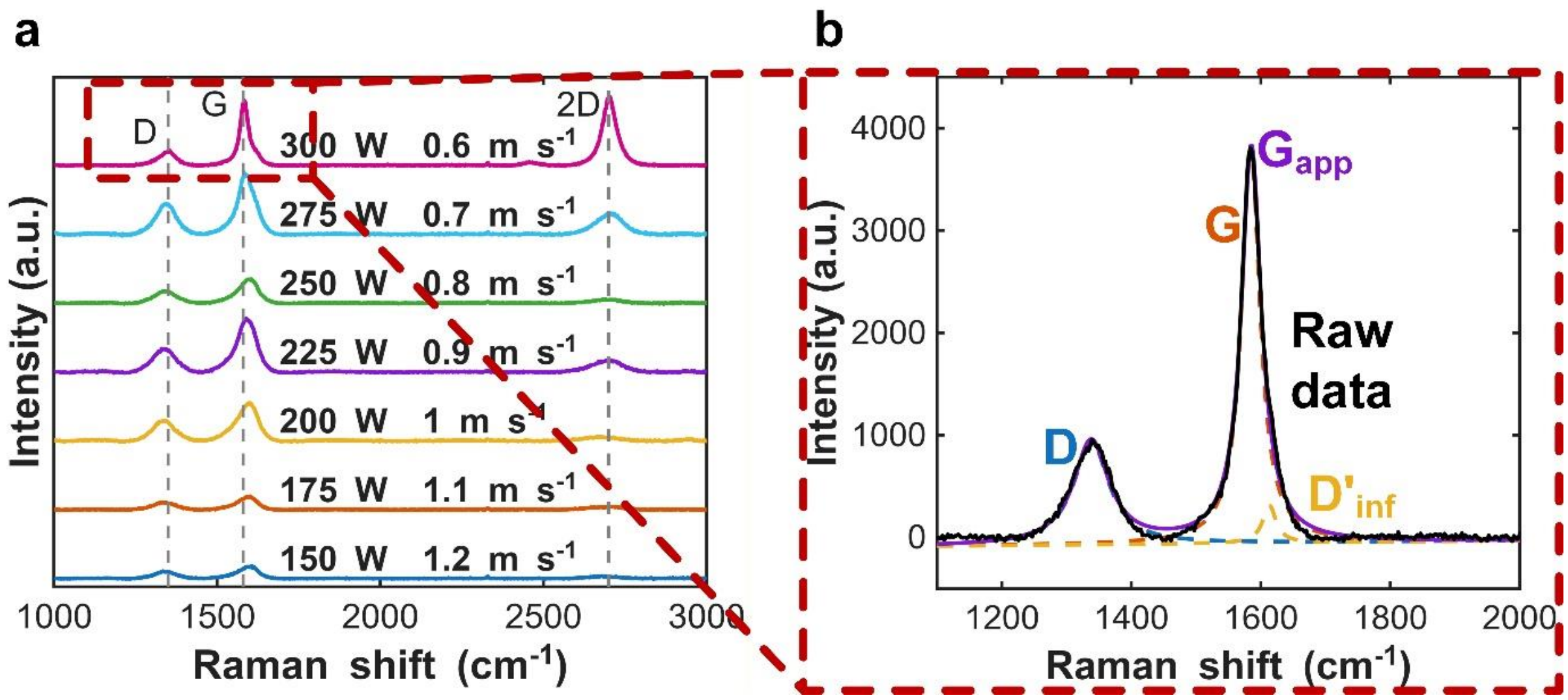

Supplementary Fig. 4: Identification of in-situ grown graphene and its derivatives.

a Typical Raman spectra of the printed samples with different laser parameters. **b** A fine fit of specific sample's Raman spectrum (with a power of 300 W and a scan speed of 0.6 m s^{-1})—incorporating the D, G, and D' peaks—indicates that the graphene is of high quality and rules out the possibility of GO.

It should be emphasized that although a C-O subpeak appears in the high-resolution C1s XPS spectrum (**Response Fig. 1a**), this does not indicate the presence of GO. No obvious subpeak corresponding to C=O is observed in the spectrum, suggesting that the C-O bonds most likely originate from organic contaminants or a small number of incompletely pyrolyzed PMMA particles in the material, as confirmed by the backscattered electron BSE SEM image of the fracture surface (**Response Fig. 1b**).

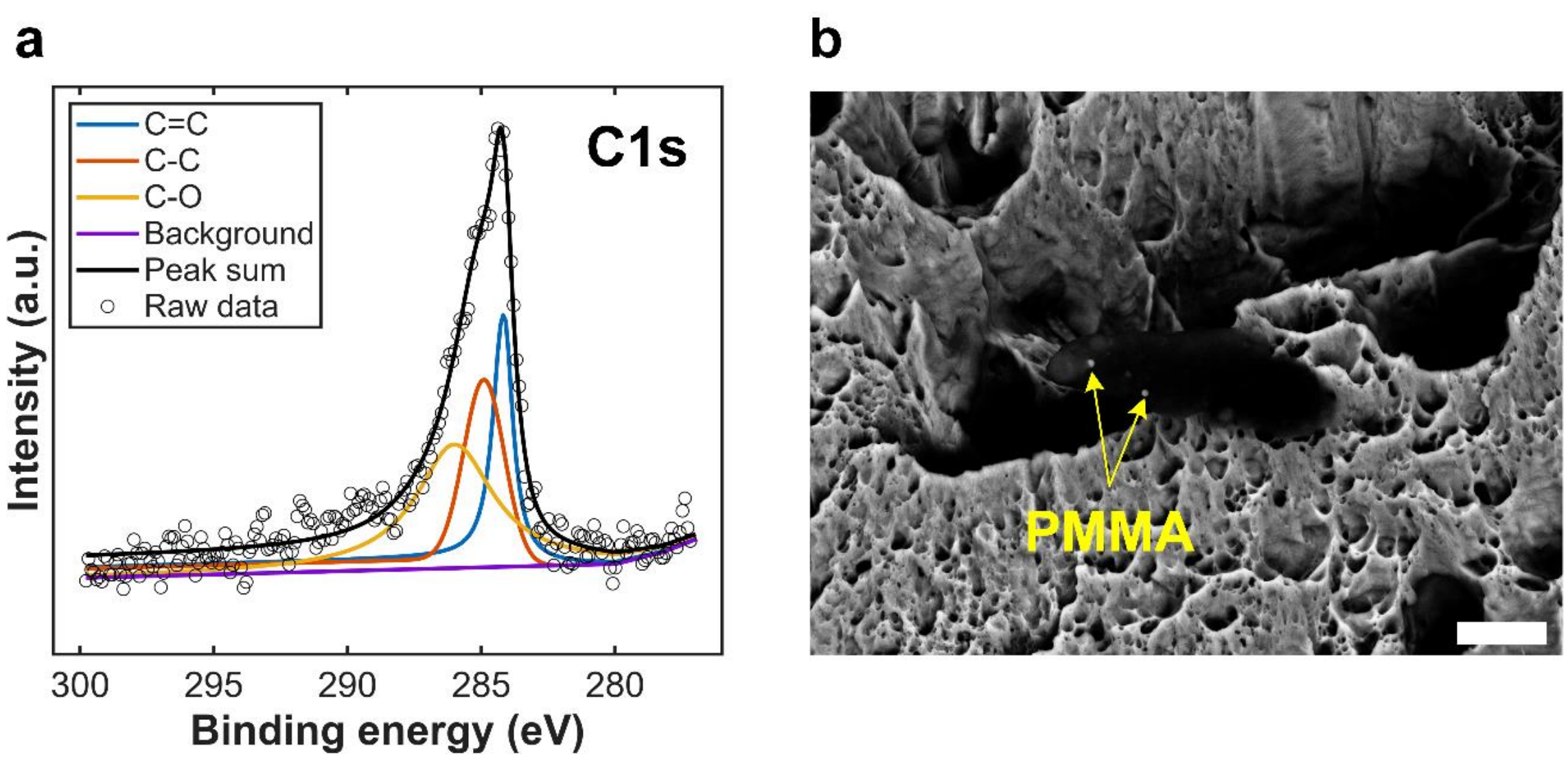

Response Fig. 1: Clarification of C-O bond source.

a XPS C1s spectrum of Ni/PMMA-Lattice-HP sample. **b** Fracture surface SEM image of Ni/PMMA-Lattice-HP sample. Scale bar, 10μm (**b**).

A clarification concerning graphene and its derivatives, along with **Supplementary Fig. 4** and relevant discussion (Supplementary Note 3), has been newly added to SI (page 9, lines 71-86):

Supplementary Note 3. Stoichiometric characterizations of as-grown FLG

The in-situ LPBF synthesis technique proposed in this study for Gr-Ni composites fabrication uses PMMA as the carbon precursor. PMMA decomposes almost completely into free carbon atoms and oxygen-containing gaseous byproducts (CH_4O, CH_2O, etc.) above 500 °C[9]. Continuous graphene layers can be formed through the dissolution, segregation, and crystallization of carbon atoms on the Ni melting/solidification track.

It has been reported in the literature that the peak separation between the D'_{inf} peak and the G peak in the Raman spectrum of graphene can serve as a criterion to distinguish between graphene oxide (GO), reduced graphene oxide (rGO), and pristine graphene[11]. Raman spectra of the in-situ grown carbon phase were fitted with Lorentzian functions for the D (≈ 1350 cm^{-1}), G (≈ 1580 cm^{-1}), D'_{inf} (≈ 1620 cm^{-1}), and 2D (≈ 2700 cm^{-1}) peaks (**Supplementary Fig. 4**). The peak position difference between the D'_{inf} peak and the G band shoulder peak (Gapp) is around 28 cm^{-1}, which is greater than 25 cm^{-1}. This is definitive spectral evidence of sp^2-hybridized pristine graphene. The G peak of the in-situ grown carbon phase is sharp (FWHM = 38 cm^{-1}) and unshifted (≈ 1585 cm^{-1}), while GO typically shows a broadened and red-shifted G peak (1560-1570 cm^{-1}) due to oxygen functionalization[14].

Corresponding modifications with suggested reference have also been updated in the main text (Pages 12-13, lines 187-190).

The sample printed at 300 W and 0.6 m/s exhibited the most prominent 2D peak ($I_{2D}/I_G \approx 1$), signifying the local formation of high-quality, few-layer graphene (FLG). In addition, the peak separation between the D' peak and the G peak in the Raman spectrum rules out the presence of graphene derivatives[11,15] (Supplementary Note 3 and Supplementary Fig. 4).

3. When PMMA formed graphene, did graphene wrapped onto nickel particles? Such graphene interlayer may play an important role in bonding between nickel particles. Did the authors see curved graphene on nickel particles? The authors need to discuss such aspects with reference to the following paper.

Tailoring nanocomposite interfaces with graphene to achieve high strength and toughness, Science Advances, 6 (2020) eaba7016

Reply:

We sincerely appreciate the reviewer's comments on the graphene growth process details and the morphology of as-grown graphene on the Ni matrix. This is a key aspect to clarify the unique in-situ graphene synthesis mechanism of the proposed strategy in this study, which is fundamentally different from the conventional graphene coating on metal powders. We elaborate the distinct graphene growth processes during LPBF printing (no pre-wrapping on Ni powder particles) in the revised manuscript.

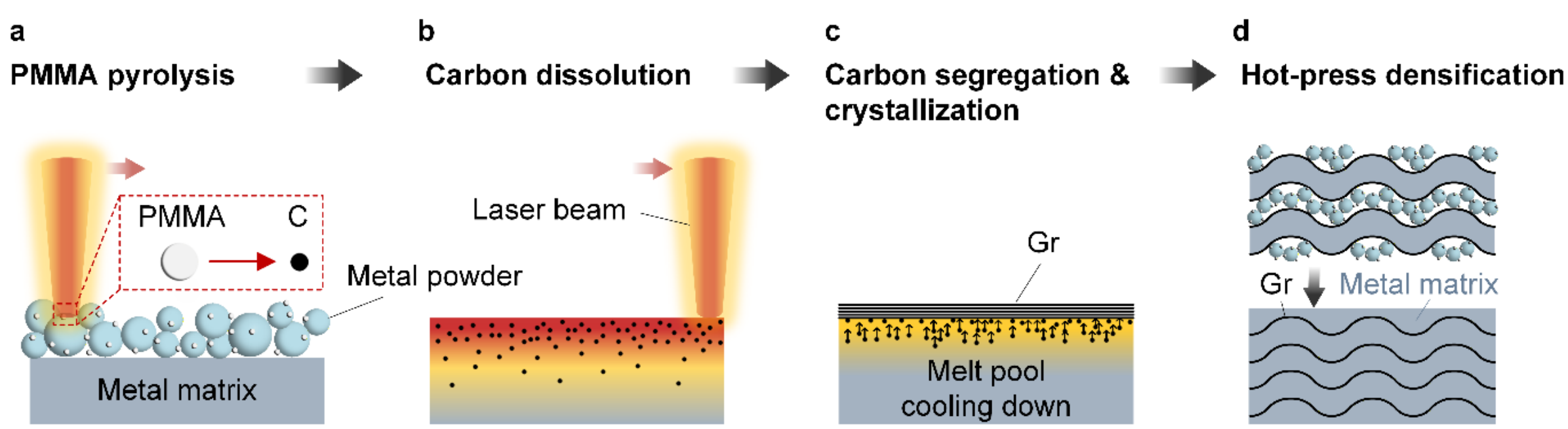

Supplementary Fig. 1: In-situ graphene growth mechanism during LPBF.

The in-situ LPBF fabrication of Gr-MMCs involves four primary procedures: **a** Metal powders are melted to form a molten pool, and PMMA particles are pyrolyzed into free carbon atoms; **b** Carbon atoms dissolve into the high-temperature molten pool; **c** During molten pool cooling, carbon atoms segregate to the surface and crystallize to form conformal graphene layers; **d** Gr-MMCs with designed spatial architectures are densified via hot pressing.

It is critical to clarify that our strategy does not involve pre-synthesizing or wrapping pre-existing graphene layers on Ni powder particles (the common powder metallurgy-based method for Gr-MMCs). Instead, we realize in-situ, conformal graphene growth on the dynamically formed Ni skeleton/scaffold

surface during LPBF printing, with the Ni/PMMA mixed powders only serving as the carbon and metal source. The detailed dynamic growth process is illustrated in **Supplementary Fig. 1**:

A) Laser-induced melting and PMMA pyrolysis: when the laser irradiates the Ni/PMMA mixed powder bed, Ni powders are selectively melted to form a molten pool, and the PMMA precursor particles undergo instantaneous pyrolysis and decomposition to release carbon atoms, which immediately dissolve into the molten Ni to form a carbon-Ni solution (**Supplementary Fig. 1a** and **b**).

B) In-situ precipitation on solidifying Ni surfaces: as the laser moves away, the molten pool rapidly solidifies into a continuous Ni scaffold (e.g. TPMS gyroid structure), and un-scanned Ni powders are intentionally encapsulated in the printed scaffold's pores. Driven by the exponentially decay in carbon solubility in Ni cladding during cooling, carbon atoms precipitate from the supersaturated Ni-C solid solution and segregate to the surface of the solidifying Ni scaffold, followed by sp^2 hybridization to form graphene layer (**Supplementary Fig. 1c**).

C) Post hot-pressing densification: the as-printed Ni scaffold (with in-situ grown graphene layers on the surface) and the unmolten Ni powders form a fully dense bulk composite via hot pressing, with the graphene layers preserving their 3D distribution following the printed gyroid scaffold in the Ni matrix as well as the conformal and intimate graphene-Ni interface (**Supplementary Fig. 1c**).

Supplementary Fig. 1 has been newly added to SI to elaborate on the fabrication of Gr-MMCs with tailored 3D configuration via in-situ LPBF.

4. Additive manufacturing often forms defects. Did graphene help reduce the formation of defects in laser additive manufacturing of such MMCs? Did the authors exam the defects with and without PMMA derived graphene? The authors need to discuss defect and the role of graphene in defect formation in additive manufacturing with reference to the following papers.

In situ real time defect detection of 3D printed parts, Additive Manufacturing, 17 (2017) 135-142

In situ defect detection in selective laser melting via full-field infrared thermography, Additive Manufacturing, 24 (2018) 595-605

Prediction of microstructural defects in additive manufacturing from powder bed quality using digital image correlation, Materials Science and Engineering A, 794 (2020) 140002

Reply:

We thank the reviewer for the insightful comment on the defect formation, which is a critical aspect for the practical applications of AM-fabricated Gr-MMCs. We have supplemented CT characterizations on the defect evolution with/without graphene growth in three kinds of samples mentioned in manuscript: (i) Ni/PMMA-Lattice-HP, (ii) Ni-Lattice-HP (graphene free), and (iii) Ni/PMMA-Bulk (fragmented in-situ grown graphene). **Supplementary Figure 15** compares the results. All samples tend to show a relatively low volume fraction of void defects, 0.1%, 0.1%, and 0.5%, respectively. We attributed this to the fact that an appropriate amount of graphene can regulate the thermodynamic behavior of the melt pool to a certain extent.

The original **Supplementary Fig. 6: (TGA and CT analysis)** has been renamed as **Supplementary Fig. 15: TGA and CT analysis of the fabricated Gr-Ni composites.** Meanwhile, CT characterization results of Ni-Lattice-HP and Ni/PMMA-Bulk samples have been newly supplemented in this figure.

This comment is highly relevant to Comment 5. Relevant revisions to the original manuscript discussion are comprehensively integrated into our response to Comment 5.

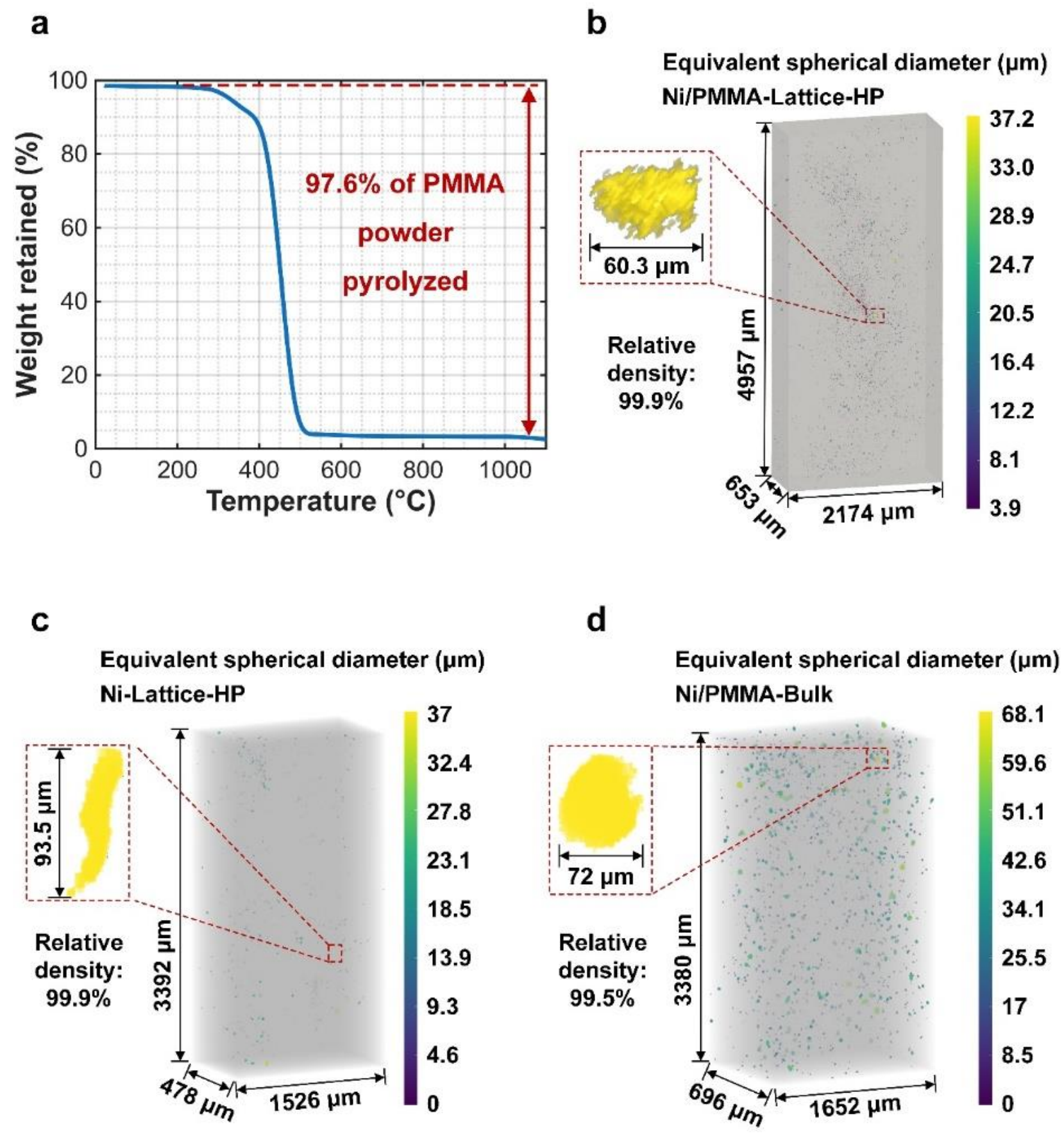

Supplementary Fig. 15: TGA and CT analysis of the fabricated Gr-Ni composites.

a The TGA analysis showing 97.6% of PMMA powders can be fully pyrolyzed at temperatures over 500˚C. **b**, **c**, **d** CT analysis of Ni/PMMA-Lattice-HP, Ni-Lattice-HP, and Ni/PMMA-Bulk samples showing 99.9%, 99.9%, and 99.5% relative density respectively. Pores are color-coded by equivalent spherical diameter.

5. Additive manufacturing often has residual stress or distortion in additively fabricated parts. Did graphene help reduce residual stress or part distortion? Could graphene high conductivity help heat transfer and reduce stress? The authors need to discuss such aspects with reference to the following papers.

An overview of residual stresses in metal powder bed fusion. Additive Manufacturing, 27 (2019) 131-149

Revealing mechanisms of residual stress development in additive manufacturing via digital image correlation. Additive Manufacturing, 22 (2018) 1-12

Reply:

We appreciate the reviewer for this insightful comment, which highlights the advantage of the in-situ grown graphene network within the metal matrix in a perspective of residual stress reduction for LPBF printed parts. This question is addressed as follows.

Response Table 1. Characterization results of electrical and thermal properties

	**Ni/PMMA-Lattice-HP**	**Ni-Lattice-HP**	**Ni/PMMA-Bulk (as-printed)**	**Ni/PMMA-Bulk (polished)**
Thermal diffusivity (mm^2 s^{-1})	14.23	16.22		
Electrical conductivity (S m^{-1})	0.85×10^7	1.21×10^7	1.3×10^7	0.89×10^7

Overall thermal diffusivity was measured for both the Ni/PMMA-Lattice-HP and the graphene-free Ni-Lattice-HP samples via laser flash analysis, and electrical conductivity was measured for Ni/PMMA-Lattice-HP, Ni-Lattice-HP, Ni/PMMA-Bulk (as-printed), and Ni/PMMA-Bulk (polished) samples via four-probe method. As shown in **Response Table 1**, the Ni/PMMA-Lattice-HP sample

containing the 3D graphene network exhibits slightly lower values in both properties than the Ni-Lattice-HP sample.

We attributed this reduction to the presence of the Ni–C transition region at the Gr–Ni interface. As noted in the response to Comment 1, the high carbon solubility of Ni at elevated temperatures inevitably leads to the formation of this interfacial layer, which has been reported to exhibit a high contact resistance — approximately twice the ideal contact resistance of pristine graphene[16]. Consequently, the macroscopic electrical and thermal transport properties are somewhat slightly degraded, which is consistent with observations in other Gr-MMC studies[17].

However, the electrical conductivity of the as-printed Ni/PMMA-Bulk sample is significantly enhanced (**Response Table 1**), which can be attributed to the highly conductive graphene layer on its surface. After polishing the sample (with the surface graphene layers removed), the electrical conductivity declines again, demonstrating the conductive enhancement effect of graphene. Similarly, it can be inferred that the in-situ grown graphene layer during printing process improves the local thermal conduction performance of the sample.

The continuous graphene layer, intimately bonded to the Ni matrix, facilitates more uniform heat dissipation during the rapid cooling process of LPBF. This could be verified by comparing the melt pool's and solidifying cladding's temperature field with Ni and Ni/PMMA powders via in-situ IR monitoring (**Supplementary Fig. 16**). The results indicate that the central region of the melt pool in the Ni/PMMA powder bed exhibits a faster cooling rate toward the surrounding areas compared with that in the Ni powder bed. This enables the rapid homogenization of the temperature field along the printing path and the reduction of local temperature gradients in the printed sample. As a result, residual stress accumulation and defect formation could be mitigated.

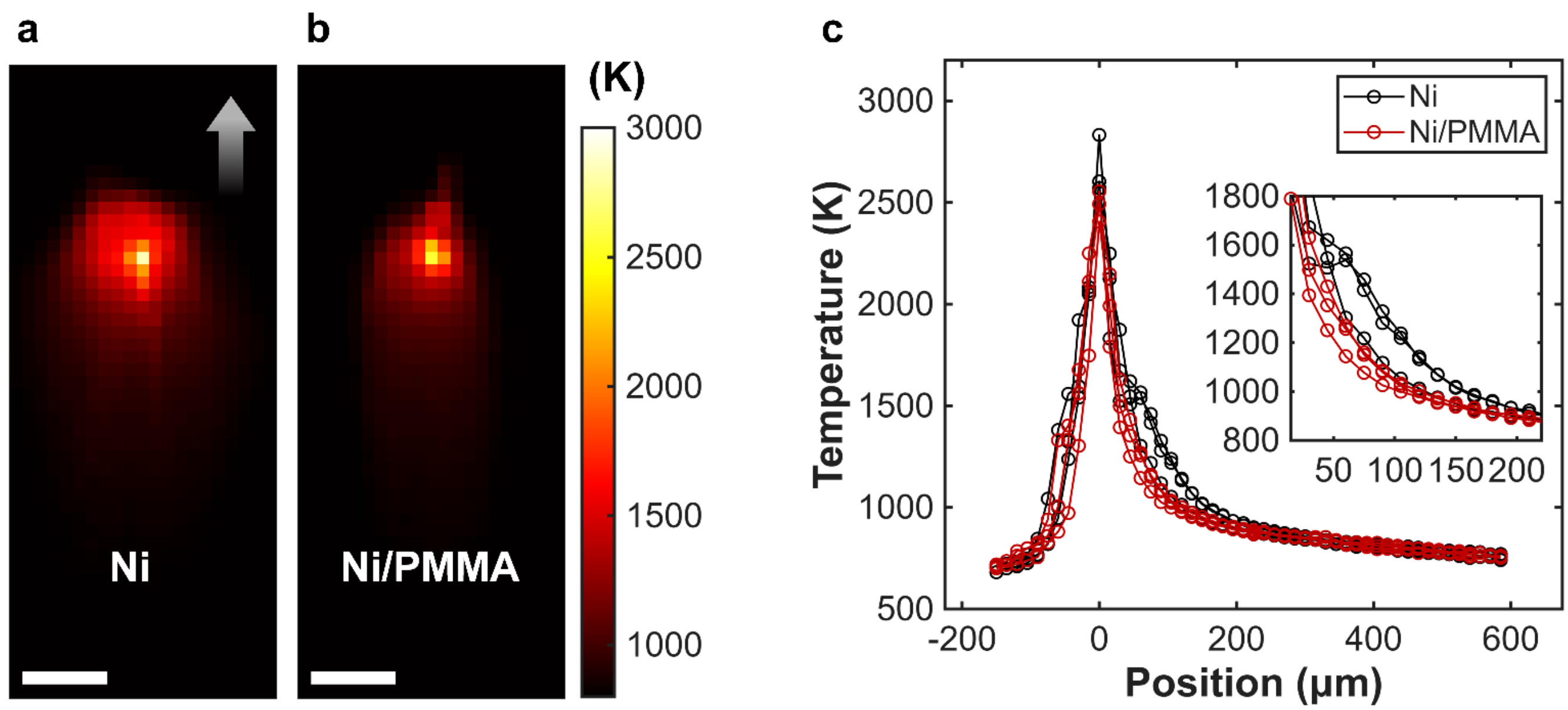

Supplementary Fig. 16: In-situ IR monitoring of Ni and Ni/PMMA melt pool.

a, **b** Reconstructed infrared (IR) images of melt pool of Ni and Ni/PMMA respectively. **c** Typical melt pool's and the solidifying cladding's temperature profile of Ni and Ni/PMMA. Scale bar, 100 μm (**a**, **b**).

Supplementary Figure 16 has been added in SI. We also added discussion to SI (Supplementary Note 7, pages21-22, lines 231-273), which is about the melt pool regulation mechanism of as-grown Gr.

Supplementary Note 7. Mechanism of in-situ grown FLG for microcrack suppression and thermal field regulation

Microcracks in LPBF-fabricated pure metals are primarily induced by uneven thermal stress during rapid heating/cooling and directional solidification[18, 19]. The in-situ grown FLG has an ultrahigh elastic modulus and excellent in-plane thermal conductivity (>3000 W m^{-1} K^{-1}), which could facilitate temperature uniformity in the Ni cladding during LPBF printing. Firstly, as shown in **Supplementary Fig. 13**, the existing graphene layer will be distorted and fragmented when the second Ni matrix layer is fabricated, and the graphene fragments embedded in the Ni melt pool could homogenize the local temperature field in the melt pool and the solidified cladding, reducing the thermal gradient and the resulting thermal stress. Second, the weak van der Waals interactions between FLG layers could allow for slight slippage under thermal stress, which dissipates the concentrated stress and prevents the initiation of microcracks and their propagation.

It has been confirmed that the extreme, non-uniform transient thermal gradient and the mismatched cooling and contraction between adjacent regions/layers during melting-solidification in laser AM, are the root causes of residual stresses and geometric distortion in LPBF AM parts[20, 21]. In fact, the in-plane thermal conductivity of graphene and its derivatives is significantly higher than that of bulk Ni, especially at high temperatures[22, 23], which can effectively improve the in-plane temperature field uniformity during LPBF AM cooling stage, thereby mitigating the in-plane thermal inhomogeneity induced by the laser scanning trajectories, and consequently reducing residual stress and property anisotropy.

To validate the above hypothesis, we employed high-speed IR thermal imaging to in-situ monitor the transient thermal behavior and melt pool thermal history of the pure Ni powder bed and the Ni/PMMA powder bed during the LPBF process, with the corresponding results presented in **Supplementary Fig. 16**. The reconstructed IR temperature field (**Supplementary Fig. 16a** and **b**) revealed evident differences in the spatial thermal distribution between the two systems. The pure Ni sample exhibited a relatively diffused high-temperature region with pronounced transverse thermal diffusion, leading to a broadened thermal affected zone. In comparison, the Ni/PMMA system featured a more confined high-temperature zone, where the temperature dropped more rapidly away from the melt pool center, indicating more efficient heat dissipation from the laser irradiation region. Quantitative analysis of the line-scan temperature profiles across the melt pool (**Supplementary Fig. 16c**) further highlighted these differences. The peak melt pool temperature of the pure Ni system reached approximately 2820 K, which was notably higher than that of the Ni/PMMA system (~2580 K), demonstrating that the introduction of the FLG precursor effectively mitigated local overheating during laser irradiation. More importantly, the temperature difference between the melt pool center and the surrounding solidified region was significantly reduced in the Ni/PMMA system. As highlighted in the magnified inset of the temperature profile, within the cooling region behind the melt pool, the Ni/PMMA sample exhibited a more uniform temperature evolution. These experimental observations directly confirm that the in-situ formed FLG can effectively homogenize the in-plane temperature field during the LPBF process, which alleviates the extreme transient thermal inhomogeneity induced by the localized laser heating and scanning trajectories. This provides solid experimental evidence supporting our proposed mechanism.

Corresponding modifications with suggested reference have also been updated in the main text (Page 20, lines 292-301).

Prior to mechanical testing, thermogravimetric analysis (TGA) was applied to the pre-mixed Ni/PMMA powders and confirmed about 97.6% decomposition of the PMMA precursor at temperatures over 500 ˚C, with only 2.4% residual carbon at 1100 °C (**Supplementary Fig. 15a**). Further, computed tomography (CT) scans revealed the density of the as-fabricated Ni/PMMA-Lattice-HP, Ni-Lattice-HP, Ni/PMMA-Bulk samples over 99.9%, 99.9%, and 99.5% respectively. All specimens were free of macro-defects, confirming that the proposed in-situ LPBF process does not compromise structural integrity (**Supplementary Fig. 15b-d**). This is partly attributed to the high thermal conductivity of in-situ grown graphene, which enables efficient homogenization of the melt pool temperature field[18,21] (**Supplementary Fig. 16** and **Supplementary Note 7**).

Reviewer #2 (Remarks to the Author):

The authors have developed a transformative manufacturing strategy to prepare the graphene-reinforced Ni-matrix composite with high strengthening efficiency. The strengthening mechanisms of the composite have been discussed. The results are interesting, but they have not made a significant breakthrough in the mechanical properties of the Ni-matrix composites because their strength and ductility are in line with some literature. In addition to the lack of some key information, I do not believe that it is publishable in its current form. The following issues should be addressed.

Reply:

We sincerely thank the reviewer for the constructive feedback. We highly appreciate the reviewer's recognition of the interest in the proposed manufacturing strategy and the discussion of the strengthening mechanisms, as well as the valuable feedback on the limitations of the original manuscript. It is acknowledged that while the reported mechanical property values — particularly the 72% strength increase without ductility loss — represent a significant synergistic enhancement compared to the LPBF printed Ni and many reported Gr-MMCs, we recognize that the primary novelty of this work lies not in the absolute property values alone but in the proposed transformative manufacturing paradigm. This approach uniquely integrates in-situ graphene synthesis with LPBF printing to achieve precise 3D architectural control of nano-reinforcements, directly addressing long-standing challenges (agglomeration, poor interfacial bonding, anisotropy, and lack of configurational design of such carbon nanomaterials as graphene in the metal matrix) that conventional methods cannot resolve. We have now clarified this distinction and supplemented the missing key information in the revised manuscript according to the reviewer's comments and suggestions.

1. Why the Ni is selected for the growth of graphene? Is this means other metals, e.g., Cu, Fe, Ti, Mg, Al, is not suitable for the transformative manufacturing.

Reply:

We sincerely thank the reviewer for this insightful comment, which is essential for clarifying the material selection rationale of this work and, more importantly, demonstrating the broad applicability of the

proposed transformative manufacturing strategy beyond the Ni model system. We have elaborated on the reasons for selecting Ni as the model material, validated the feasibility of this strategy in multiple typical engineering metal systems via systematic experiments, and discussed the graphene growth mechanisms on different metal matrices, as detailed below.

Ni was chosen as the model system to prove the concept of in-situ LPBF printing of graphene due to its high solid solubility for carbon (≈2.03 at.% which is over 50 times of that in Cu at 1000 ˚C[5]) and the resultant large chemical potential gradient of carbon adatoms on the surface and inside the bulk during rapid solidification — conditions that favor efficient carbon segregation and continuous graphene formation via the diffusion/precipitation mechanism. What should be noted is, Ni is not the only material compatible with the proposed manufacturing route. To demonstrate broader applicability, the same in-situ LPBF process was performed on four additional engineering metal systems using PMMA as the carbon precursor: **CuCrZr, 316L, AlSi10Mg, Ti6Al4V**. Raman characterizations of the as-printed sample surfaces confirmed the presence of characteristic graphene peaks (the D band ~1350 cm^{-1}, the G band ~1580 cm^{-1}, and the 2D band ~2700 cm^{-1}) in all these alloy systems, verifying the successful in-situ growth of graphene layers during LPBF printing. The detailed Raman spectra is shown in **Supplementary Fig. 6** below. Notably, the Mg system was not tested solely out of safety considerations for LPBF.

Besides, we would like to discuss the laser-induced graphene growth mechanisms on different metals. (a) For metals (e.g., Ni, 316L, etc.) having high carbon solubility at elevated temperatures and a significant decay in solubility during solidification/cooling. Graphene growth follows the dissolution-precipitation mechanism[24,25]. To be specific, carbon atoms released from PMMA pyrolysis upon laser irradiation first dissolve into the metal melt pool, then precipitate towards the solidifying metal surface driven by the solubility drop and chemical potential gradient and finally grow into graphene under the catalytic effect of the metal matrix.

For metals (e.g., CuCrZr, $AlSi_{10}Mg$, TC4, etc.) with relatively low carbon solubility in both molten and solid states, and graphene growth follows the surface-catalyzed growth mechanism[26,27], which is the surface reaction dominated process similar to the typical chemical vapor deposition growth of graphene on Cu foils. In the LPBF process, carbon atoms from PMMA decomposition are directly adsorbed and migrated on the surface of the molten/solidified metal, where carbon atoms undergo sp^2 hybridization to nucleate and grow into continuous graphene layers through the catalytic effect of the underlying metal. Our experiments further confirm that by optimizing the laser power and scan speed, we can tune the

temperature profile of the solidifying cladding, therefore, to control the crystallinity and quality of the formed graphene on these low carbon solubility metals.

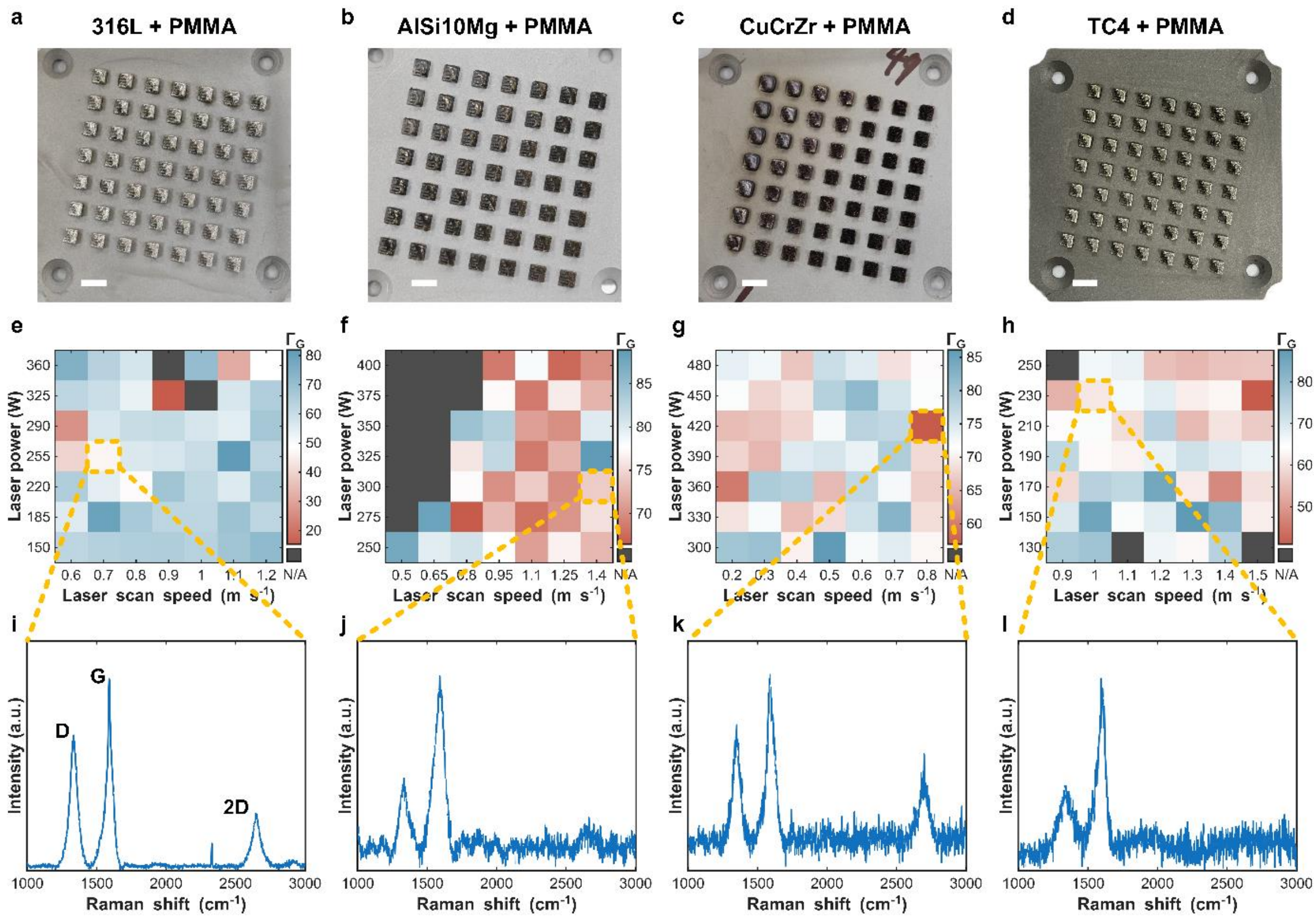

Supplementary Fig. 6: Raman results of as-printed Metal/PMMA-Bulk samples.

a, **b**, **c**, **d** Photos of the as-printed specimens of 316L/PMMA-Bulk, AlSi10Mg/PMMA-Bulk, CuCrZr/PMMA-Bulk, and TC4/PMMA-Bulk from the pre-blended metal/PMMA powder, respectively. Scale bar, 10 mm (**a**-**d**). **e**, **f**, **g**, **h** The Raman G peak's full width at half maximum (Γ_G) distribution corresponding to the 49 LPBF laser conditions in the printed samples in (**a**-**d**). **i**, **j**, **k**, **l** The representative Raman spectra in (**e**-**h**).

To deeply discuss our transformative technique for in-situ graphene growth on different metal matrix and the different graphene growth mechanisms, we added new discussion to SI (Supplementary Note 5, pages 11-12, lines 126-151).

Supplementary Note 5. Transformative in-situ LPBF strategy for various alloys

To verify the universality of the proposed in-situ graphene growth strategy during LPBF, we extended this transformative manufacturing route from the Ni matrix to another four widely used engineering alloys, including 316L austenitic stainless steel, AlSi10Mg aluminum alloy, CuCrZr copper alloy, and Ti-6Al-4V (TC4) titanium alloy. As shown in **Supplementary Fig. 6a**, high-integrity sample arrays of all four alloy systems were successfully fabricated via LPBF, demonstrating the process compatibility of our strategy with mainstream commercial metal AM materials. Raman spectroscopy analysis was performed (**Supplementary Fig. 6b** and **c**), illustrating the graphene growth quality as a function of laser power and scan speed, confirming that a well-defined process window for in-situ graphene growth can be identified for each alloy via tuning the melt pool thermal history. The characteristic Raman spectra for all four alloys exhibits the distinct D peak (~1350 cm^{-1}), G peak (~1580 cm^{-1}), and 2D peak (~2700 cm^{-1}) of graphene, providing unambiguous evidence for the successful in-situ synthesis of graphene on the printed surface of all investigated alloy systems via the same in-situ LPBF route.

The distinct subsurface carbon solubility[5] and carbon chemical potential[1] across various metallic matrices result in divergent growth mechanisms. The moderate carbon solubility in 316L leads to a graphene growth pattern similar to that of Ni, which follows the sequence of carbon dissolution, segregation, and crystallization. In contrast, AlSi10Mg and CuCrZr exhibit extremely low equilibrium carbon solubility in their matrices; bulk dissolution of carbon atoms remains negligible even at molten pool temperatures[5, 28]. For these two alloy systems, graphene growth proceeds via a surface adsorption and surface reaction mechanism[29]. For TC4, the exceptionally high chemical affinity between Ti and C readily induces the formation of brittle TiC precipitates in the matrix, which competes with the growth of surface graphene[30]. Therefore, precisely tuning laser parameters to promote the rapid segregation and nucleation of carbon atoms while inhibiting carbide precipitation is likely the key to controlling the quality of graphene growth on titanium alloy.

Corresponding modifications have also been updated in the main text (Page 13, lines 199-201).

Notably, the proposed in-situ LPBF printing technique is also applicable to various metallic materials beyond Ni (Supplementary Fig. 6 and Supplementary Note 5).

2. The PMMA molecule coating on the Ni particle surface would be destroyed due to the laser ablation. It is incredible that the 3D graphene network with a high-quality can be formed although the ideal process window is investigated. More experimental characterizations should be carried out to prove the structural integrity of the in-situ grown graphene reinforcements.

Reply:

We thank the reviewer for the insightful concerns regarding the structural integrity of in-situ grown graphene during LPBF printing. This is a core premise for validating the effectiveness of 3D printed graphene network reinforcement in the metal matrix. Below we elaborate the detailed processes with experimental validations of the continuous graphene layers growth on the solidified Ni surface during LPBF printing with the solid carbon precursor (e.g. PMMA).

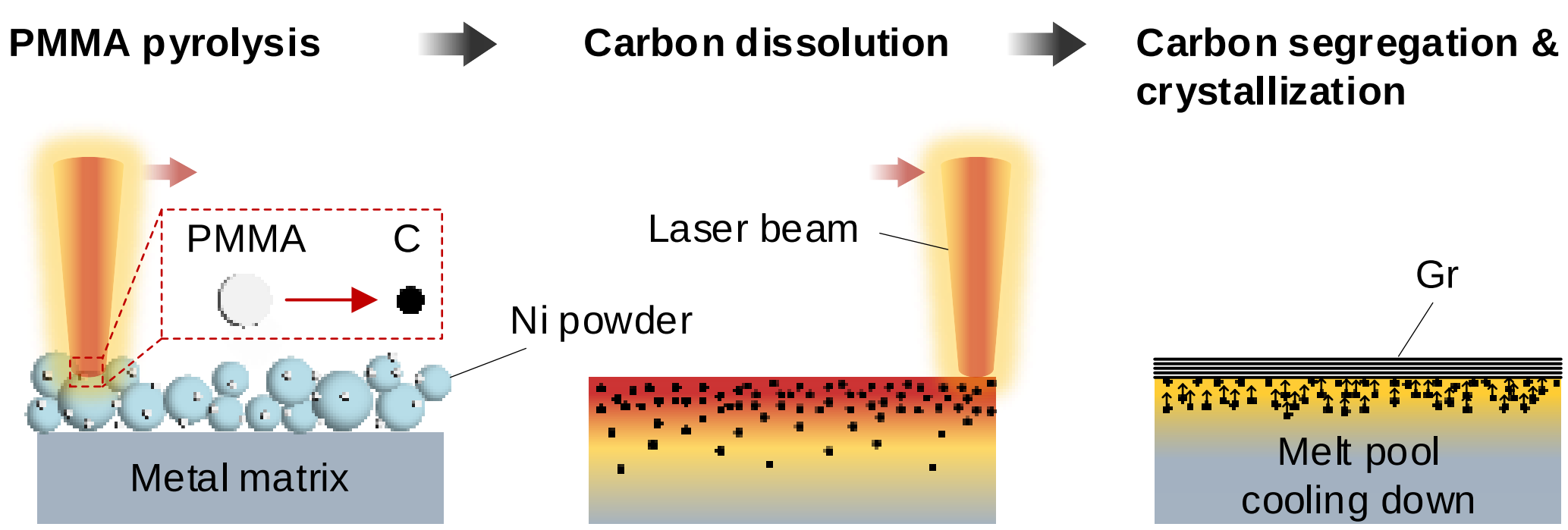

Response Fig. 2: Illustration of in-situ graphene growth mechanism during LPBF

First, a key clarification is necessary: the present strategy does not rely on a "molecular coating" of PMMA on Ni particles that would be directly ablated to form graphene coating on Ni surface. Instead, PMMA serves as a solid carbon precursor that undergoes complete pyrolysis under laser irradiation, releasing carbon atoms, together with Ni powders melting. Upon pyrolysis, PMMA chains decompose into MMA monomers, which rapidly fragment into small gaseous species (CO_2, CO, CH_4, etc.) and atomic carbon[10]. The released carbon atoms quickly dissolve into the Ni melt pool, as illustrated in **Response Fig. 2**, taking advantage of the high carbon solubility of Ni at elevated temperatures (~2.03 at.% at 1000 °C, which is about 50 times higher than that in Cu[5]). During the subsequent melt pool solidification and rapid cooling, the exponentially decay of carbon solubility and the strong chemical potential gradient drive carbon atoms to precipitate from the supersaturated solid solution in bulk Ni and segregate to the surface of the solidifying Ni cladding (**Response Fig. 2**), where sp^2 hybridization leads to graphene formation before the

as-solidified Ni completely cools down. The typical temperature for graphene growth on carbon-saturated Ni matrix could be as low as around 700 K[5,31,32]. Experimental observations (IR monitoring, **Supplementary Fig. 16**) and simulation results (**Supplementary Fig. 2**) jointly verified that the cooling duration of the printing track from the Ni melting point (~1728 K) to 700 K is approximately 1 ms. On the basis of this time period, the diffusion length L of carbon atoms is estimated to be about 41 nm ($L = 2\sqrt{D_T \tau}$, where $D_T = D_0 \exp(E_D/kT)$ and the temperature T is taken as 1000 °C)[24]. This value greatly exceeds the Ni subsurface depth (the carbon chemical potential in the subsurface of metals including Ni and Pd is lower than that on the surface[1]), which demonstrates that carbon atoms have adequate time to segregate to the surface and crystallize during the cooling process of LPBF printing. This mechanism is further supported by experiments using alternative polymers as carbon precursors: Ni/Polypropylene (PP)-Bulk and Ni/Polyethylene (PE)-Bulk samples printed under identical LPBF conditions also exhibited characteristic graphene Raman signals on their surfaces. In this regard, precise polymer stoichiometry is not critical; the essential role of the polymer precursor is to provide a carbon source upon laser pyrolysis. It is observed that Ni/PE-Bulk and Ni/PP-Bulk samples exhibit strong Raman D peaks and weak 2D peaks. This is mainly attributed to the excessively high viscosity of Ni/PE and Ni/PP mixed powders, which causes severe difficulties in powder spreading and poor printing quality, thereby deteriorating the growth quality of graphene on the printed surface.

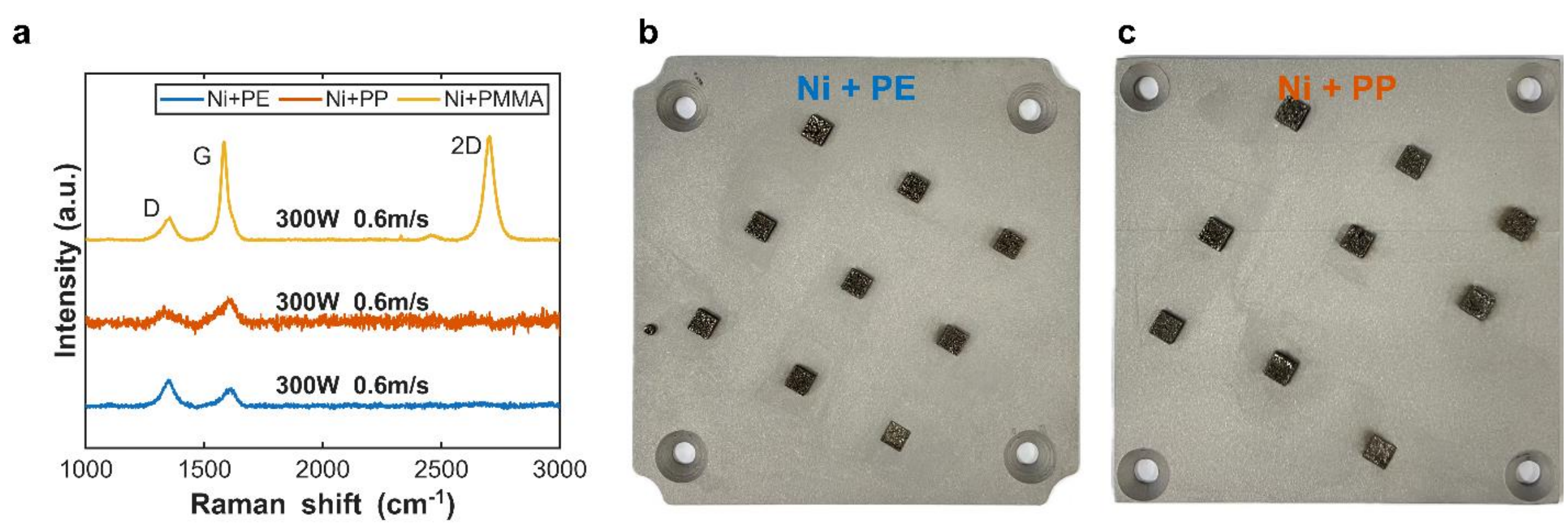

Response Fig. 3: Analysis of LPBF-printed Ni-Gr composites using different carbon precursor powders

a Raman spectra of the printed samples using three different carbon precursor powder, including PMMA, PP, and PE. **b c** Sample images of as-printed Ni/PE-Bulk and Ni/PP-Bulk respectively.

Second, the quality and coverage of the in-situ grown graphene are primarily governed by the melt pool cooling rate, which can be controlled by laser parameters (e.g., laser power, scan speed, etc.). The optimal parameter set identified in the manuscript yields continuous graphene layers with high crystallinity. To further substantiate this, additional characterizations were conducted. Raman mapping including $I_G, I_D/I_G, \Gamma_G$ over an area of 1 mm × 1 mm on the top surface of the printed Ni/PMMA-Bulk sample confirms over 98% coverage. Moreover, Raman mapping on all four lateral surfaces of the same sample similarly demonstrates high coverage and quality, as shown in **Supplementary Fig. 7**. These results collectively demonstrate that the in-situ synthesis strategy proposed in this study produces large-area, continuous, high-quality graphene layers on the printed metal surfaces.

Third, direct morphological evidence is provided by more SEM images shown in **Supplementary Fig. 8**. The top surface of the Ni/PMMA-Bulk sample exhibits wide-area, continuously distributed wrinkles along with occasional fragmented features — distinct signatures of graphene growth on Ni. This observation complements the Raman data and unequivocally confirms graphene formation during the LPBF process.

Finally, to preserve a specific 3D graphene configuration and integrity within the metal matrix, a gyroid-shaped Ni scaffold was fabricated via LPBF printing with Ni/PMMA pre-mixed powders. Following the graphene in-situ growth process on as-printing Ni cladding we evidenced above, graphene layers can directly form on the outer surface of the printed gyroid Ni scaffold. Notably, a gyroid structure exhibits an infinitely connected triply periodic minimal surface which is curved everywhere. As shown in **Supplementary Fig. 9**, detailed Raman characterizations on the curved surfaces of the printed scaffold confirm full coverage and conformal graphene growth, thereby validating the architectural integrity of the 3D network via LPBF printing.

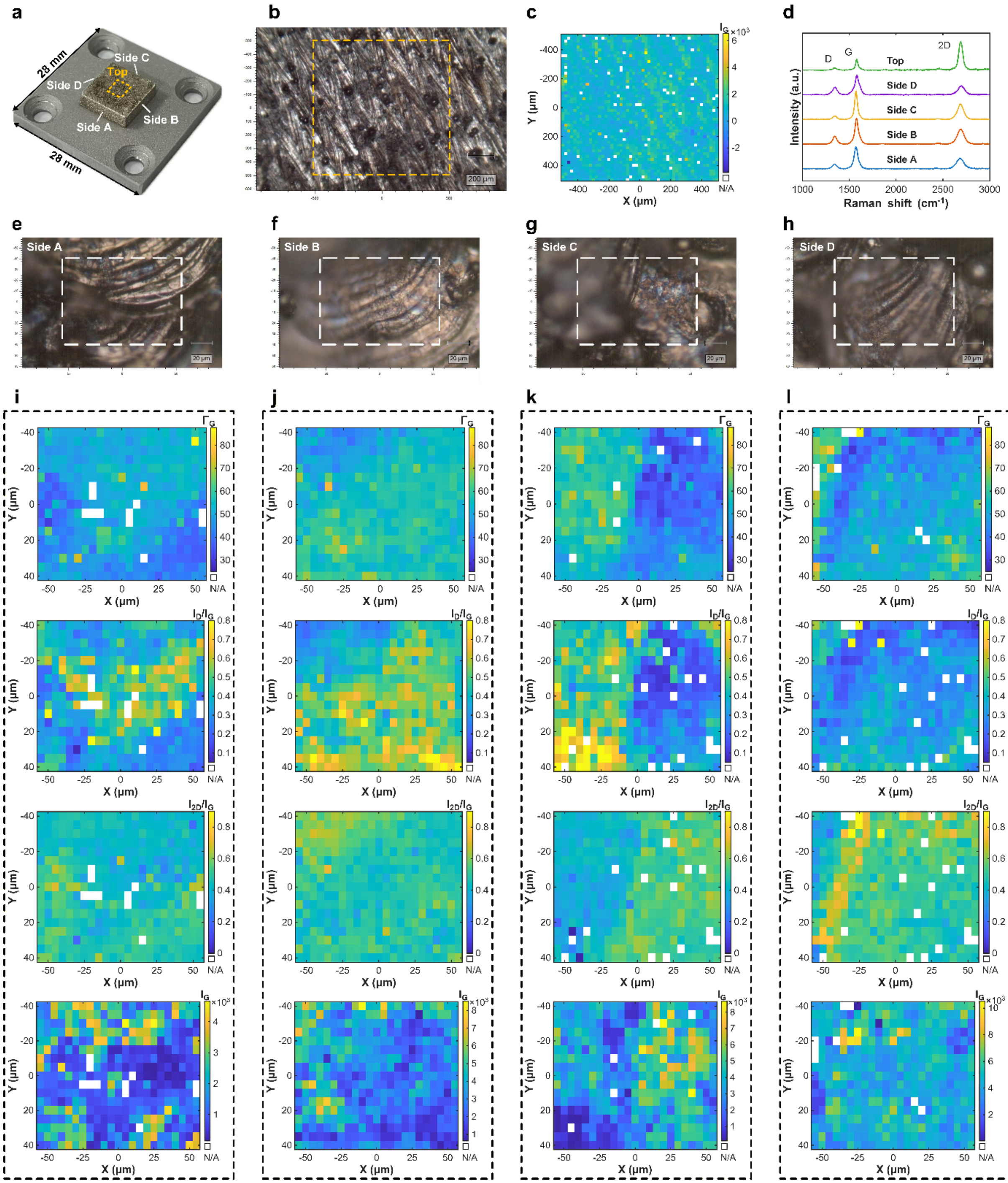

Supplementary Fig. 7: Raman results for the top and four lateral surfaces of the Ni/PMMA-Bulk sample.

a As-printed Ni/PMMA-Bulk sample photo. **b** OM of Ni/PMMA-Bulk sample's top surface. **c** Raman G peak intensity map of the dashed square in **(b)**. **d** Representative Raman spectra of Ni/PMMA-Bulk

sample's top surface and 4 lateral surfaces. **e**, **f**, **g**, **h** OM of Ni/PMMA-Bulk sample's 4 lateral surfaces. **i**, **j**, **k**, **l** Raman mapping results of Ni/PMMA-Bulk sample's 4 lateral surfaces.

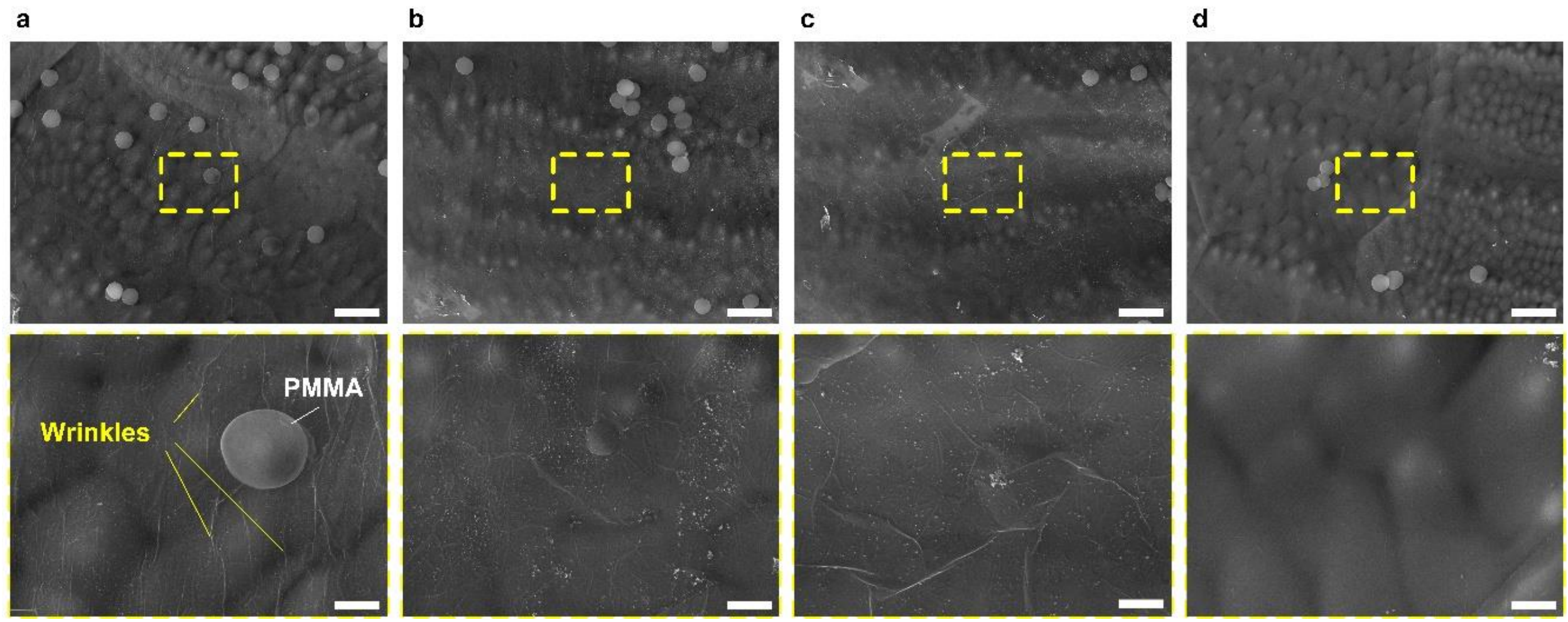

Supplementary Fig. 8: SEM images of the grown FLG on the printedNi.

a, **b**, **c**, **d** SEM images of Ni/PMMA-Bulk samples' top surface. The yellow lines in (**a**) point to the wrinkled microstructures of the graphene on the printed surface, while the white lines point to incompletely pyrolyzed PMMA particle. Scale bar, upper 5 µm, lower 1 µm (**a**, **b**, **c**, **d**).

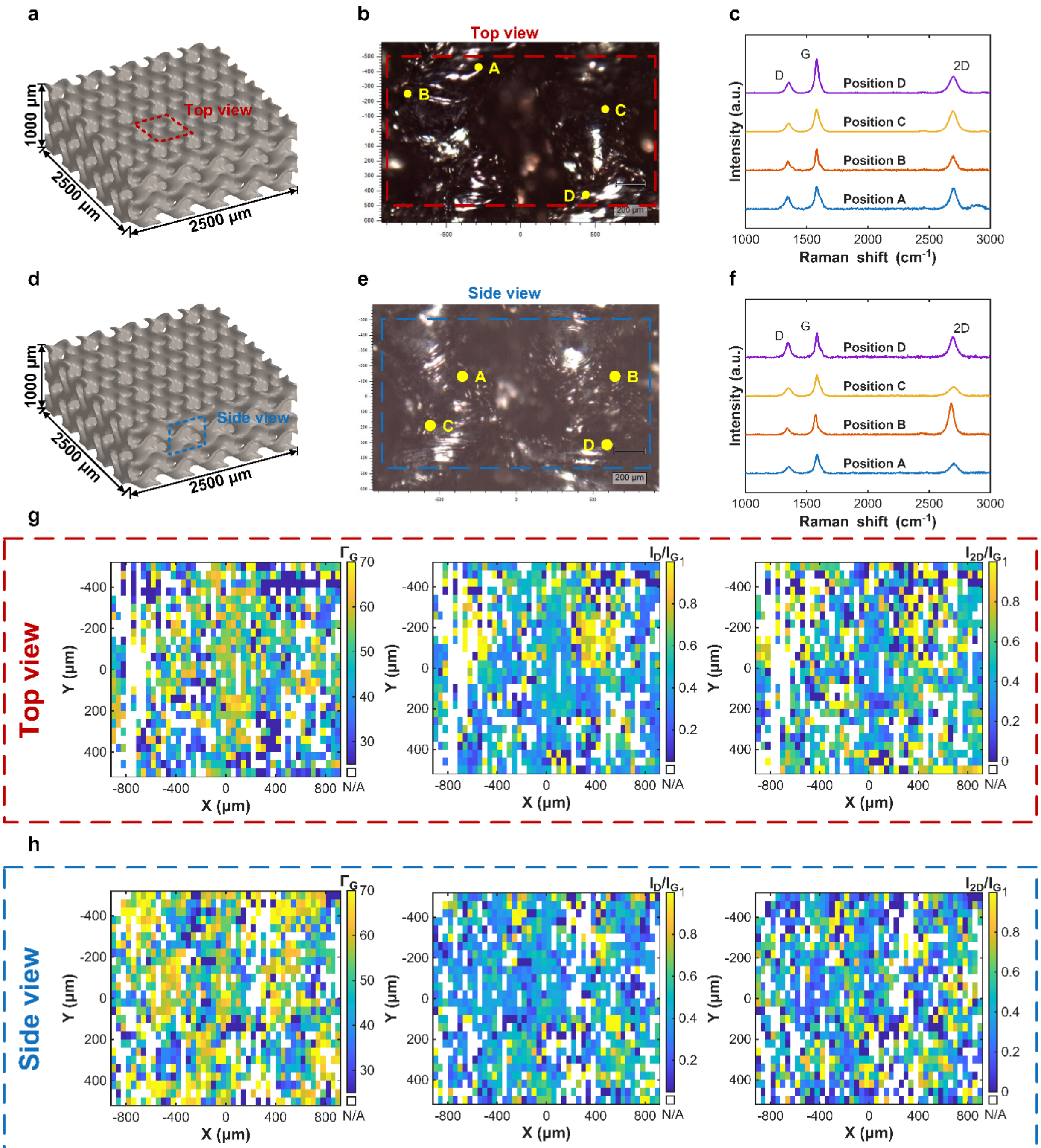

Supplementary Fig. 9: Characterizations of FLG grown on the curved surface of a printed gyroid Ni structure.

a, **d** Gyroid structure 3D model. **b**, **e** OM of printed gyroid Ni-Gr composite's top surface and lateral surface. **c**, **f** Representative Raman spectra of different position in (**b**) and (**e**), respectively. **g**, **h** Corresponding Raman results in (**b**, **e**).

These additional experiments and clarifications provides more comprehensive evidence for the structural integrity and quality controllability of the in-situ grown graphene reinforcement.

Newly supplemented figures have been added to SI, including **Supplementary Fig. 7**, **Supplementary Fig. 8**, and **Supplementary Fig. 9**.

Corresponding figures are also mentioned in main text (pages 13-14, lines 202-218).

To evaluate the coverage and conformality of the in-situ grown graphene on 3D-printed Ni surface, we performed detailed Raman spectroscopy mapping on samples fabricated using optimized parameters (300 W, 0.6 m s^{-1}). The top surface of a printed bulk sample exhibits a graphene coverage over 98% (**Fig. 3a-d** and **Supplementary Fig. 7a-c)**. The Raman I_D/I_G values (all < 0.56) and I_{2D}/I_G ratios, predominantly ranging from 0.3 to 1.34, indicate the formation of FLG with uniformly high quality[33]. Meanwhile, abundant SEM images validate the continuous graphene growth (**Supplementary Fig. 8**). Notably, such conformal growth of high-quality graphene is not restricted to flat horizontal surfaces. High-quality FLG was successfully synthesized on non-planar geometries, including inclined surfaces (15-135°, **Fig. 3e**) and curved hemispheres (**Fig. 3f**), showcasing the flexibility of the in-situ graphene synthesis and its compatibility with the LPBF process. Furthermore, growth can reach complex internal architectures. Raman mapping of the interior surface of a printed gyroid structure (**Fig. 3g** and **Supplementary Fig. 9**) and vertical sidewalls (**Fig. 3h** and **Supplementary Fig. 7e-h**) revealed high graphene coverage rates of 95% and 94%, respectively, confirming the formation of a continuous FLG 3D wrapping on the metal scaffold during printing. The few areas lacking Raman signal are attributed to topological undulations of the gyroid structure and defocusing of the laser in Raman spectroscopy (**Supplementary Fig. 9g** and **h**).

3. The authors mentioned that the 3D graphene network has formed in the Ni matrix, but the Raman spectra and CT data has failed to show the microstructures of the 3D graphene. Some other advanced techniques are recommended for the determination of a 3D graphene network.

Reply:

We sincerely appreciate the reviewer's constructive suggestion on the structural characterization of the 3D graphene network within the metal matrix. It is acknowledged that fully and directly visualizing the 3D architecture of the in-situ grown graphene in the final composite, for example the dense Ni-MMCs, is exceptionally challenging for the following reasons: (i) the total graphene content generated via carbon precipitation during the rapid solidification/cooling of LPBF is intrinsically low, although its crystallinity and quality — confirmed by TEM and Raman spectroscopy — are superior to those obtained by conventional powder metallurgy based routes. Notably, the low graphene content is beneficial for this system, because nickel tends to form multilayer graphene with higher defect density when excess carbon is available; the limited carbon precipitation flux due to ultrafast cooling in LPBF helps maintain few-layer, high-quality sp^2 structures (HRTEM image in **Supplementary Fig. 5**). (ii) direct tomographic imaging (e.g., by SEM, FIB-tomography, or μ-CT) of low-content, atomically thin graphene layers embedded within a high-density metal matrix like nickel remains beyond the current capability of these techniques. Given these constraints, an indirect, multi-perspective evidence was established here and in the revised manuscript to confirm the formation, spatial continuity, and structural retention of the 3D graphene network.

(1) Direct confirmation on the as-printed scaffold. Before hot pressing, confocal Raman mapping was performed on both the external curved surfaces and the internal pore surfaces of the as-printed gyroid Ni scaffold. The results show uniform coverage of characteristic graphene G and 2D bands across the entire scanned area, with low I_D/I_G ratios (< 0.56), confirming that a continuous, high-quality graphene layers conformally coat the 3D Ni skeleton right after LPBF printing (**Supplementary Fig. 9**). SEM imaging of the same scaffold surface further reveals continuous wrinkle structures typical of in-situ grown graphene (**Supplementary Fig. 10**).

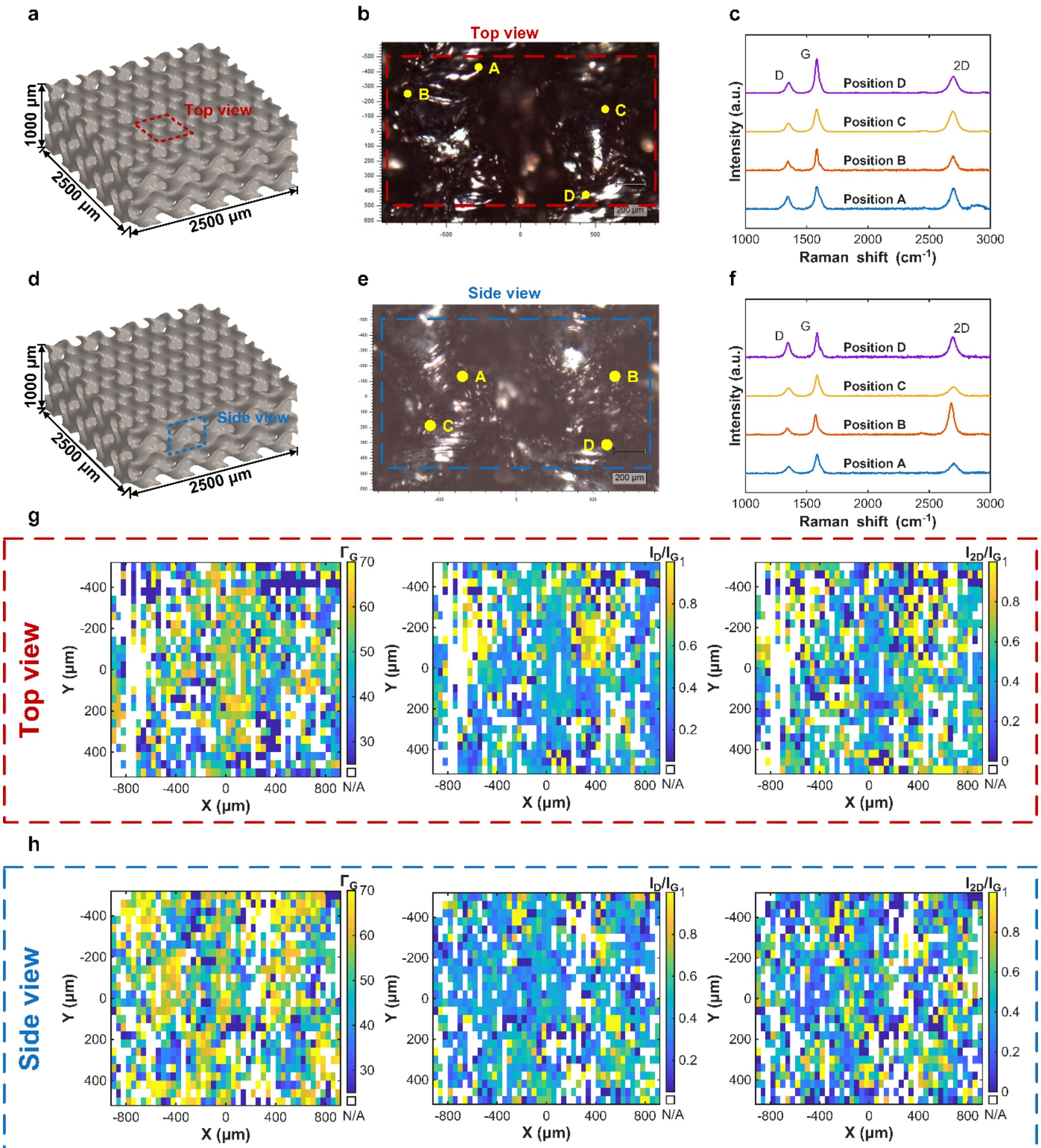

Supplementary Fig. 9: Characterizations of FLG grown on the curved surface of a printed gyroid Ni structure.

a, **d** Gyroid structure 3D model. **b**, **e** OM of printed gyroid Ni-Gr composite's top surface and lateral surface. **c**, **f** Representative Raman spectra of different position in (**b**) and (**e**), respectively. **g**, **h** Corresponding Raman results in (**b**, **e**).

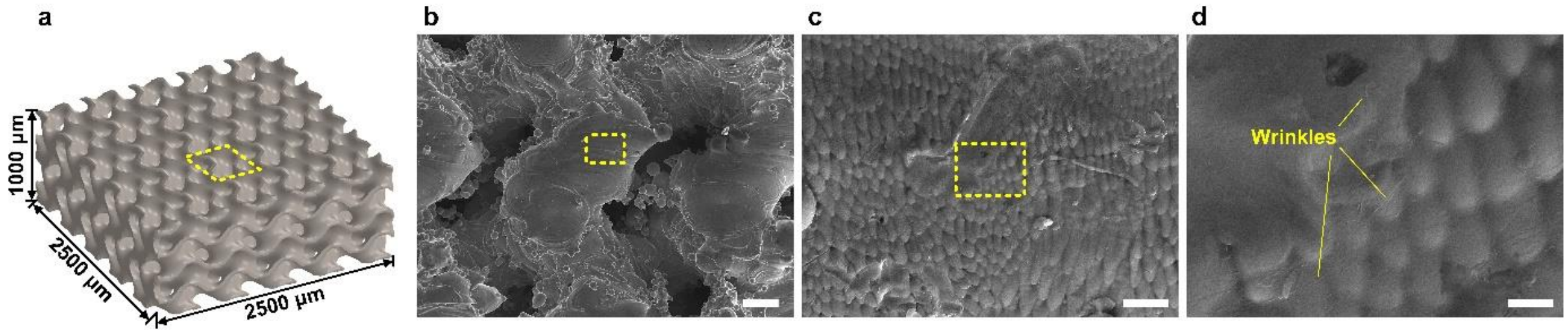

Supplementary Fig. 10: SEM images of in-situ grown graphene on as-printed gyroid skeleton.

a Gyroid structure 3D model. **b**, **c**, **d** SEM images of as-printed composite skeleton, where graphene uniformly grows on its surface and yellow lines point to the unique wrinkle structure of graphene. Scale bar, 200 μm (**b**), 5 μm (**c**), 1 μm (**d**).

(2) Indirect visualization of the preserved 3D network via GNDs patterning. After hot pressing and densification, cross sections of the final Ni/PMMA-Lattice-HP composite were analyzed by EBSD KAM mapping. The KAM map (**Fig. 4j** and **Supplementary Fig. 11**) exhibits a periodic, long-range pattern of geometrically necessary dislocations (GNDs) density that precisely matches the gyroid topology. This periodic GNDs distribution originates from the continuous 3D graphene network: the Ni–Gr interface acts as a strong barrier to dislocation motion during hot pressing and subsequent plastic deformation, forcing dislocation pile-ups that follow the spatial layout of the reinforcement network. In contrast, the graphene-free control sample (Ni-Lattice-HP, **Fig. 4k**) shows an irregular, random GNDs distribution with no periodic pattern. Therefore, we believe the KAM pattern serves as a reliable fingerprint of the underlying graphene architecture.

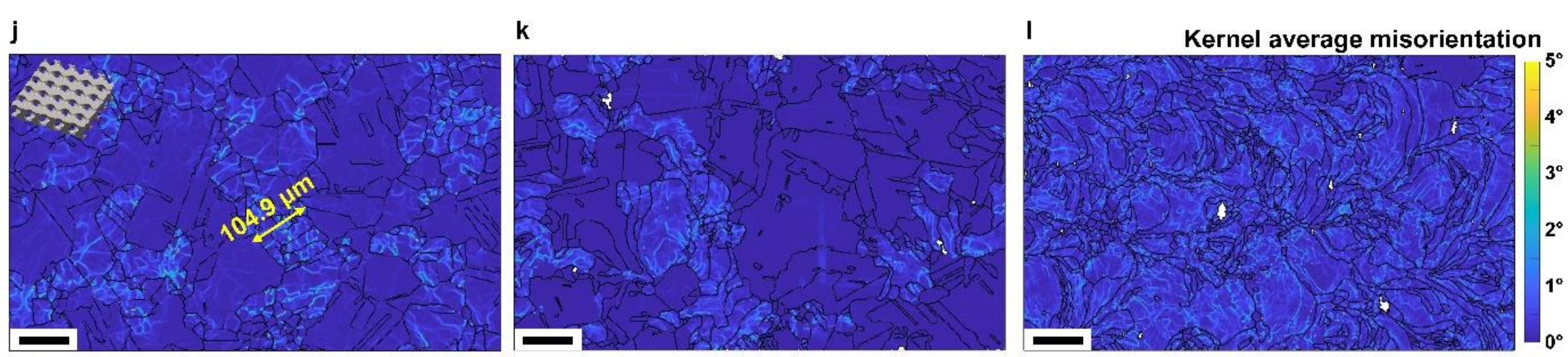

Fig. 4 j, k, l: Microstructure characterizations of as-fabricated Ni and Ni-Gr composites

j, **k**, **l** The kernel average misorientation maps of Ni/PMMA-Lattice-HP, Ni-Lattice-HP, and Ni/PMMA-Bulk, respectively. The geometrical necessary dislocations (GNDs) density is high in the LPBF-printed regions due to fast heating and cooling. The Ni/PMMA-Lattice-HP, the KAM map shows a periodic pattern as the printed gyroid Ni-Gr scaffold, scale bar, 100 μm (**j**-**l**).

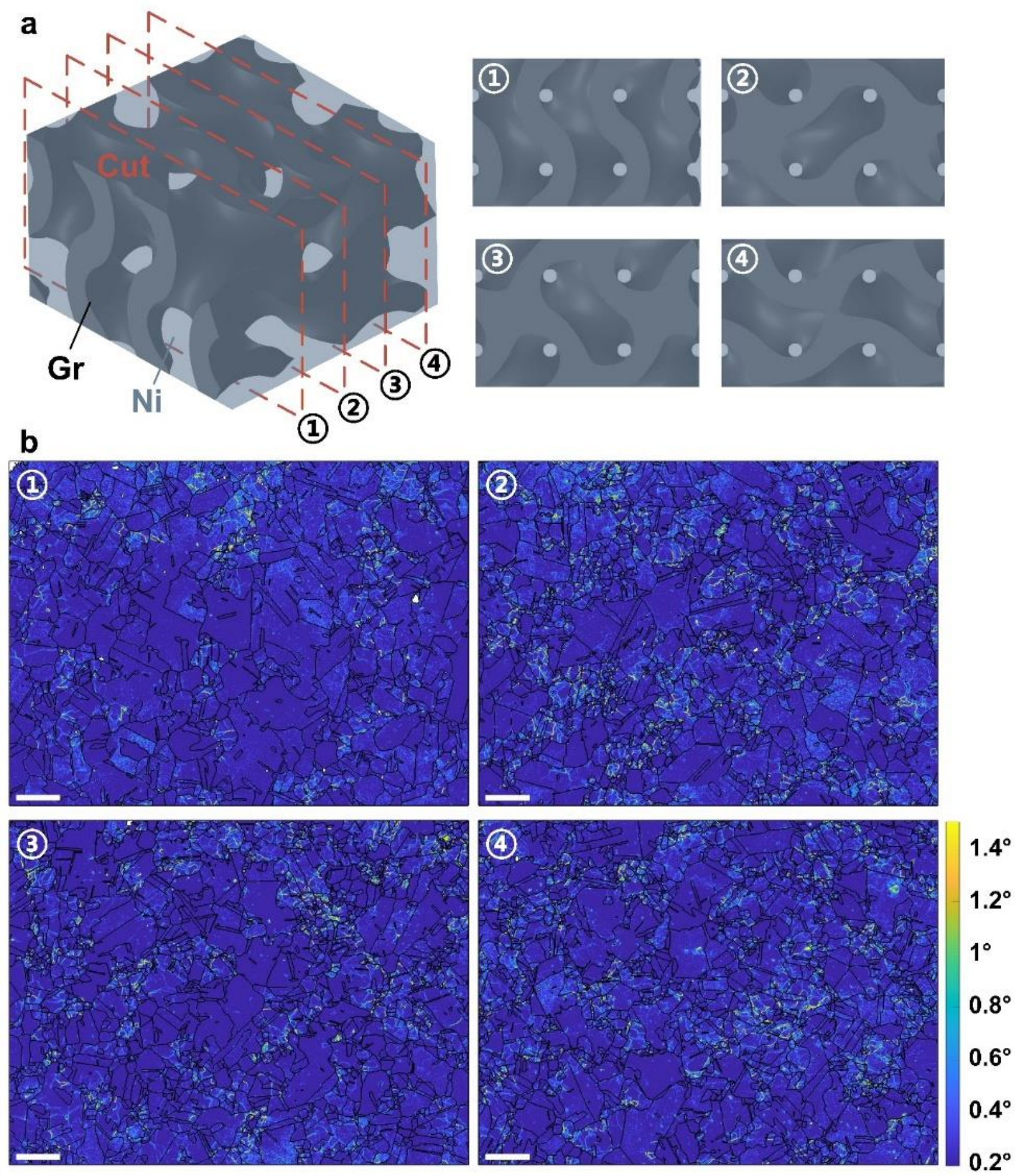

Supplementary Fig. 11: KAM images of Ni/PMMA-Lattice-HP sample.

a Ni/PMMA-Lattice-HP KAM sample cutting schematic, showing the possible distribution of the graphene network. **b** Corresponding KAM images to the slices in (**a**). Scale bar, 150 μm (**b**).

(3) Gr-Ni interface deformations during densification. To further assess whether the as-grown graphene gyroid configuration could maintain its structural integrity during the hot-pressing densification step, finite element method (FEM) analysis has been conducted to illustrate the deformation of the as-printed Ni skeleton and the encapsulated unmolten powders during hot pressure. A 2×2×2 representative volume

element model is established to simulate the deformation with a symmetric boundary condition. Both Ni/PMMA-Bulk and powder matrix are meshed with linear tetrahedral finite element. Elasto-plastic model is applied to simulate the constitutive behavior of both parts. The parameters are obtained from experiments and a reduction based on the packing density for Ni/PMMA-bulk and Ni-PMMA powder respectively. The model is loaded with a rigid plate meshed in quadrilateral shell element with general contact, up to a strain of 20%. The explicit method is conducted for FEM analysis and mass scaling is applied for higher computing efficiency. Additionally, the total mass increment ratio is restrained below 1%, leading to a rational result.

FEM results are shown in **Supplementary Fig. 12**. The deformed configuration maintains the gyroid-like curvy shape (**Supplementary Fig. 12b** and **c**), which keeps a good consistency with EBSD results (**Supplementary Fig. 11**). The maximum Von Mises stress between Ni/PMMA powder and Ni TPMS skeleton is below 650 MPa, which is lower than the critical value for graphene tearing or delamination, provided that the graphene is well bonded. The maximum true strain in skeleton structure is about 0.3, which obviously exceeds the ductility of graphene. However, between two peak values, the strain remains quite low at around 0, which partially keeps the connectivity of graphene layers, leaving continuous 3D Gr network in Ni/PMMA-Lattice-HP composite after hot pressing.

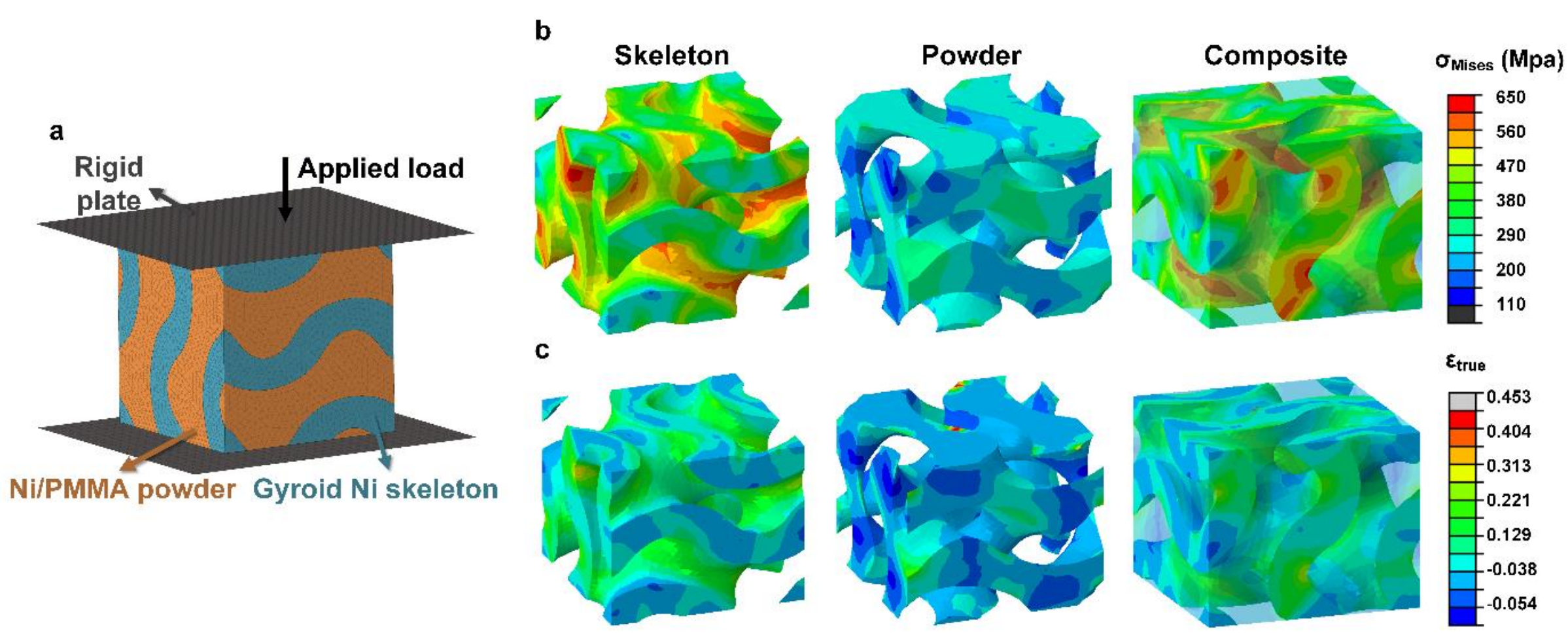

Supplementary Fig. 12: Simulated compression deformation of Ni skeleton-Ni/PMMA powder composite.

a Finite element method model. **b**, **c** Von Mises stress and true strain of Ni skeleton, Ni/PMMA powder, and composite during compression deformation.

Overall, while direct tomographic imaging of the graphene network within the bulk nickel is not feasible with current techniques, the combination of (i) Raman spectroscopy/SEM confirmation on the as-printed scaffold, (ii) topology-matched periodic GNDs patterning in the final composite, and (iii) FEA of interfacial integrity provides compelling, multi-scale evidence for the formation, preservation, and structural integrity of the designed high-quality 3D graphene architecture.

To validate the 3D graphene network within Ni/PMMA-Lattice-HP composite, we have added new figures to SI, including **Supplementary Fig. 7, 8, 9, 10, 11**, and **12**. Besides, deep discussion to clarify the 3D graphene networks has been added to SI as well (Supplementary Note 6, pages 13-14, lines 160-195).

Supplementary Note 6. Structural characterizations of the grown FLG on the printed Ni scaffold surface during LPBF process and within the Ni matrix after densification

Extensive Raman spectroscopy characterizations demonstrate the high-quality growth of graphene on various printed architectures. For example, the graphene coverage rate exceeds 98% on the top surface and 94% on the side surfaces of Ni/PMMA-Bulk samples (**Supplementary Fig. 7** and **Supplementary Fig. 8**). Meanwhile, continuous high-quality graphene growth is also realized on the as-printed composite Gyroid skeleton (**Supplementary Fig. 9** and **Supplementary Fig. 10**).

In addition, EBSD KAM results of Ni/PMMA-Lattice-HP exhibit a periodic pattern of high GND density that matches well with the Gyroid geometry (**Fig. 4j** and **Supplementary Fig. 11**). This typical pattern arises from thermal stress differences between the printed Ni framework and consolidated powder regions, together with dislocation blocking at their interfaces. At these interfaces, the growth of a three-dimensional continuous graphene network has been confirmed by Raman and SEM characterizations. Furthermore, hot pressing does not damage the continuity and integrity of the 3D graphene network in Ni/PMMA-Lattice-HP samples. Accordingly, FEM simulations were carried out to illustrate the stress-strain distribution between the Ni skeleton and Ni/PMMA powder during compressive deformation.

A 2×2×2 representative volume element model is established to simulate the deformation with a symmetric boundary condition. Both Ni/PMMA-Bulk and powder matrix are meshed with linear tetrahedral finite element. Elasto-plastic model is applied to simulate the constitutive behavior of both parts. The parameters are obtained from experiments and a reduction based on the packing density for Ni skeleton and Ni/PMMA powder respectively. The model is loaded with a rigid plate meshed with quadrilateral shell element with general contact, up to a strain of 20%, according to the actual experimental

value. The model is shown in **Supplementary Fig. 12a**. The explicit method is conducted for FEM analysis and mass scaling is applied for higher computing efficiency. Additionally, the total mass increment ratio is restrained below 1%, leading to a rational result.

FEM results are shown in **Supplementary Fig. 12b** and **c**. The deformed configuration maintains the gyroid-like curvy shape, which keeps a good consistency with EBSD KAM results (**Supplementary Fig. 4j** and **Supplementary Fig. 11**). The stress concentrates at the peaks that tangents parallel to loading direction, and between the peaks, the stress level is much lower. As shown in **Supplementary Fig. 12c**, the maximum true strain in skeleton structure is about 0.3, which obviously exceeds the ductility of graphene. However, between two peak values, the strain of the most region remains quite low at around 0, which partially keeps the connectivity of graphene layers, leaving continuous 3D Gr network in Ni/PMMA-Lattice-HP composite after hot pressing.

Corresponding figures are also mentioned in main text (page 17, lines 268-275).

As compared in **Fig. 4j-l**, the Ni/PMMA-Lattice-HP sample displays a distinct, periodic pattern of high geometrically necessary dislocation (GND) density that mainly replicates the designed gyroid scaffold. This patterned dislocation density originates from the strain incompatibility and impeded dislocation motion at the interfaces between the printed Ni framework and the consolidated powder regions, where the FLG grows (**Supplementary Fig. 11**). Furthermore, FEM results verify that and the as-grown FLG are not fully disrupted after hot pressing, therefore the continuity of the 3D graphene architecture remains preserved (**Supplementary Fig. 12**).

4. A hot-pressing process has been used after the laser additive manufacturing. Why the hot-pressing is applied? Is it because the sintering density of the composite is not high, or the mechanical property of the initial state of the composite is not good? In fact, the 3D graphene network would generate in one-step hot-pressing process. The combination of laser-sintering and hot-pressing makes a loss of the advantages of the laser-sintering. On the other hand, as is known, the hot-pressing may damage the structures of graphene and lead an anisotropic Ni matrix, which influence the mechanical property of the composite. Thus, the role of hot-pressing should be stated.

Reply:

We are grateful for the reviewer's important question regarding the necessity of the hot-pressing (HP) step and its potential effects on the graphene network and the matrix anisotropy. We clarify below that HP is an indispensable and deliberately designed component of the proposed manufacturing route, not a remedial measure for poor sintering density or inferior mechanical properties of the LPBF-printed parts (note, all LPBF printing in this study achieved over 99.5% density of the materials). Moreover, the two-step approach (LPBF+HP) retains the core advantages of laser AM — specifically, the ability to predefine the 3D topology of the graphene architecture within the metal matrix — which cannot be achieved by one-step powder metallurgy-based HP method, which leads to random graphene network.

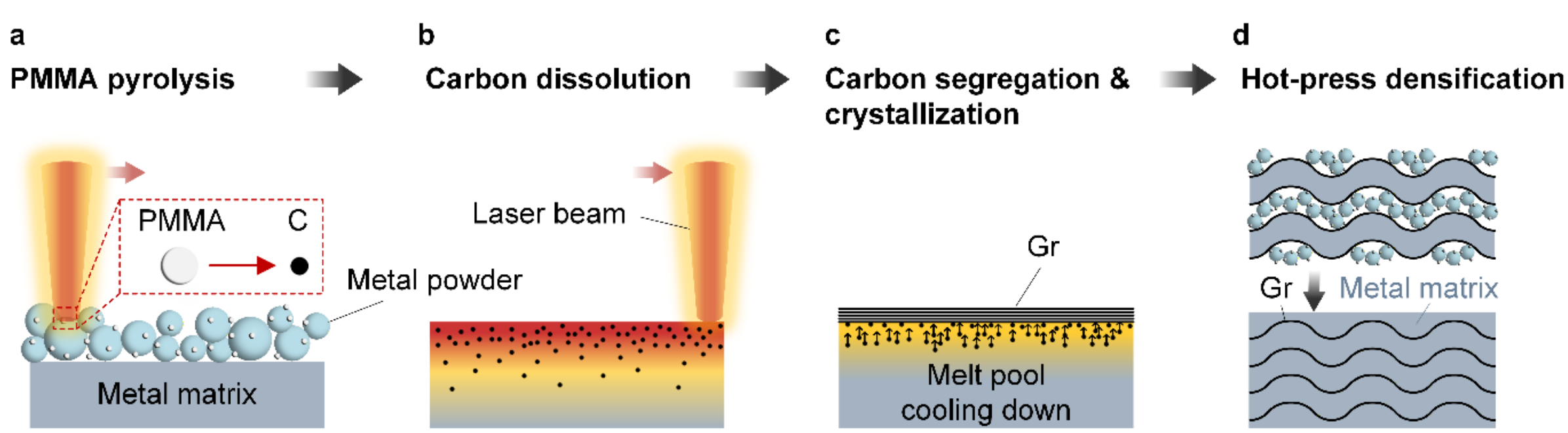

Supplementary Fig. 1: In-situ graphene growth mechanism during LPBF.

The in-situ LPBF fabrication of Gr-MMCs involves four primary procedures: **a** Metal powders are melted to form a molten pool, and PMMA particles are pyrolyzed into free carbon atoms; **b** Carbon atoms dissolve into the high-temperature molten pool; **c** During molten pool cooling, carbon atoms segregate to the surface and crystallize to form conformal graphene layers; **d** Gr-MMCs with designed spatial architectures are densified via hot pressing.

A central innovation of this work is the spatial control of the 3D graphene architecture via LPBF AM. This pre-designed nano-reinforcement architecture is the root cause of the MMCs' exceptional strength-ductility synergy and high graphene strengthening efficiency of ultimate tensile strength (≈761% improvement over pure Ni, as shown in **Fig. 5e**). In contrast, one-step HP of pre-mixed metal powders and carbon precursor usually produces a random distribution of graphene within the Ni matrix, offering limited ability to design or control the 3D reinforcement architecture. In the present strategy, LPBF is used to predefine the 3D topology of the graphene network by using a printed Ni 3D template. Notably, during in-situ LPBF printing, graphene layers directly form on the surface of as-printed Ni scaffold, while the unscanned (unmolten) Ni powders are encapsulated inside of the Ni scaffold pores. Subsequent HP on the graphene wrapped scaffold together with intentionally encapsulated unmolten powders serves only to densify the structure while preserving the pre-designed architecture. **Supplementary figure 1** elaborates the detailed fabrication processes.

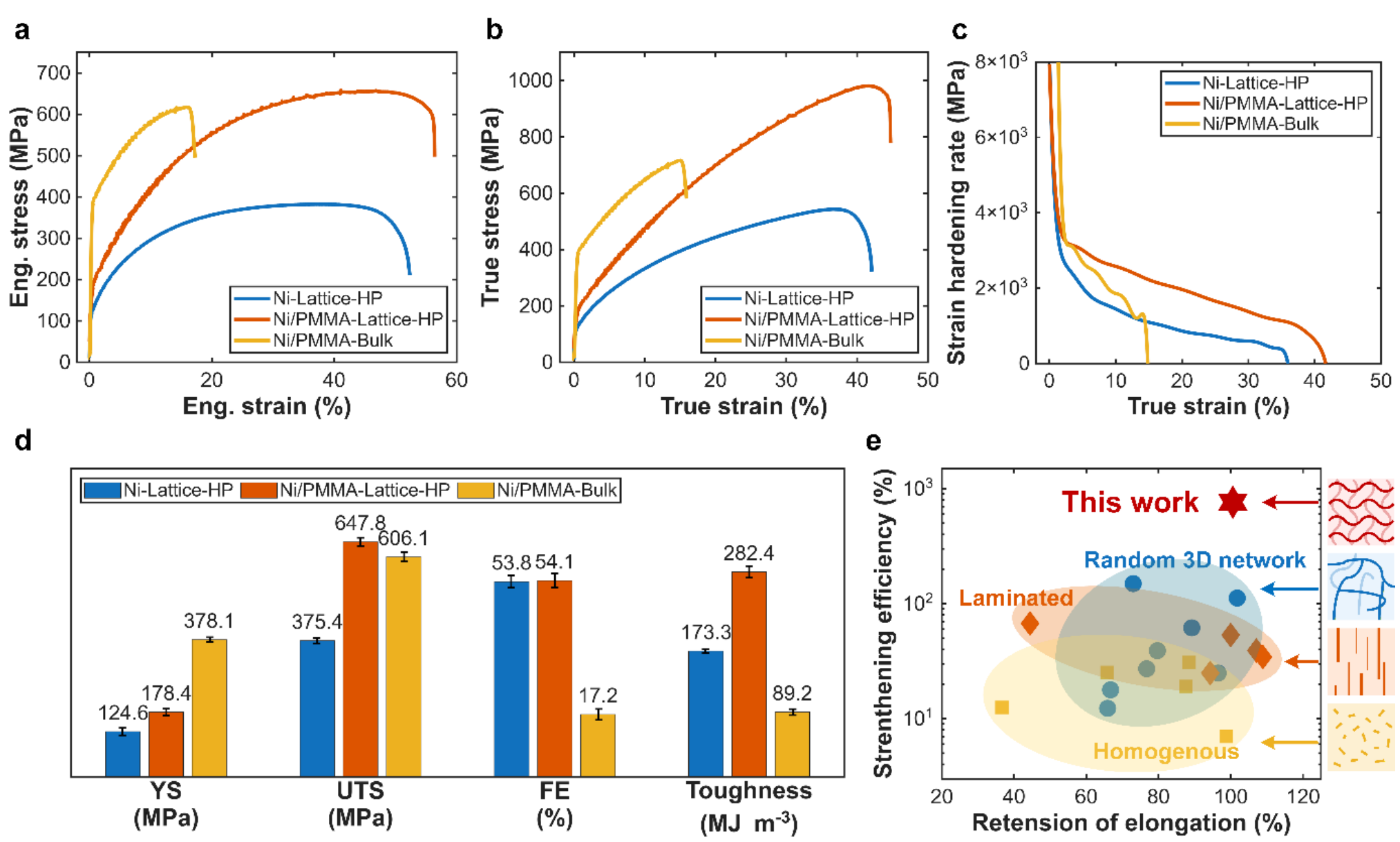

Fig. 5: Uniaxial tensile properties of as-fabricated Ni and Ni-Gr composites.

a, b Engineering and true tensile stress-strain curves of Ni/PMMA-Lattice-HP, Ni-Lattice-HP, and Ni/PMMA-Bulk, respectively. **c** Strain hardening rate – true strain curves. **d** Comparison of key mechanical properties of Ni/PMMA-Lattice-HP, Ni-Lattice-HP, and Ni/PMMA-Bulk, showing that the as-prepared Ni/PMMA-Lattice-HP composite had an outstanding combination of strength and toughness. **e** Retention of fractural elongation versus strengthening efficiency of graphene, showing superior

mechanical performance of 3D printed graphene reinforcement architecture compared to homogeneous, laminated, and random 3D network configurations.

The necessity of HP is further evidenced by comparing the mechanical properties of the as-printed (Ni/PMMA-Bulk) and HP-densified (Ni/PMMA-Lattice-HP) samples (**Fig. 5a**). The as-printed bulk sample exhibits significantly lower ultimate tensile strength and fracture elongation. Two factors contribute to this: (i) the pyrolysis of PMMA during LPBF releases large amounts of carbon-containing gases, resulting in a relative density of only ≈99.5% even at high volumetric energy density, as shown in the CT images in **Supplementary Fig. 15**; and (ii) the inherent layer-by-layer nature of LPBF causes the high-quality graphene layer on each solidified surface to be torn apart during subsequent laser melting of the next powder layer (**Supplementary Fig. 13a** and **b**). Consequently, the as-grown graphene in the as-printed bulk sample is eventually fragmented, which cannot effectively harness the expected strengthening mechanisms associated to graphene in-plane properties.

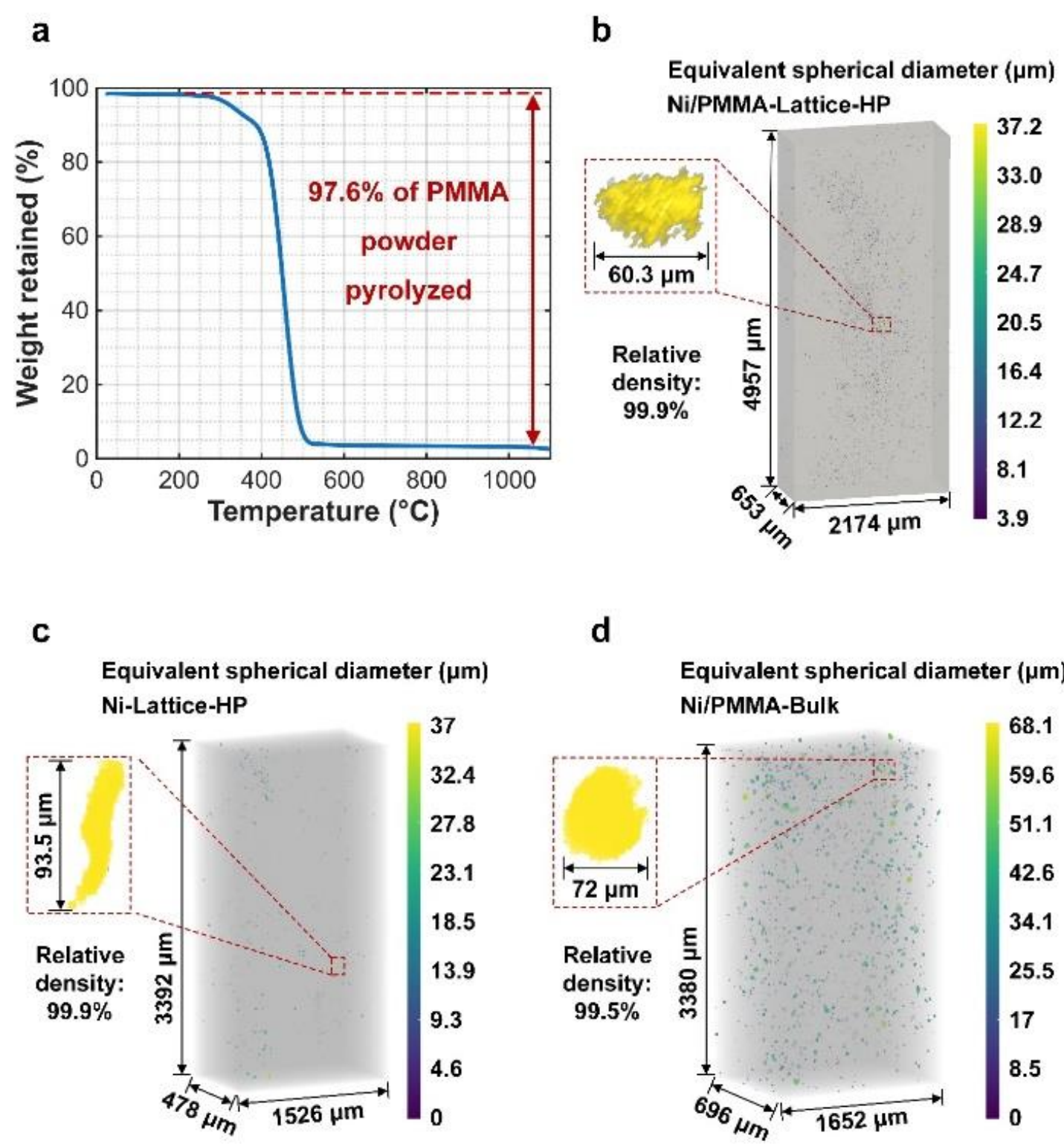

Supplementary Fig. 15: TGA and CT analysis of the fabricated Gr-Ni composites.

a The TGA analysis showing 97.6% of PMMA powders can be fully pyrolyzed at temperatures over 500˚C. **b**, **c**, **d** CT analysis of Ni/PMMA-Lattice-HP, Ni-Lattice-HP, and Ni/PMMA-Bulk samples showing 99.9%, 99.9%, and 99.5% relative density respectively. Pores are color-coded by equivalent spherical diameter.

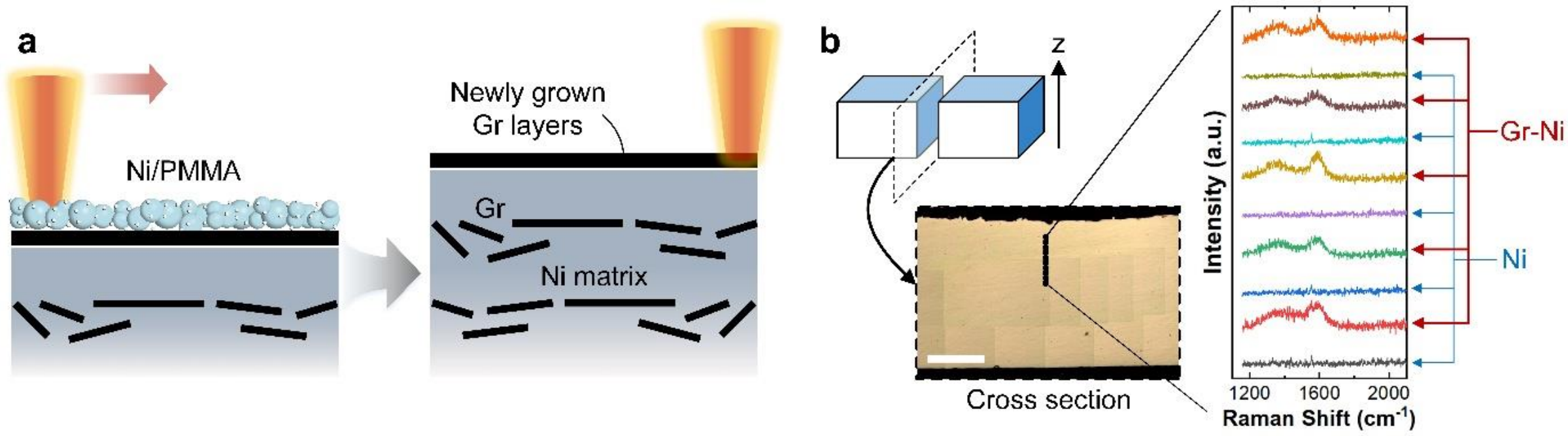

Supplementary Fig. 13: The as-grown Gr distribution within the Ni/PMMA-Bulk.

a Schematic illustration showing that as-grown FLG on the surface of cladding was torn and distorted as the laser melting of the next layer of powders, and a new layer of Gr grew on the top surface of cladding. **b** OM image and Raman spectra of the cross-sectional surface of the printed Ni-Gr composite, showing periodic graphitization phenomenon, scale bar 1 mm (**b**).

Regarding the concern that HP might damage the graphene structure or induce anisotropy: first, the EBSD KAM map of the HP-densified Ni/PMMA-Lattice-HP sample (**Fig. 4j** and **k**) exhibits a distinct, periodic pattern of high GNDs density that precisely replicates the pre-designed gyroid Ni scaffold. This pattern provides direct microstructural evidence that the 3D graphene network remains structurally intact and retains its designed topology after HP. Second, the pole figure map of the same sample (**Fig. 4g**) shows a maximum texture intensity of only 4.66, which is significantly lower than that of the graphene-free control (maximum texture intensity=7.28, **Fig. 4h**), indicating no strong crystallographic texture. These results demonstrate that the HP step does not damage the pre-defined graphene architecture nor introduce undesirable anisotropy.

Thus, the role of HP is clearly defined: it densifies the LPBF-pre-architectured scaffold without compromising the graphene network or inducing anisotropy, enabling the realization of a fully dense Gr-MMC with a designed 3D reinforcement architecture — a capability not achievable by either LPBF alone or one-step powder metallurgy-based HP fabrication.

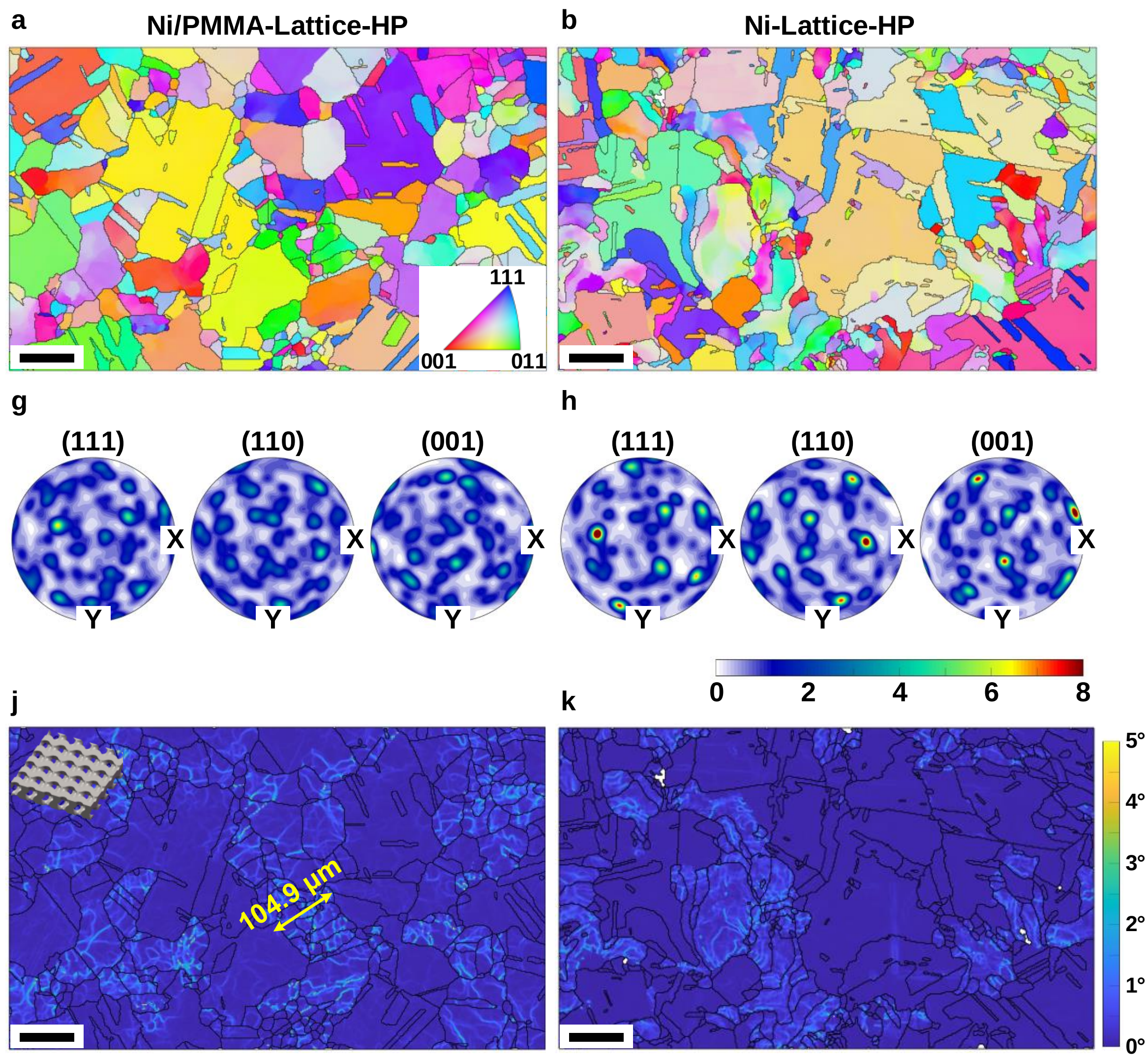

Fig. 4a, b, g, h: Microstructure characterizations of as-fabricated Ni and Ni-Gr composites.

a, **b** EBSD inverse pole figure (IPF) maps of the proposed Ni MMC with in-situ grown graphene network reinforcement (Ni/PMMA-Lattice-HP), and control group densified LPBF printed pure Ni lattice via hot pressing (Ni-Lattice-HP), scale bar, 100 μm (**a**, **b**). **g**, **h** Pole figure (PF) maps of Ni/PMMA-Lattice-HP and Ni-Lattice-HP respectively. Notably, for Ni-Lattice-HP without graphene synthesis, a clear texture pattern can be observed. **j**, **k**, The kernel average misorientation maps of Ni/PMMA-Lattice-HP, Ni-Lattice-HP and respectively. The geometrical necessary dislocations (GNDs) density is high in the LPBF-printed regions due to fast heating and cooling. The Ni/PMMA-Lattice-HP, the KAM map shows a periodic pattern as the printed gyroid Ni-Gr scaffold, scale bar, 100 μm (**j**, **k**).

We have added **Supplementary Figure 1** to SI to illustrate the detailed mechanism of in-situ graphene growth during LPBF, which has been mentioned in main text (page 7, line 114).

The released carbon atoms can diffuse into the molten Ni, and during the Ni melt pool solidifies the dissolved carbon can precipitate to the Ni clad surface to form graphene layers (**Supplementary Fig. 1**).

CT data for Ni-Lattice-HP and Ni/PMMA-Bulk sample has been added to **Supplementary Fig. 15c and d** and mentioned in main text (page 20, lines 293-299).

Prior to mechanical testing, thermogravimetric analysis (TGA) was applied to the pre-mixed Ni/PMMA powders and confirmed about 97.6% decomposition of the PMMA precursor at temperatures over 500 ˚C, with only 2.4% residual carbon at 1100 °C (**Supplementary Fig. 15a**). Further, computed tomography (CT) scans revealed the density of the as-fabricated Ni/PMMA-Lattice-HP, Ni-Lattice-HP, Ni/PMMA-Bulk samples over 99.9%, 99.9%, and 99.5% respectively. All specimens were free of macro-defects, confirming that the proposed in-situ LPBF process does not compromise structural integrity (**Supplementary Fig. 15b-d**).

5. There is also less information about the interfacial structure analysis. The interfacial structures of a composite are important for its mechanical response. Thus, the interface of the graphene/Ni composites should be clarified.

Reply:

We appreciate the reviewer's professional and insightful comments. The reviewer raises an important point regarding the interfacial structure of the Gr-Ni MMC, which is indeed central to understanding the mechanical response. In response, we prepared new samples and performed high-resolution transmission electron microscopy (HRTEM) on the as-fabricated Gr-Ni interface. The results have been incorporated into the revised manuscript.

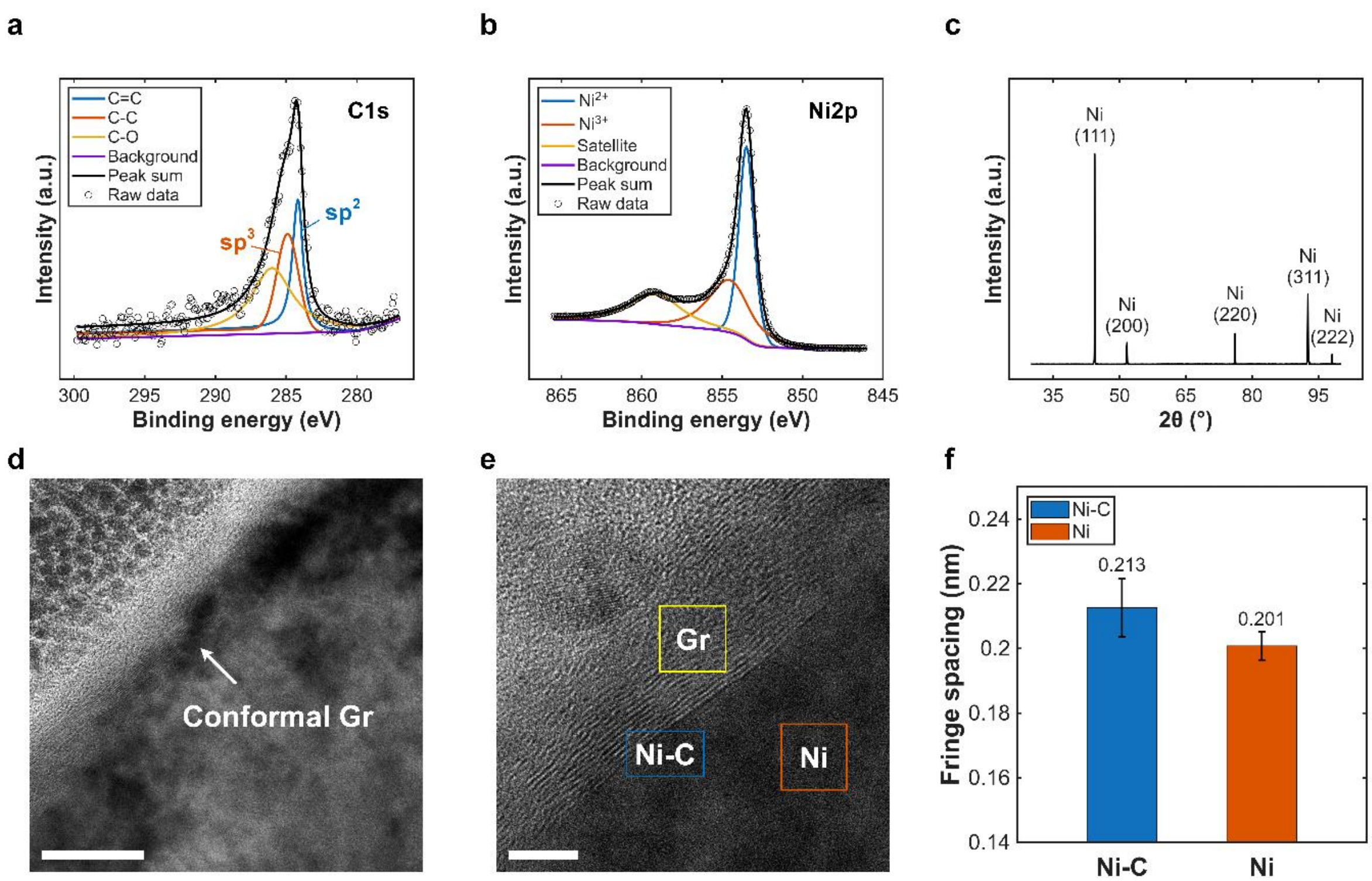

Supplementary Fig. 5: Gr-Ni interface characterization.

a, **b**, **c** XPS C1s, XPS Ni2p, and XRD spectrum of Ni/PMMA-Lattice-HP sample. **d**, **e** HRTEM images of Gr-Ni interface. **f** Fringe spacing comparison of Ni region and Ni-C region. Scale bar, 5 nm (**d**, **e**).

As shown in **Supplementary Fig. 5**, the HRTEM analysis reveals two key features of the Gr-Ni interface. First, the interface between the in-situ grown FLG and the Ni matrix is continuous, intimate, and free of voids, microcracks, impurity phases, or agglomerated carbon clusters. It has been reported in the literature

that the interfacial adhesion energy of graphene grown on Ni is 72.7 J/m², approximately six times that of Cu, which leads to a stronger interaction at the Gr–Ni interface[7]. Second, lattice fringe analysis shows that the Ni matrix exhibits a spacing of approximately 0.20 nm, corresponding to the (111) plane of face-centered cubic Ni[5]. At the Gr-Ni interface, a distinct Ni–C transition region is present, where the lattice spacing gradually shifts from 0.20 nm to 0.23 nm. This variation indicates partial diffusion and segregation of carbon atoms at the Ni subsurface during graphene growth, forming a Ni-C interfacial layer that ensures strong adhesion and efficient load transfer across the interface.

These observations provide direct evidence for the high-quality interfacial bonding achieved by the in-situ synthesis strategy, which underpins the observed strengthening and toughening mechanisms.

To discuss the Gr-Ni interface characterization results, **Supplementary Figure 5** and Supplementary Note 4 (Page 9, lines 93-119) have been added to SI.

Supplementary Note 4. Characterization of Gr-Ni interface formed in LPBF process

First-principles calculations indicate that monoatomic carbon on the Ni surface prefers to occupy subsurface octahedral interstitial sites rather than surface adsorption sites, and the chemical potential of subsurface carbon is significantly lower than that of surface carbon[1]. This thermodynamic characteristic renders carbon highly prone to diffuse into the matrix during deposition on the Ni surface and eventually form a nickel carbide phase[1], which has been confirmed by Li's team[2].

In the XPS and XRD results of Ni/PMMA-Lattice-HP sample (**Supplementary Fig. 5a-c**), there are no characteristic peaks of Ni carbides observed, for example, diffraction peaks at 39.3°, 41.7°, and 58.6° in XRD, which is respectively related to the (110), (006), and (116) planes of Ni_3C structure (PDF#06-0697). The absence of nickel carbide peak can be attributed to the high temperature during LPBF and subsequent hot-pressing treatment, where the metastable Ni carbides could decompose, and the released carbon atoms will diffuse onto the graphene layer[3]. The ultra-low graphene content (0.095 vol.%) in the composite makes carbides undetectable by the employed characterization methods.

The cross-sectional HRTEM characterizations of the Ni/PMMA-Bulk sample helps to better understand the microstructure between the as-grown graphene and underneath Ni matrix, where the Ni carbides are most likely to form. The HRTEM images clearly show an intimate, defect-free semi-coherent interface between the Ni matrix and the graphene layers (**Supplementary Fig. 5d and e**). It is attributed to the high adhesion energy of graphene grown on Ni (72.7 J m^{-2}), which is around 6 times of that Gr-Cu interface[7].

Notably, the lattice fringe spacing of the Ni matrix exhibits a spacing of approximately 0.20 nm, corresponding to the (111) plane of face-centered cubic Ni[5]. While a distinct Ni–C transition region is present, where the lattice spacing gradually shifts from 0.20 nm to 0.23 nm (**Supplementary Fig. 5f**). This gradual change indicates partial diffusion and segregation of carbon atoms at the Ni subsurface during graphene growth, forming a Ni-C interfacial layer that ensures strong adhesion and efficient load transfer across the interface.

Corresponding modifications have also been updated in the main text (Page 13, lines 191-199).

It is noted that the initial layers of graphene grown on the surface of the Ni matrix typically do not exhibit measurable features in Raman spectra[8]. Cross-sectional high-resolution transmission electron microscopy (HRTEM) image confirmed the presence of FLG growth on the Ni surface (**Fig. 2i**). Notably, no obvious Ni carbide phase[2] was detected within the fabricated composites using X-ray photoelectron spectroscopy (XPS) and X-ray diffraction (XRD), as shown in **Supplementary Fig. 5a-c**. However, a slight lattice distortion of Ni is observed at the Gr-Ni interface, which is most likely induced by the infiltration of carbon atoms and provides direct evidence for the intimate Ni–Gr interface (**Supplementary Fig. 5d-f**, and **Supplementary Note 4**).

In main text, **figure 2i** was replaced with a new HRTEM image.

6. The strain hardening curves of the composites should be provided since it is helpful to determine the strengthening and plastic behaviors of the composite.

Reply:

We sincerely appreciate the reviewer for the valuable suggestion regarding strain hardening curves, which are indeed critical for elucidating the strengthening mechanisms and plastic deformation behavior of the fabricated Gr-MMC. We would like to clarify that the strain hardening curve of the composites has been included in original manuscript already, and we further elaborate on its analysis here to highlight the key insights into the material's deformation behavior.

As shown in **Fig. 5c**, we present the strain hardening rate versus true strain curves for the three key samples: Ni/PMMA-Lattice-HP (our 3D graphene network-reinforced composite), Ni-Lattice-HP (graphene-free control), and Ni/PMMA-Bulk (fragmented graphene without 3D architecture control). This curve directly reflects the dynamic evolution of strengthening and plastic behavior during deformation, with key observations as follows:

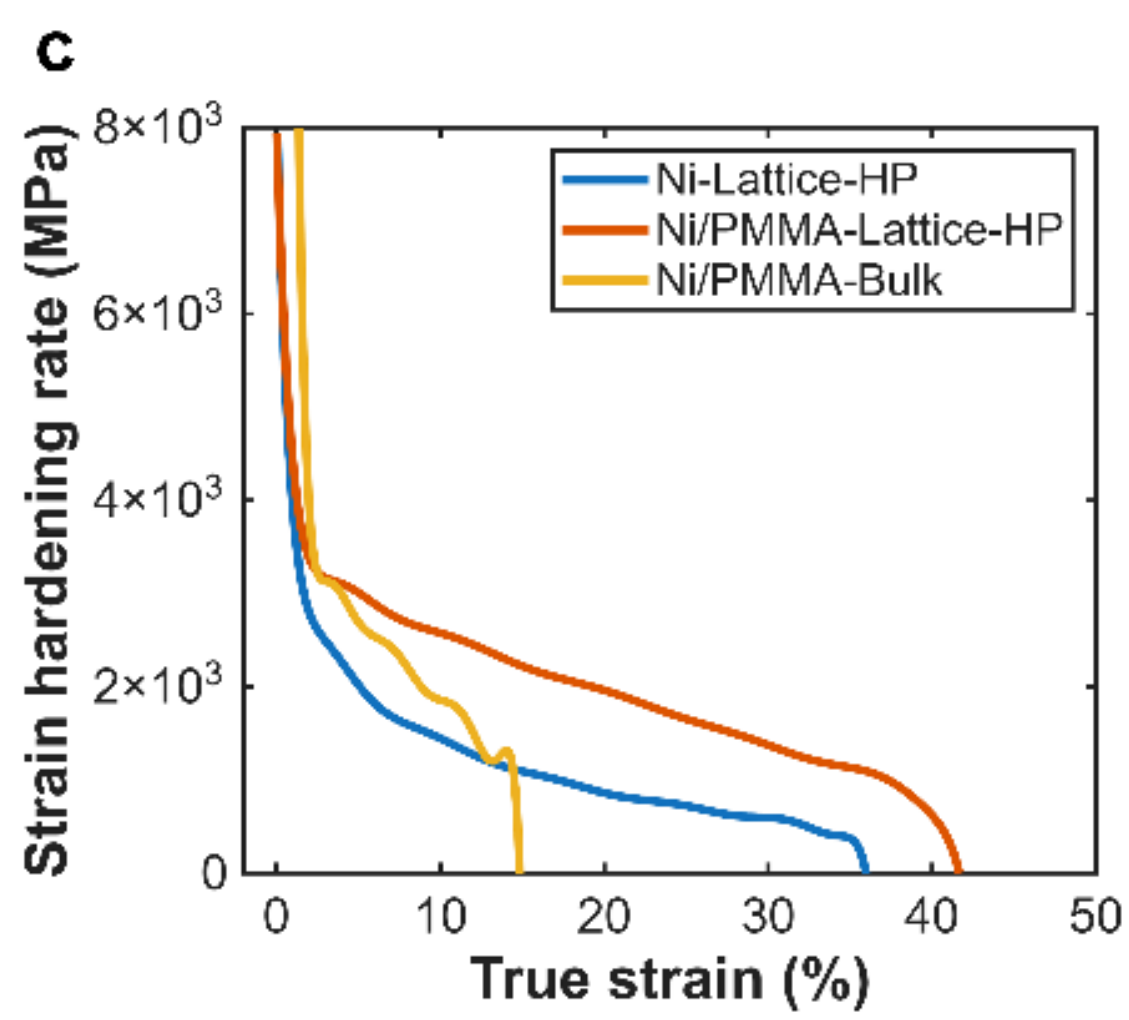

Fig. 5c: Uniaxial tensile properties of as-fabricated Ni and Ni-Gr composites

c Strain hardening rate – true strain curves.

(a) Ni-Lattice-HP (graphene-free control): Exhibits a characteristically slow and continuous decay in strain hardening rate (from ~ 8000 MPa to ~ 2000 MPa) with increasing true strain. This is typical of pure Ni fabricated by LPBF, where dislocation accumulation proceeds uniformly without effective reinforcement, leading to gradual exhaustion of strain hardening capacity[34,35].

(b) Ni/PMMA-Bulk (fragmented graphene): Shows a significantly higher initial strain hardening rate (~ 9500 MPa) due to the dispersion strengthening effect of fragmented graphene. However, the hardening rate drops sharply within a small true strain range (< 0.15), leading to premature necking and limited ductility (fracture elongation = 17.2%). This rapid exhaustion of strain hardening capacity originates from the random distribution of graphene fragments, which act as localized stress concentration sites and impede continuous dislocation multiplication[36,37].

(c) Ni/PMMA-Lattice-HP (3D graphene network): Demonstrates a superior and sustained strain hardening rate throughout the entire deformation process. Its initial hardening rate (~ 9000 MPa) is comparable to that of Ni/PMMA-Bulk, and it maintains a high hardening rate (> 4000 MPa) even at large true strains (> 0.4). This sustained strain hardening is the core reason for the composite's exceptional strength-ductility synergy. The 3D graphene network enables continuous dislocation storage and multiplication by creating heterogeneous deformation zones (evidenced by KAM maps in **Fig. 4j** and **7c**), while avoiding localized stress concentration[38,39]. The hardening rate only begins to decrease gradually at the late stage of deformation, which effectively suppresses premature necking and promotes extensive uniform elongation.

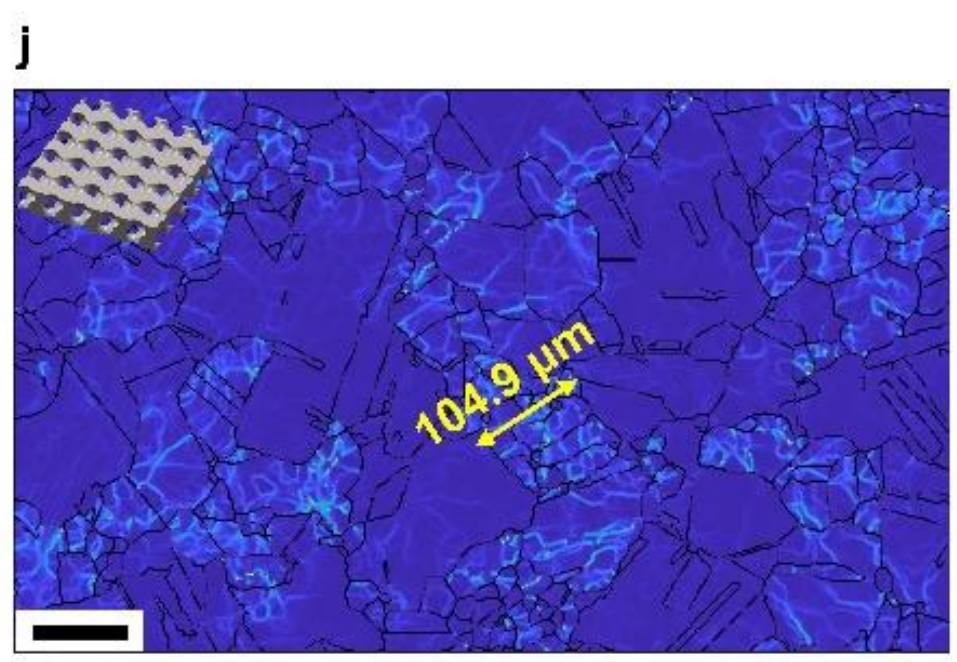

Fig. 4j: Microstructure characterizations of as-fabricated Ni and Ni-Gr composites

j The kernel average misorientation maps of Ni/PMMA-Lattice-HP. The geometrical necessary dislocations (GNDs) density is high in the LPBF-printed regions due to fast heating and cooling. The Ni/PMMA-Lattice-HP, the KAM map shows a periodic pattern as the printed gyroid Ni-Gr scaffold, scale bar, 100 μm (**j**).

c

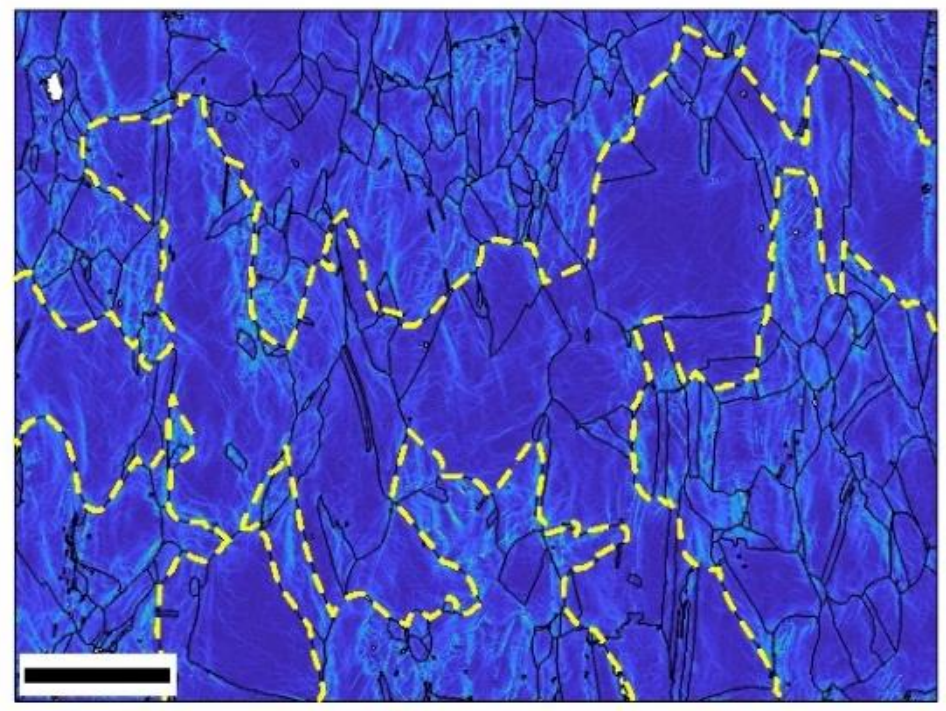

Fig. 7c: Microstructure characterizations of the fractured samples

c KAM map of the fractured Ni-Lattice-HP. Scale bar, 100 μm (**c**).

7. The contribution of 3D graphene in the strength and plasticity of the composite should be analyzed quantitatively.

Reply:

We sincerely appreciate the reviewer for this constructive suggestion. We have calculated different strengthening contributions in our composite, including Hall-petch strengthening, solid solution strengthening, dislocation strengthening, and load transfer strengthening. The theoretical and experimental values show a non-negligible deviation, which we believe is mainly due to the inapplicability of existing load transfer models to calculate 3D nano-reinforcement architecture strengthening mechanisms.

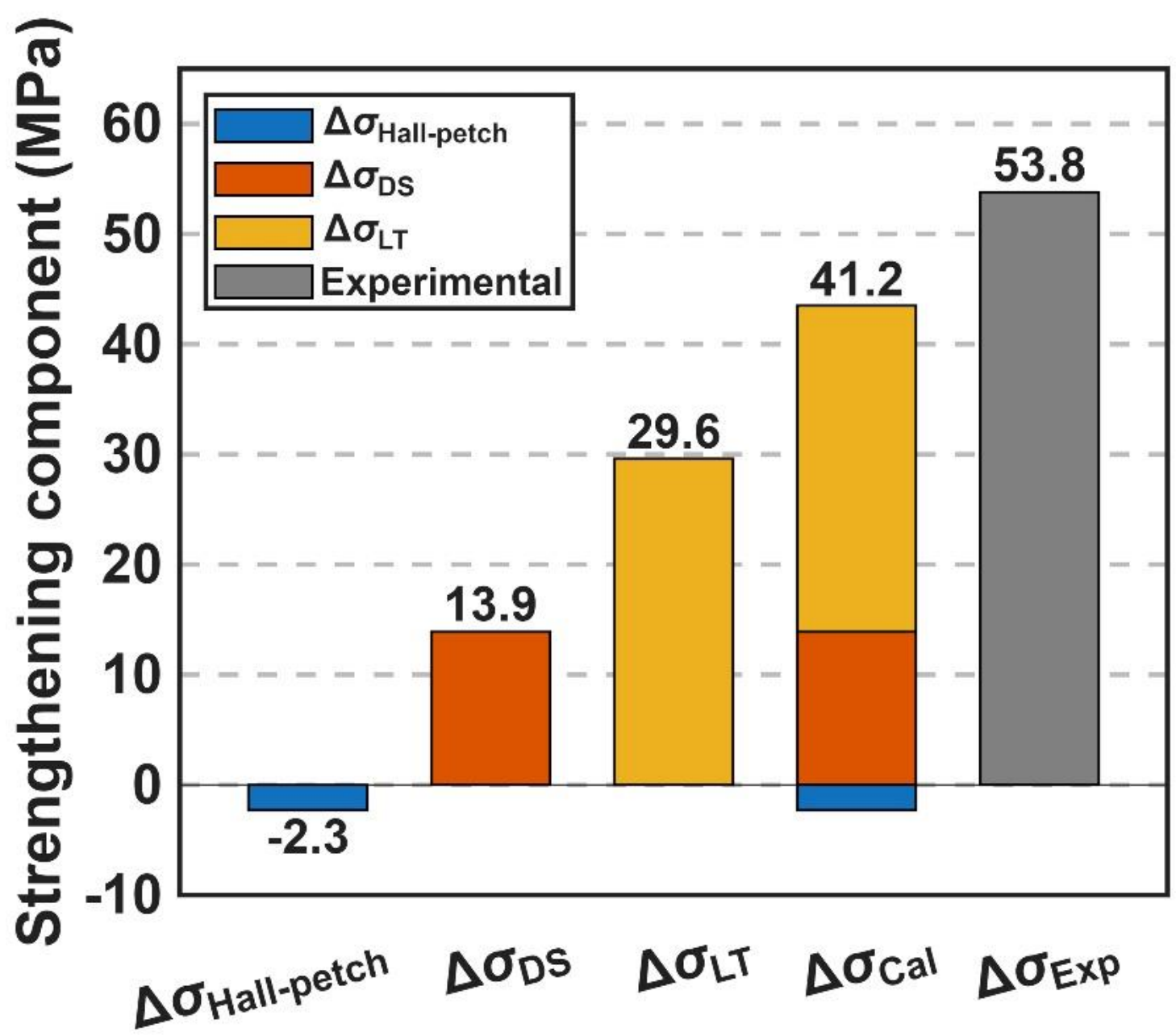

Supplementary Fig. 17: Quantitative strengthening component calculation

Quantitative strengthening component calculations of the Ni/PMMA-Lattice-HP sample compared with the Ni-Lattice-HP sample, containing Hall-petch strengthening, dislocation strengthening, and load transfer strengthening.

The detailed analysis has been added to SI (Supplementary Note 8, Pages 27-32, lines 307-418).

Supplementary Note 8. Quantitative strengthening mechanism

The enhanced yield strength Ni/PMMA-Lattice-HP compared with Ni-Lattice-HP can be estimated by equation:

$$\sigma_C = \sigma_M + \varDelta\sigma_{Hall-petch} + \Delta\sigma_{SS} + \Delta\sigma_{DS} + \varDelta\sigma_{LT} \quad (1)$$

where σ_C is the yield strength of composite, σ_M is yield strength of matrix. The difference between them can be attributed to the Hall-petch strengthening $\varDelta\sigma_{Hall\text{-}petch}$, solid solution strengthening $\varDelta\sigma_{SS}$, dislocation strengthening $\varDelta\sigma_{DS}$, and load transfer strengthening $\varDelta\sigma_{LT}$.

8.1 Hall-petch strengthening

Hall-Petch strengthening, also known as grain boundary strengthening, originates from the hindrance effect of grain boundaries on dislocation slip and propagation. The quantitative contribution of Hall-petch strengthening can be calculated by the classical Hall-petch relationship[40]:

$$\varDelta\sigma_{Hall-petch} = K\left(d_C^{-0.5} - d_M^{-0.5}\right) \quad (2)$$

where K is the Hall-Petch slope constant for pure Ni, with a value of 154.95 MPa·μm$^{0.5}$, d_C and d_M are the average grain size of composite and Ni matrix respectively. This negligible, even negative contribution is attributed to the ultra-low loading content of graphene in the composite, which only induces local grain refinement in limited regions, while the overall average grain size of the Ni matrix in the composite shows no significant refinement.

8.2 Solid solution strengthening

Solid solution strengthening arises from the lattice distortion induced by the dissolution of solute atoms into the metal matrix lattice. Solute atoms with a different atomic radius from the matrix atoms generate a local elastic strain field around their lattice sites, which interacts with moving dislocations, impedes dislocation glide, and thus improves the strength of the material. It can be calculated by the following equation:

$$\Delta\sigma_{ss} = MG\varepsilon^{\frac{3}{2}}\sqrt{\frac{f_m}{3}} \quad (3)$$

where M is the Taylor factor for Ni, taken as 3.1, G is the shear modulus of pure Ni, with a value of 65 GPa, ε is the lattice misfit parameter caused by interstitial carbon atoms in the Ni lattice, with a value of 0.0152[41]. f_m is the mass concentration of carbon dissolved in the Ni matrix, which is estimated according to the equilibrium solid solubility limit of carbon in Ni at room temperature (1×10^{-6} wt.%)[42]. Based on the above parameters, the calculated contribution of solid solution strengthening is ~0.21 MPa, which could be ignored.

8.3 Dislocation strengthening

The quantitative contribution of dislocation density can be calculated by the classic Taylor equation[41]:

$$\Delta\sigma_{ds} = \alpha MGb\sqrt{\rho_0} \tag{4}$$

where α is a dimensionless constant related to dislocation interaction, taken as 0.25, M is the Taylor factor (3.1), G is the shear modulus of Ni (65 GPa), b is the Burgers vector of Ni, with a value of 0.249 nm[43]. Dislocation density ρ_0 can be estimated as:

$$\rho_0 = \frac{2\sqrt{3}}{3}\frac{\theta}{bs} \tag{5}$$

where θ is the average misorientation angle per unit step length obtained from KAM mapping, and s is the step size of the EBSD scanning. Based on the experimental KAM data and the above parameters, the calculated contribution of dislocation strengthening is 13.9 MPa.

8.4 Load transfer strengthening

Load transfer strengthening is the most characteristic and dominant strengthening mechanism in graphene-reinforced metal matrix composites. The quantitative contribution of load transfer strengthening can be evaluated by different methods. In the shear-lag model, it is described as[44]:

$$\Delta\sigma_{LT} = \frac{S}{4} f_v \sigma_{YM} \tag{6}$$

where S is defined as the aspect ratio of the reinforcing phase. For the graphene reinforcement in our composite system, its in-plane lateral diameter can be approximated as 100 μm based on the characteristic feature size of the gyroid skeleton, and the thickness of the graphene nanosheets is determined to be 100 nm. The term f_v refers to the volume fraction of graphene (0.095 vol%). Using the above parameters, the load transfer strengthening is calculated to be 29.6 MPa.

8.5 Load transfer strengthening mechanism of 3D graphene network

Load transfer strengthening is identified as the dominant strengthening mechanism in the composite (Ni/PMMA-Lattice-HP), contributing 29.6 MPa to the total yield strength increment as calculated by the modified shear-lag model. It accounts for more than 70% of the total theoretically calculated strengthening effect. However, the classical shear-lag model adopted for theoretical calculation is established based on the core assumption of discrete, randomly distributed 2D graphene nanosheets in the metal matrix, which fundamentally differs from the 3D continuous, interpenetrating graphene network architecture in our composite system. The unique topological structure of the in-situ grown 3D graphene network enables a

more efficient load transfer behavior, which is the core reason for the underestimation of the load transfer strengthening contribution by the traditional theoretical model, as well as the gap between the total calculated strength increment (41.2 MPa) and the experimental value (53.8 MPa), as shown in **Supplementary Fig. 17**.

The primary advantage of the 3D graphene network in load transfer lies in its ability to form a robust mechanical interlocking structure with the Ni matrix, which significantly enhances the interfacial shear strength and the efficiency of stress transfer from the soft metal matrix to the high-strength graphene reinforcement. Molecular dynamics simulation results from Yang et al.[45] demonstrated that the interlocking structure formed by the 3D graphene network and the metal matrix improves the deformation compatibility between the reinforcement and the matrix, and the average interfacial pull-out force of the 3D graphene network in the Ni matrix is approximately three times higher than that of discrete 2D graphene sheets. In this work, the in-situ grown graphene conformally wraps the 3D printed gyroid Ni skeleton, forming an intimate Ni-graphene interface. This conformal growth further consolidates the mechanical interlocking between the 3D graphene network and the Ni matrix, ensuring that the external load applied to the composite can be efficiently and continuously transferred to the graphene reinforcement throughout the bulk material, rather than being limited to the local shear zone around individual graphene flakes.

Unlike discrete 2D graphene sheets that rely solely on interfacial shear stress to achieve load transfer, the 3D continuous graphene network can directly bear the external load through its integral skeleton structure, achieving long-range, full-scale load sharing in the composite. For conventional Gr-MMCs with discrete flake reinforcements, the load transfer process is restricted to the short-range shear zone at the graphene-matrix interface, and the reinforcing effect is severely limited by the in-plane size of individual graphene flakes; once interfacial debonding or graphene pull-out occurs, the load transfer capacity of the reinforcement is almost completely lost[44]. In contrast, the 3D graphene network in our composite forms a continuous, self-supporting reinforcing skeleton that interpenetrates with the Ni matrix. Even if local interfacial separation occurs at partial regions during tensile deformation, the integral network structure can still maintain the load-bearing capacity through the continuous interconnected skeleton, and exert an extrinsic toughening effect via crack bridging, which further amplifies the load transfer strengthening effect. Yang et al.[7] also pointed out that the continuous network structure enables graphene to bear load directly in addition to interfacial load transfer, which effectively compensates for the strength reduction

caused by local interfacial separation, and the straight portions of the 3D graphene network exhibit a more efficient load-sharing capacity during tension.

Furthermore, the unique deformability of the 3D graphene network optimizes the stress distribution during composite deformation, further improving the stability and efficiency of the load transfer process. MD results revealed that the curved portions of the 3D graphene network can be straightened during tensile deformation, which enables the network to release local stress concentrations caused by dislocation blocking through overall structural deformation, avoiding the premature fracture of the reinforcement caused by local stress overloading[7]. This stress relaxation effect ensures that the load can be uniformly distributed on the 3D graphene network, maintaining the long-term effectiveness of the load transfer process during the entire plastic deformation stage. Meanwhile, the straightening of the curved portions of the graphene network enhances the mechanical interlocking between the network and the Ni matrix through dislocation-graphene interactions, which further increases the interfacial shear stress and the load-sharing ratio of the graphene reinforcement. In contrast, when dislocations encounter discrete 2D graphene sheets, stress concentration is prone to occur at the edges of the sheets, leading to dislocation penetration through the graphene or interfacial debonding, which significantly reduces the load transfer efficiency[46].

Corresponding discussion was also mentioned in main text (page 21, lines 317-324).

Remarkably, Ni/PMMA-Lattice-HP maintains superior strain hardening rates throughout deformation, indicating sustained dislocation storage and multiplication enabled by the 3D Ni-Gr interpenetrating configuration. This continuous hardening promotes extensive uniform elongation while effectively suppressing localized failure. It should be emphasized that the strength improvement in composite can be attributed to multiple strengthening mechanisms quantitatively, including grain refinement, dislocation strengthening, and load transfer, where the high load transfer efficiency originating from the 3D graphene network dominants, as detailed and calculated in Supplementary Note 8, and compared in **Supplementary Fig. 17**.

8. The strengthening and toughening mechanisms of the graphene reinforced Ni-matrix composite have to be discussed in-depth.

Reply:

We sincerely appreciate the reviewer's suggestion. The reviewer notes that the strengthening and toughening mechanisms require in-depth discussion. To respond, a quantitative analysis of strengthening components has been provided (see response to Comment 7 and the supplementary information). Here, the multi-scale synergistic toughening mechanisms enabled by the in-situ architectured 3D graphene network are further elaborated.

A key conceptual advance of this work is the decoupling of strengthening and toughening mechanisms, which is rarely achievable by conventional powder metallurgy routes. In traditional MMCs or Gr-MMCs, mechanisms such as solid-solute strengthening, precipitation hardening, Orowan dislocation looping, and classical toughening (graphene pull-out, grain bridging, crack deflection) are inherently coupled and often mutually exclude[47]. In contrast, the present 3D architectured graphene network — enabled by the layer-by-layer building process of LPBF and the in-situ chemical/physical reactions embedded in the process — allows strengthening to be primarily governed by the high-quality sp^2 carbon bonds and intimate Gr-Ni interface (quantified in the supplementary information), while toughening is independently activated by the pre-designed 3D architecture across multiple length scales. This decoupling is unique to the additive manufacturing approach and underpins the exceptional combination of strength and ductility.

The exceptional toughness of the Ni-Gr composite (282.4 $MJ \cdot m^{-3}$, 63.2% higher than unreinforced Ni) without sacrificing ductility originates from the coupling of extrinsic toughening at the macro/mesoscale and intrinsic toughening at the micro/atomic scale, both rooted in the continuous, interpenetrating 3D Gr network and the robust Gr-Ni interface constructed via in-situ LPBF.

The detailed analysis has been added to SI (pages 33-34, lines 425-465).

Supplementary Note 9. Toughening mechanisms of as-fabricated Gr-Ni composites

9.1 Mesoscale extrinsic toughening dominated by the continuous 3D Gr network

Unlike discrete graphene nanosheets that provide only localized toughening, the 3D interconnected Gr network conformally wrapping the Ni scaffold enables global regulation of crack propagation throughout the bulk composite. When a crack propagates, the continuous 3D Gr network acts as a persistent barrier,

forcing extensive crack deflection, branching, and twisting. This is directly evidenced by the highly tortuous crack path with abundant secondary and tertiary cracks on the fracture surface of the Ni/PMMA-Lattice-HP specimen. Such a tortuous path dramatically increases the total fracture length and the associated energy dissipation — a classic extrinsic toughening mechanism validated in Gr-MMCs[2,48]. More importantly, unlike discrete graphene flakes that induce only local deflection, the 3D Gr network suppresses the formation of catastrophic through-thickness cracks throughout the entire loading process, which is key to avoiding premature fracture and maintaining high ductility.

The robust semi-coherent Gr-Ni interface (**Supplementary Fig. 5d, e**) enables the continuous Gr network to form stable bridging ligaments in the crack wake, resisting crack opening and slowing propagation[47]. During final fracture, the as-grown graphene layers are gradually pulled out from the Ni matrix, as evidenced by abundant pulled-out and bent FLG fragments on the fracture surface (**Fig. 6a**). Notably, the 3D continuous network ensures that this crack-bridging and pull-out toughening effect operates across the entire fracture plane, not merely in local regions.

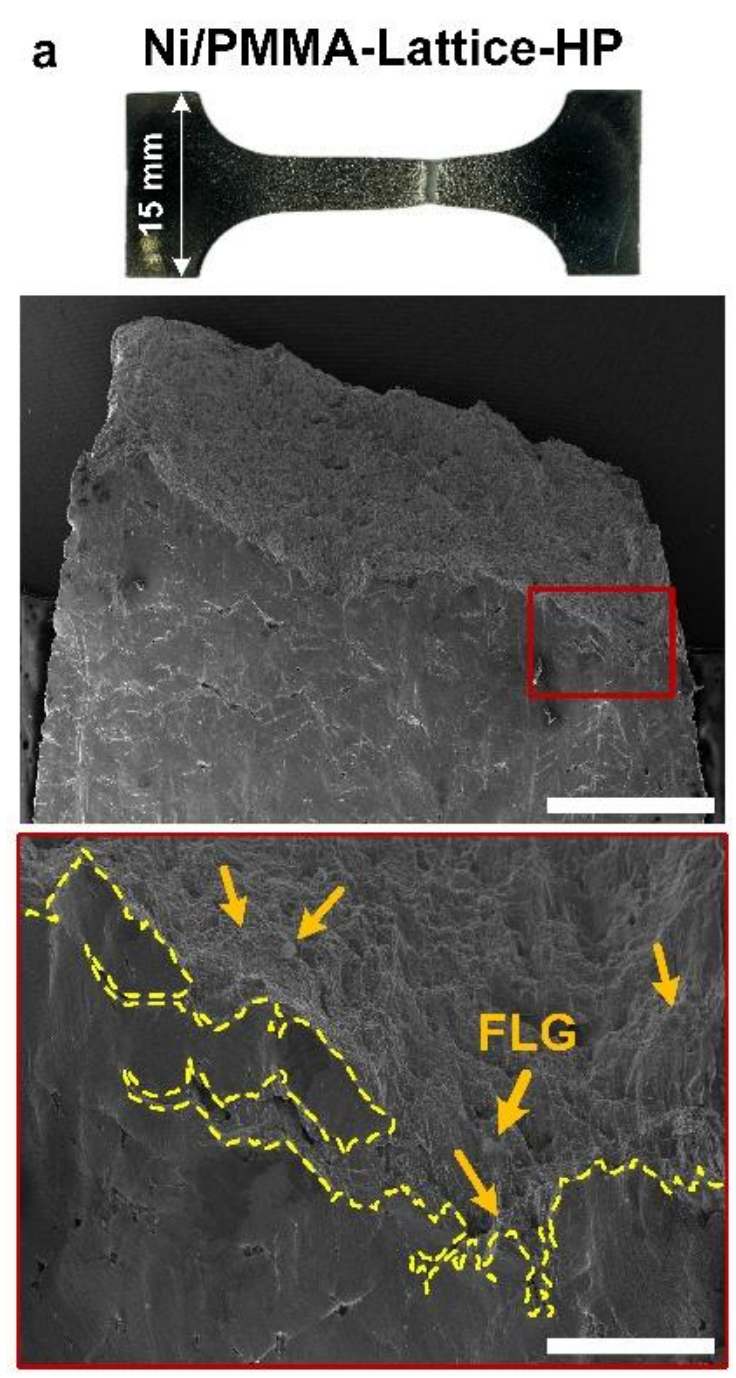

Fig. 6a OM and SEM images of tensile testing coupons and fracture surfaces of **a** Ni/PMMA-Lattice-HP composite.

9.2 Micro scale intrinsic toughening for ductility retention

The interpenetrating 3D Gr-Ni architecture creates a heterogeneous microstructure consisting of graphene-reinforced regions and pure Ni matrix regions with a large difference in strength and deformability. During tensile deformation, the mechanical incompatibility between these regions generates long-range back stress in the matrix. This back stress not only contributes to strength enhancement but also promotes sustained dislocation accumulation and multiplication throughout the deformation process[49,50]. Direct evidence is the maintained high strain hardening rate of the Ni/PMMA-Lattice-HP specimen until fracture, which effectively suppresses premature necking and enables extensive uniform plastic deformation, thereby retaining the excellent ductility of the pure Ni matrix. Further support comes from EBSD results of fractured samples (**Fig. 7**): the distribution of low-angle grain boundaries (LAGBs) in the Ni/PMMA-Lattice-HP sample clearly divides the material into two regions with distinct deformation levels. Meanwhile, the Ni matrix region retains a high dislocation storage capacity, underpinning subsequent plastic deformation and energy absorption.

Therefore, the combination of global extrinsic toughening (crack deflection/branching, bridging, pull-out) and intrinsic toughening (heterogeneous deformation-induced back stress, sustained strain hardening) explains the exceptional toughness enhancement. The decoupling of strengthening and toughening — strength from high-quality graphene and its interface, toughness from the 3D architecture — represents a paradigm enabled uniquely by the proposed LPBF-based in-situ synthesis strategy in this study.

Corresponding discussion was also mentioned in main text (pages 25-26, lines 388-390).

The combination of these intrinsic toughening mechanisms with extrinsic toughening via crack deflection represents a comprehensive strategy to overcome the strength-ductility trade-off in architectured MMCs (Supplementary Note 9).

References

1. Yazyev, O. V. Effect of Metal Elements in Catalytic Growth of Carbon Nanotubes. *Phys. Rev. Lett.* **100**, (2008).
2. Zhang, Y. *et al.* Bioinspired, graphene-enabled Ni composites with high strength and toughness. *Science Advances* **5**, eaav5577 (2019).
3. Kim, J., Son, S., Choe, M. & Lee, Z. *In situ* TEM investigation of nickel catalytic graphitization. *Materials Today Advances* **22**, 100494 (2024).
4. Lin, Z. *et al.* Enhanced strength and toughness of Ni/graphene composite via three-dimensional graphene-like nanosheets network. *Composites Part B: Engineering* **253**, 110499 (2023).
5. Dahal, A. & Batzill, M. Graphene–nickel interfaces: a review. *Nanoscale* **6**, 2548–2562 (2014).
6. Zhang, K., Surana, M., Haasch, R. & Tawfick, S. Elastic modulus scaling in graphene-metal composite nanoribbons. *J. Phys. D: Appl. Phys.* **53**, 185305 (2020).
7. Das, S., Lahiri, D., Lee, D.-Y., Agarwal, A. & Choi, W. Measurements of the adhesion energy of graphene to metallic substrates. *Carbon* **59**, 121–129 (2013).
8. Niilisk, A., Kozlova, J., Alles, H., Aarik, J. & Sammelselg, V. Raman characterization of stacking in multi-layer graphene grown on Ni. *Carbon* **98**, 658–665 (2016).
9. Chen, R. & Xu, M. Kinetic and volatile products study of micron-sized PMMA waste pyrolysis using thermogravimetry and Fourier transform infrared analysis. *Waste Management* **113**, 51–61 (2020).
10. Acosta, R. I., Gross, K. C. & Perram, G. P. Thermal degradation of Poly(methyl methacrylate) with a 1.064 μm Nd:YAG laser in a buoyant flow. *Polymer Degradation and Stability* **121**, 78–89 (2015).
11. King, A. A. K. *et al.* A New Raman Metric for the Characterisation of Graphene oxide and its Derivatives. *Sci Rep* **6**, 19491 (2016).

12. Ferrari, A. C. *et al.* Raman Spectrum of Graphene and Graphene Layers. *Phys. Rev. Lett.* **97**, 187401 (2006).

13. López-Díaz, D., López Holgado, M., García-Fierro, J. L. & Velázquez, M. M. Evolution of the Raman Spectrum with the Chemical Composition of Graphene Oxide. *J. Phys. Chem. C* **121**, 20489–20497 (2017).

14. Evolution of the Raman Spectrum with the Chemical Composition of Graphene Oxide. *ACS Publications* https://pubs.acs.org/doi/full/10.1021/acs.jpcc.7b06236 (2017).

15. Zhang, Y. & Li, X. Bioinspired, Graphene/Al2O3 Doubly Reinforced Aluminum Composites with High Strength and Toughness. *Nano Lett.* **17**, 6907–6915 (2017).

16. Stokbro, K., Engelund, M. & Blom, A. Atomic-scale model for the contact resistance of the nickel-graphene interface. *Phys. Rev. B* **85**, 165442 (2012).

17. Murzaev, R. T., Krylova, K. A. & Baimova, J. A. Thermal Expansion and Thermal Conductivity of Ni/Graphene Composite: Molecular Dynamics Simulation. *Materials* **16**, 3747 (2023).

18. Holzmond, O. & Li, X. In situ real time defect detection of 3D printed parts. *Additive Manufacturing* **17**, 135–142 (2017).

19. Bartlett, J. L., Heim, F. M., Murty, Y. V. & Li, X. *In situ* defect detection in selective laser melting via full-field infrared thermography. *Additive Manufacturing* **24**, 595–605 (2018).

20. Bartlett, J. L., Croom, B. P., Burdick, J., Henkel, D. & Li, X. Revealing mechanisms of residual stress development in additive manufacturing via digital image correlation. *Additive Manufacturing* **22**, 1–12 (2018).

21. Bartlett, J. L. & Li, X. An overview of residual stresses in metal powder bed fusion. *Additive Manufacturing* **27**, 131–149 (2019).

22. Yin, J. *et al.* A finite element model of thermal evolution in laser micro sintering. *Int J Adv Manuf Technol* **83**, 1847–1859 (2016).

23. Wei, Z., Ni, Z., Bi, K., Chen, M. & Chen, Y. In-plane lattice thermal conductivities of multilayer graphene films. *Carbon* **49**, 2653–2658 (2011).

24. Baraton, L. *et al.* On the mechanisms of precipitation of graphene on nickel thin films. *EPL* **96**, 46003 (2011).

25. Wu, P., Zhang, W., Li, Z. & Yang, J. Mechanisms of Graphene Growth on Metal Surfaces: Theoretical Perspectives. https://doi.org/10.1002/smll.201303680 doi:10.1002/smll.201303680.

26. Yang, Q., Zhang, Z., Zhu, W. & Wang, G. Growth of Large-Area High-Quality Graphene on Different Types of Copper Foil Preannealed under Positive Pressure H2 Ambience. *ACS Omega* **4**, 5165–5171 (2019).

27. Cabrero-Vilatela, A., Weatherup, R. S., Braeuninger-Weimer, P., Caneva, S. & Hofmann, S. Towards a general growth model for graphene CVD on transition metal catalysts. *Nanoscale* **8**, 2149–2158 (2016).

28. Qiu, C. & Metselaar, R. Solubility of carbon in liquid Al and stability of Al4C3. *Journal of Alloys and Compounds* **216**, 55–60 (1994).

29. Fang, W., Hsu, A. L., Song, Y. & Kong, J. A review of large-area bilayer graphene synthesis by chemical vapor deposition. *Nanoscale* **7**, 20335–20351 (2015).

30. Zhang, G. *et al.* In situ synthesized 3D network structure TC4 metal matrix composites reinforced by the graphene via using laser additive manufacturing. *Journal of Materials Research and Technology* **39**, 4020–4032 (2025).

31. Weatherup, R. S., Dlubak, B. & Hofmann, S. Kinetic Control of Catalytic CVD for High-Quality Graphene at Low Temperatures. *ACS Nano* **6**, 9996–10003 (2012).

32. Patera, L. L. *et al.* Real-time imaging of adatom-promoted graphene growth on nickel. *Science* **359**, 1243–1246 (2018).

33. Cao, M. *et al.* Ultrahigh Electrical Conductivity of Graphene Embedded in Metals. *Advanced Functional Materials* **29**, 1806792 (2019).

34. Yue, T. *et al.* Effects of volumetric energy density on melting modes, printability, microstructures, and mechanical properties of laser powder bed fusion (L-PBF) printed pure nickel. *Materials Science and Engineering: A* **909**, 146871 (2024).

35. Hu, H. *et al.* A comparison study of high purity nickel fabricated by laser powder bed fusion and subjected to dynamic plastic deformation. *Materialia* **33**, 102044 (2024).

36. Güler, Ö. & Bağcı, N. A short review on mechanical properties of graphene reinforced metal matrix composites. *Journal of Materials Research and Technology* **9**, 6808–6833 (2020).

37. Chen, Z. *et al.* Graphene reinforced nickel-based superalloy composites fabricated by additive manufacturing. *Materials Science and Engineering: A* **769**, 138484 (2020).

38. Zhang, X. *et al.* A powder-metallurgy-based strategy toward three-dimensional graphene-like network for reinforcing copper matrix composites. *Nat Commun* **11**, 2775 (2020).

39. Launey, M. E. & Ritchie, R. O. On the Fracture Toughness of Advanced Materials. *Advanced Materials* **21**, 2103–2110 (2009).

40. Armstrong, R. W. Hall–Petch description of nanopolycrystalline Cu, Ni and Al strength levels and strain rate sensitivities. *Philosophical Magazine* **96**, 3097–3108 (2016).

41. Arya, A. *et al.* Strength and microstructure evolution in nickel during large strain wire drawing. *Acta Materialia* **221**, 117396 (2021).

42. Lander, J. J., Kern, H. E. & Beach, A. L. Solubility and Diffusion Coefficient of Carbon in Nickel: Reaction Rates of Nickel-Carbon Alloys with Barium Oxide. *Journal of Applied Physics* **23**, 1305–1309 (1952).
43. Li, Z., Gu, H., Luo, K., Kong, C. & Yu, H. Achieving High Strength and Tensile Ductility in Pure Nickel by Cryorolling with Subsequent Low-Temperature Short-Time Annealing. *Engineering* **33**, 190–203 (2024).
44. Ryu, H. J., Cha, S. I. & Hong, S. H. Generalized shear-lag model for load transfer in SiC/Al metal-matrix composites. *Journal of Materials Research* **18**, 2851–2858 (2003).
45. Yang, Y. *et al.* Strengthening behaviour of continuous graphene network in metal matrix composites. *Carbon* **182**, 825–836 (2021).
46. Shuang, F. & Aifantis, K. E. Relating the strength of graphene/metal composites to the graphene orientation and position. *Scripta Materialia* **181**, 70–75 (2020).
47. Ritchie, R. O. The conflicts between strength and toughness. *Nature Mater* **10**, 817–822 (2011).
48. Zhang, M. *et al.* 3D printed Mg-NiTi interpenetrating-phase composites with high strength, damping capacity, and energy absorption efficiency. *Science Advances* **6**, eaba5581 (2020).
49. Li, Z. *et al.* Enhanced Mechanical Properties of Graphene (Reduced Graphene Oxide)/Aluminum Composites with a Bioinspired Nanolaminated Structure. *Nano Lett.* **15**, 8077–8083 (2015).
50. Lin, Z. *et al.* Graphene-decorated bimodal pure metal with high strength and ductility. *Materials Research Letters* **12**, 815–824 (2024).

Response to the reviewers

Ref: NCOMMS-25-100125A

Title: In-situ Architecturing of 3D Graphene Networks in Metals via Laser Additive Manufacturing for Strength-Toughness Synergy

Journal: Nature Communications

Reviewer #1 (Remarks to the Author):

The revised manuscript is of higher quality. Publish as is.

Reviewer #2 (Remarks to the Author):

I have reviewed the revised manuscript and recommend its acceptance for publication. The authors have properly addressed all the previous comments, and the manuscript meets the journal's publication criteria.

Response:

We are grateful for the opportunity to revise our manuscript and appreciate the reviewers' time and constructive feedback. We are pleased to see that both reviewers have found the revised manuscript acceptable for publication.

We thank the reviewers for their thorough evaluation and valuable suggestions, which have significantly improved the quality of our work.
